# Investigation of Sulfur-related Influences on the Construction and Ring Opening of a Six-membered Cyclic Xanthate Derived from d‑Thymidine and CS_2_

**DOI:** 10.1021/jacs.6c15290

**Published:** 2026-10-05

**Authors:** Autumn M. Andras, Nehal Fatima, Kai-Hua Mick Kuo, David K. Tran, Donald J. Darensbourg, Karen L. Wooley

**Affiliations:** † Departments of Chemistry, 14736 Texas A&M University, College Station, Texas 77843, United States; ‡ Materials Science & Engineering, 14736 Texas A&M University, College Station, Texas 77843, United States

## Abstract

With an interest in sustainable polymers derived from nucleoside building blocks, d-thymidine and carbon disulfide (CS_2_) were used as synthetic precursors to construct a *trans*-fused, 6,5-membered bicyclic xanthate, 3′,5′-cyclic *N* ^3^-(3-butenyl)-d-thymidine *trans* xanthate, for ring-opening polymerization (ROP) to yield stereo- and regiochemically defined homopolymer analogs of nucleic acids bearing thiocarbonate backbones. Screenings for CS_2_ insertion and succeeding cyclization to form the targeted monomer revealed two additional d-thymidine-derived cyclic moieties, including a xanthate and trithiocarbonate of *cis*-fused configurations, isolated in low yields. Therefore, ROP experiments using alkoxy-based initiators and organobase catalysts at room temperature and supporting DFT calculations for enthalpies of ring opening were extended to all three potential monomers, with only the *trans*-fused bicyclic xanthate undergoing ROP. Structural characterization by MALDI-TOF mass spectrometry and NMR spectroscopy of polymers obtained from ROPs of 3′,5′-cyclic *N* ^3^-(3-butenyl)-d-thymidine *trans* xanthate conducted at or somewhat below ambient temperatures indicated the presence of linear and cyclic species having primarily dimeric 5′,5′-trithiocarbonate-*co*-3′,3′-thionocarbonate connectivities from an alternating C–S and C–O cleavage pathway. Other minor trithiocarbonate, xanthate, and thionocarbonate functionalities were proposed to arise from backbiting reactions and/or minor regiorandom cleavages. In contrast, ROPs conducted at reduced temperatures (≤ −20 or −40 °C depending on the organobase) achieved selective C–S cleavage to yield 3′,5′-xanthate-connected polymers of *N* ^3^-(3-butenyl)-d-thymidine with more structural similarity to native DNA. These fundamental advances extend the breadth of nucleoside-derived polymers, emphasizing the influence of sulfur incorporation during monomer synthesis and upon subsequent ROPs, with outcomes being temperature-mediated alternating vs. unidirectional backbone compositional and structural regioregularity.

## Graphical abstract

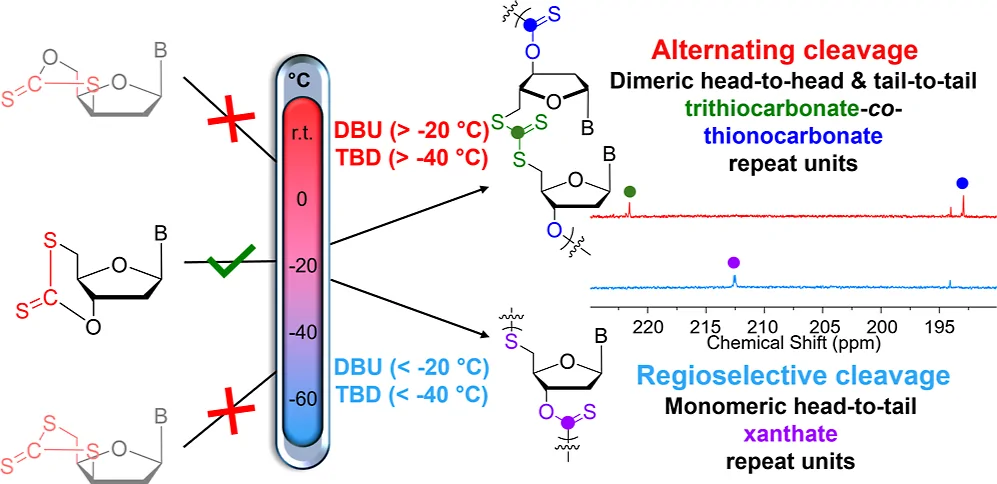

## Introduction

Deoxyribonucleic acid (DNA) and ribonucleic acid (RNA) are remarkably sophisticated biomacromolecules that perform essential, life-sustaining functions, enabled by their ordered molecular architectures. Consequently, there has been sustained interest over several decades in developing synthetic methodologies to access biomimetic analogs for clinical^1^ and other applications, which rely on the use of nucleosides and/or their structural components. Conventional approaches to the generation of nucleic acid analogs for therapeutics implement predominantly solid-phase syntheses, wherein nucleoside-derived reactive intermediates, such as phosphoramidites^2,3^ and phosphodiesters,^4^ undergo coupling and subsequent oxidation and/or sulfurization reactions to afford sequence-defined oligonucleotides with P­(V)-based backbones.^5–8^ Biocatalytic approaches have also emerged for more stream-lined production, extending to high chain lengths and large scales.^9^ In contrast, nucleic-acid-mimicking synthetic polymers for materials applications largely forfeit the structural similarity of native DNA. Instead, they often focus on incorporation of nucleosides or nucleobases as pendant groups upon petrochemically sourced backbones,^10,11^ with interest in utilizing their intrinsic noncovalent interactions, such as hydrogen bonding and pi–pi interactions,^12–14^ in various applications, including adhesives,^15^ supramolecular materials/nanoparticles,^16–18^ and solid polymer electrolytes for batteries^19^ or other applications. These synthetic methods have included polymerizations through either step-growth, condensation (ADMET),^19^ or chain-growth (ROMP, RAFT, ATRP)^20,21^ chemistries, in which nucleoside/nucleobase functionalities were preinstalled on the monomer or added post polymerization.

Despite this long-standing history, utilizing nucleosides as direct building blocks for scalable and rapid construction of synthetic polymers with expansion of the chemical functionality of nucleotide connectivity remains underdeveloped. Such polymers constitute a class of materials that may allow for sought-after, efficient noncovalent interactions through the establishment of regioregular backbones and well-defined stereochemistries while also employing naturally derived monomer building blocks for expansion of sustainable and functional polymeric materials. One promising synthetic strategy to achieve this chemistry is chain-growth, ring-opening polymerization (ROP) of nucleoside-derived cyclic monomers.^22–25^ This approach relies upon chemistries at the 3′–OH and 5′–OH positions that allow for the formation of fused, six-membered cyclic structures, which can undergo facile ROPs for chain elongation, thereby bypassing the requirement for continuous protection and deprotection chemistries. Over a similar timeframe nearly a decade ago, such processes were demonstrated for polyphosphotriesters, polycarbonates, and polythioesters. Our group established a two-step methodology to generate well-defined polyphosphoesters with primarily 3′,5′-backbone connectivities.^24,25^ Expanding beyond phosphorous-containing backbones, work by Buchard and coworkers^22^ featured the formation of a *cis*-fused, six-membered cyclic carbonate by insertion of carbon dioxide (CO_2_) into the 1,3-diol moiety of d-thymidine. Their findings indicated that the generation of the *trans*-fused isomer was not thermodynamically favorable, likely due to the resulting shorter C–O bond lengths. Consequently, the polymer after ROP of the *cis* isomer exhibited both differing stereo- and regiochemical outcomes from their native DNA counterpart. Finally, Bowman and coworkers^23^ reported the inclusion of sulfur into the backbone of nucleoside-derived polymers from the ROP of d-thymidine and deoxycytidine thiolactones as a platform to develop regioregular polymers capable of template-directed synthesis through thiol—thioester exchange reactions.

Inspired by the works mentioned above and the increased interest in sulfur incorporation for sustainable polymeric materials,^26,27^ we sought to expand the scope of nucleoside-derived polymers generated by ROP through employment of a sulfur-containing cyclizing agent, carbon disulfide (CS_2_). Motivation for employing CS_2_ included addressing the inability to isolate the *trans*-fused cyclic carbonate isomer^22^ by the generation of a longer C–S bond. This approach would alleviate some ring strain exhibited by the carbonate analog, allowing for the formation and isolation of a six-membered, *trans*-fused cyclic xanthate monomer to facilitate ROP. Further, incorporation of a more labile C–S bond compared to C–O would create both an opportunity to impart a directional preference for ring opening, thereby resolving the regioirregularity in the polycarbonate analog and aiding in future depolymerization/degradation.^23,26,28,29^ Here, synthetic efforts to generate the cyclic xanthate monomer derived from d-thymidine and CS_2_ and its ROP to gain regioselective control during propagation are described.

## Results and Discussion

Monomer development to produce the target 3′,5′-*trans*-fused, six-membered cyclic xanthate derived from d-thymidine comprising a 6,5-bicyclic monomer (**1**) relied on a similar synthetic strategy previously reported by our group but encountered several unique challenges.^24,25^ In that approach, a butenyl moiety was first installed at the *N* ^3^-position on the nitrogenous base for protection, solubility, and functionalization purposes, leaving the 3′- and 5′-positions open for cyclization via the reaction with ethyl dichlorophosphate and establishing a butenyl-functionalizeḑ *trans*-fused, 6,5-membered bicyclic phosphotriester. Likewise, in this work, butenylation at the *N* ^3^-position was performed by reaction with 4-bromo-1-butene in the presence of potassium carbonate at 60 °C for 24 h to afford **2** in 81% yield (Scheme 1). Efforts were then dedicated to the establishment of the cyclic xanthate as opposed to the previous incorporation of a phosphotriester functionality through the 3′ and 5′ positions with control over the regiochemistry and maintenance of the *trans* stereochemistry.

**Scheme 1:**
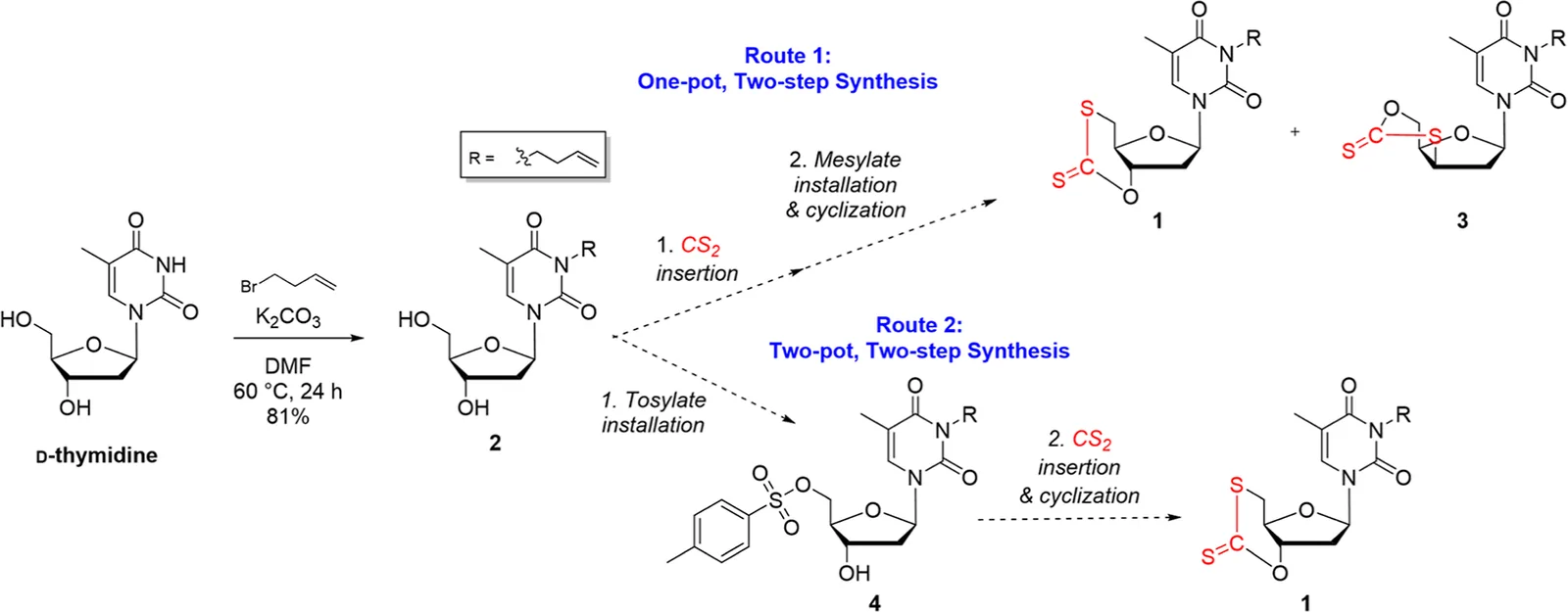
Two Established Synthetic Routes Investigated for the Preparation of d-Thymidine-Derived Cyclic Xanthates

Cyclization-based insertion of CS_2_ to produce **1** was investigated via two established methodologies, including either one-pot, two-step, or two-pot, two-step chemistries (Scheme 1). The one-pot, two-step strategy follows a synthetic method reported by Buchard et al.^30^ for six-membered cyclic xanthate formation from 1,3-diols of both deoxyribose and d-xylose, giving 6,5-bicyclic and 6,5,5-tricyclic monomers possessing *trans*- and *cis*-stereochemistries, respectively, relative to the 3- and 5- positions. Those syntheses included the insertion of CS_2_ at either hydroxyl in the presence of 1,8-diazabicycloundec-7-ene (DBU) to generate nucleophilic xanthate anions, followed by addition of mesityl chloride and triethylamine to accomplish in situ formation of mesylate leaving groups, and thereafter allowing for intramolecular cyclizations, albeit in limited yields (≤15%). As we considered this approach, it was recognized that mechanistically for the d-thymidine system, this process had two limitations: (1) the presence of two hydroxyl groups could result in a mixture of regioisomers (**1** and **3**), and (2) nucleophilic attack of a 5′-xanthate anion intermediate at the stereogenic 3′-center would result in the unintended regioisomer also possessing undesired *cis*-fused cyclic xanthate stereochemistry (**3**). Of note, when applied to d-xylose, this method had previously generated a *cis*-fused cyclic thionocarbonate from an addition/elimination pathway in significant yield (48%).^30^ However, formation of a cyclic thionocarbonate for the d-thymidine analog of this current work was not expected due to the requirement that it would accommodate *trans*-stereochemistry with relatively shorter C–O bond lengths; indeed, Buchard had demonstrated that cyclic carbonates of d-thymidine^22^ could not accommodate a *trans*-fused configuration. In our hands, non-optimized one-pot, two-step attempts led to only low yields of isomers **1** and **3**.

Structural elucidation of both isomers was achieved by employment of 1D and 2D nuclear magnetic resonance (NMR) spectroscopic techniques (Figure 1). Specifically, chemical shifts of ^13^C and ^1^H NMR signals were used to confirm the formation of two separate cyclic xanthates and determine their regiochemistries, while stereochemical assignments relied upon 2D nuclear Overhauser effect spectroscopy (NOESY) analyses. ^13^C NMR signals of thiocarbonyl carbons resonate downfield relative to their oxygen counterparts,^31,32^ with literature-reported signals of six-membered cyclic xanthate thiocarbonyl carbons resonating between ca. 206 ppm–208 ppm.^30,33,34^ Therefore, the presence of ^13^C NMR signals at 206.8 and 207.4 ppm corroborate the formation of two cyclic xanthate species, which were assigned to the thiocarbonyls of **1** and **3**, respectively, based on further analysis of resonance frequencies for carbons and protons in the 3′ and 5′ positions. Regiochemical arrangements for the nonthiocarbonyl S and O atoms of the xanthate moieties at the C3′- and C5′-positions were based on the weaker inductive effect of S, which results in carbons with direct S vs O connectivities to resonate further upfield. The ^13^C NMR spectrum of **1** displays signals at 82.6 and 36.5 ppm for C3′ and C5′, respectively, indicating an O-bonded C3′ and an S-bonded C5′ (Figure 1A). Conversely, for **3**, the chemical shifts for C3′ and C5′ at 44.1 and 72.8 ppm, respectively, support an S-bonded C3′ and an O-bonded C5′ (Figure 1B). The same trend in chemical shift values of the signals for the corresponding protons, H_c_ and H_(e,e’)_, is observed in the ^1^H NMR spectrum of each isomer. Finally, stereochemical assignments of the stereogenic C3′ centers were deduced by determining the extent of through-space interactions between H_c_ with H_d_ and H_(e,e’)_ protons through examining the relative intensities of their cross-correlation peaks in each NOESY spectrum. The NOESY spectrum of **1** displays only a minor cross-peak at (4.94 ppm, 4.14 ppm), resulting from the weak interaction between H_c_ and H_d_, while a strong cross-peak at (4.94 ppm, 3.44 ppm) arises from the strong interaction between H_c_ and H_e_ (Figure 1A). Collectively, these signals support the *trans*-fusion of the six-membered cyclic xanthate ring to the five-membered sugar core of d-thymidine. In comparison, the NOESY spectrum cross-peak at (3.92 ppm, 4.67 ppm) for significant H_c_–H_d_ interaction and an absence of H_c_–H_e_ and H_c_–H_e’_ cross-peaks at (3.92, 4.44, and 3.92 ppm, 4.89 ppm) confirm the *cis*-fused configuration of **3** (Figure 1B).

**Figure 1:**
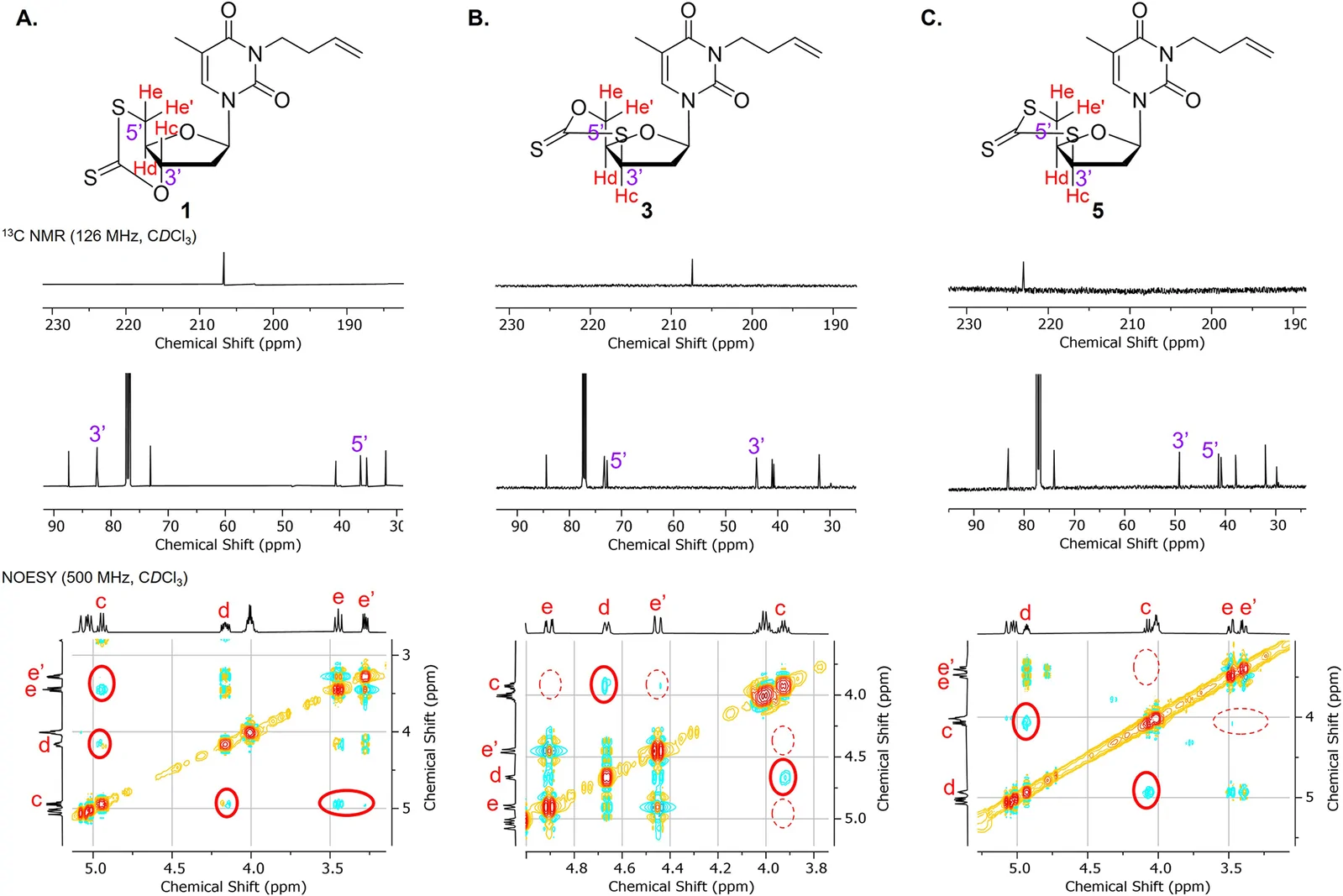
^13^C NMR (126 MHz, CDCl_3_) and NOESY (500 MHz, CDCl_3_) spectra of **1**, **3**, and **5**.

Efforts were then redirected to employing the two-pot, two-step method (Schemes 1 and 2). Buchard et al.^33,34^ had applied this approach of preinstallation of a tosylate leaving group followed by CS_2_ insertion and subsequent intramolecular cyclization for d-glucal derivatives, which retained the native *trans* stereochemistry of the sugar and afforded 6,6-bicyclic xanthate monomers in good yield. Zhou et al.^35^ followed similar chemistry for insertion of carbonyl sulfide (COS) into d-xylose derivatives to generate 6,5,5-tricyclic monothiocarbonate monomers. Applied to **2**, monotosylation at the 5′–OH was executed by reaction with *p*-toluenesulfonyl chloride in pyridine at room temperature, giving **4**. In the presence of DBU,^30,35^ reaction of **4** with CS_2_ at room temperature gave no isolable amount of **1** but led to complex oligomeric mixtures, which may have included uncontrolled ring-opening of **1** in situ. In addition, an unforeseen *cis*-fused, six-membered cyclic trithiocarbonate, **5**, was observed (Scheme 2) and underwent similar characterization experiments as for **1** and **3** (Figure 1C). Formation of the trithiocarbonate moiety was based on the appearance of a characteristic ^13^C NMR signal at 223.2 ppm and further corroborated by the upfield chemical shifts of both C3′ and C5′ carbons (49.1 and 41.4 ppm, respectively) and their respective protons, H_c_ and H_(e,e’)_, due to the two adjacent nonthiocarbonyl S atoms. Finally, similar to **3**, the NOESY spectrum indicated a *cis* configuration through the appearance of a strong cross-correlation peak at (4.08 ppm, 4.94 ppm) for significant H_c_–H_d_ interaction, while basically no correlation is observed for the H_c_–H_e_ or H_c_–H_e’_ interaction (Figure 1C). There are several potential mechanistic pathways by which to arrive at the cyclic trithiocarbonate structure from **4**, each complicated and likely involving reaction pathways known for DBU with CS_2_,^36–39^ as supported by the reaction mixture briefly becoming red immediately. Alternatively, the possibility for the direct transformation of **1** to **5** was tested by running a small-scale polymerization of **1** in the presence of 4-methylbenzyl alcohol, DBU, and CS_2_. Mass spectrometry analysis suggested formation of **5** in low quantity, leading to a proposed mechanism detailed in the Supporting Information (Scheme S1). As such, this transformation may also transpire during the DBU-facilitated two-step, two-pot cyclization strategy through a similar mechanism with ring opening of **1** presumably initiated by unreacted **4**. Additional screenings using DBU at lower temperatures and in various solvents also proved to be unsuccessful, with either no improvement for selective formation of **1** or inhibition of reactivity altogether. Alternatively, in the presence of *t*-BuOK at 0 °C,^33^ formation of **1** was detected with minimal formation of oligomeric species and no isolation of **5**. However, conversion of **4** to **1** proceeded exceptionally slowly, reaching less than ∼50% conversion in 48 h, as determined by ^1^H NMR spectroscopy. Therefore, after addition of *t*-BuOK and CS_2_ at 0 °C, the reaction mixture was warmed to 20 °C. Under these conditions, the reaction reached ∼89% conversion of **4** in 6 h with a ∼60% NMR yield, but with difficult purification due to an inseparable, unknown byproduct. Instead, addition of reagents at −78 °C and subsequent warming to 20 °C allowed for ∼99% conversion of **4** over a total period of 5 h with a ∼67% NMR yield and an isolated yield of 58% of pure **1** (Scheme 2).

**Scheme 2:**
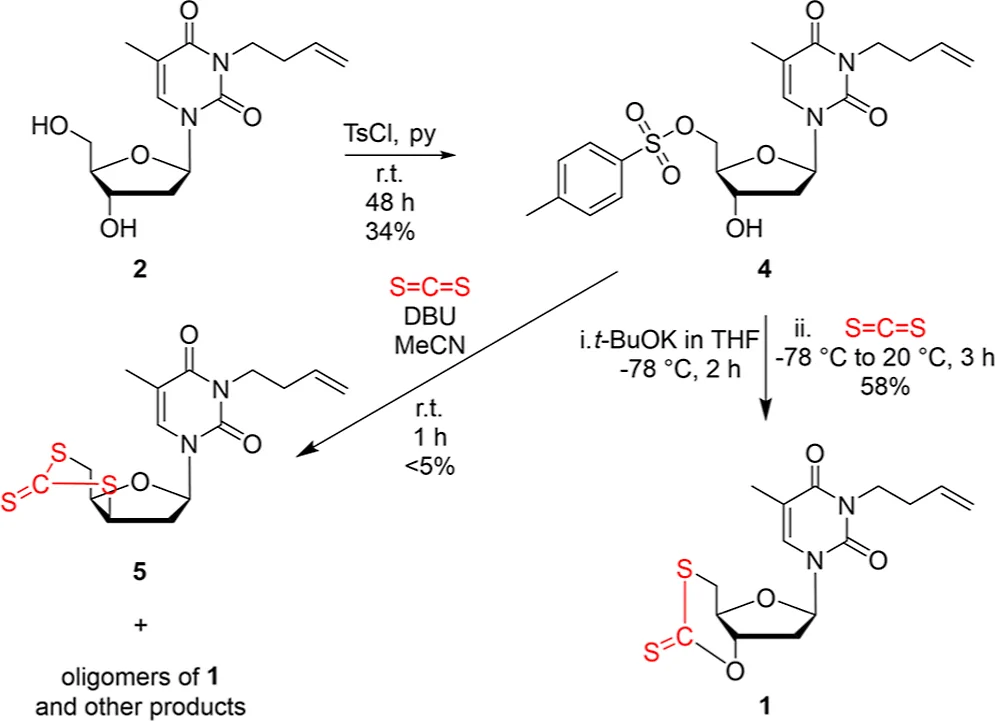
Optimization of Conditions to Avoid Side Reactions and Achieve Synthesis of 1 by a Two-Pot, Two-Step Approach from **2**

The abilities of **1**, **3**, and **5** to undergo ROPs were investigated experimentally and then evaluated computationally. Experimental screenings for their ROPs utilized organobase catalyst/initiator systems by employing DBU or 1,5,7-triazabicyclo[4.4.0]­dec-5-ene (TBD) with substituted benzyl alcohol initiators at ambient/room temperature (r.t.). Under these conditions, only **1** underwent ROP (Figure 2A), which was in agreement with computational results derived from density functional theory (DFT) calculations evaluating the enthalpies of ring opening (i.e., ring strain) for each monomer. To establish a reliable thermodynamic baseline for polymerizability, the ring strain energies of a series of model compounds were obtained by calculating the enthalpy of ring opening using an isodesmic approach (Figures S23 and S24 and Table S1). This approach^22,40^ utilizes the fragment structures of the actual polymer backbone, which is hypothesized to better reflect real-world ring-opening behavior compared to other methods.^24,41^ To ensure an accurate representation of the macroscopic thermodynamic properties for both cyclic and acyclic molecules, ensemble averages of different conformers at room temperature were carried out (see Supporting Information). The results successfully captured the relative trends expected from the literature: monomers known to readily undergo ROP exhibited significantly higher ring strain than their non-polymerizable counterparts. For instance, the polymerizable trimethylene cyclic carbonate^42^ and 7-membered trithiocarbonate^43^ displayed substantial ring strains, whereas the non-polymerizable analogs, ethylene carbonate^44^ and trimethylene trithiocarbonate^43^ yielded markedly lower values (Figures 3 and S23 **structures a–e**, and Table S1). Notably, the calculated ring strains of the trimethylene cyclic carbonate, trithiocarbonate, and xanthate analogs exhibit a qualitative trend wherein the substitution of oxygen with larger sulfur atoms within a given ring size reduces the overall ring strain. This result aligns with earlier structural and computational investigations by Darensbourg and coworkers for five,six-membered bicyclic carbonate structures containing 0-to-3 sulfur atoms.^45^ Applying this established thermodynamic rationale to the nucleoside derivatives (**1**, **3**, and **5**) and a previous study (d-thymidine 3′,5′-*cis*-carbonate, **f**, reported by Buchard et al.)^22^ (Figures 3 and S23 **structures 1**, **3**, **5**, and **f**, respectively, and Table S1) yielded excellent agreement with the experimental ROP outcomes. Both the polymerizable *cis*-fused carbonate reference **f** and the targeted *trans*-fused xanthate **1** possess high ring strains of 8.14 and 7.67 kcal/mol, respectively, placing them well within the thermodynamically favorable zone to drive chain propagation. In contrast, the experimentally inactive *cis*-fused trithiocarbonate **5** lacks meaningful ring strain (1.21 kcal/mol), thermodynamically precluding its polymerization. Notably, while the *cis*-fused xanthate **3** exhibits a moderate ring strain of 5.55 kcal/mol, it sits approximately 2 kcal/mol lower than its *trans* counterpart regardless of the computational methods being used, remaining below the critical activation threshold. Therefore, only ROP of **1** was further pursued.

**Figure 2:**
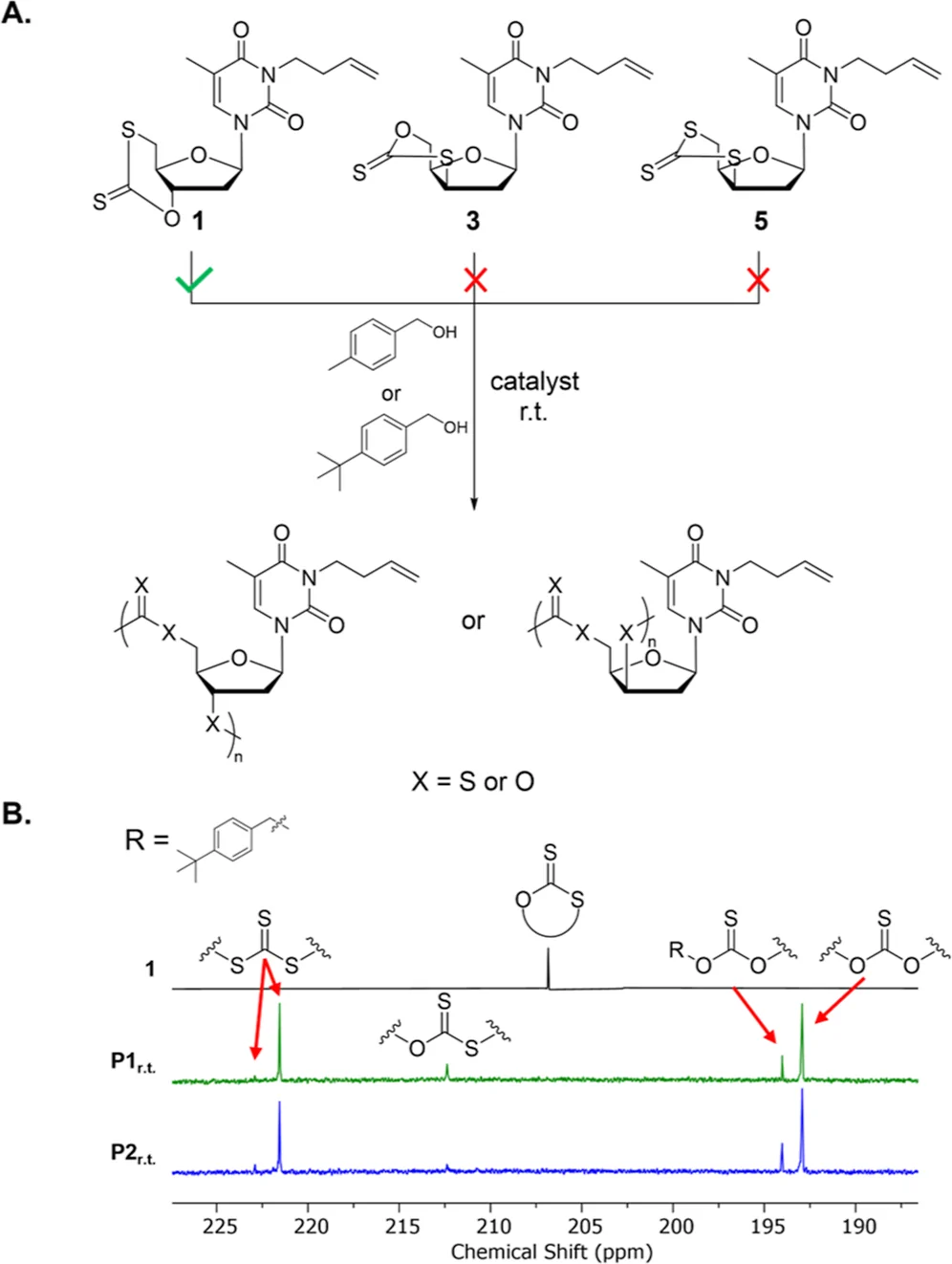
(A) ROP attempts of **1**, **3**, and **5** at ambient/room temperature (r.t.). (B) Varying thiocarbonate linkages from ROP of **1** in the presence of DBU (**P1**_**r.t.**_) and TBD (**P2**_**r.t**._) at r.t.

**Figure 3:**
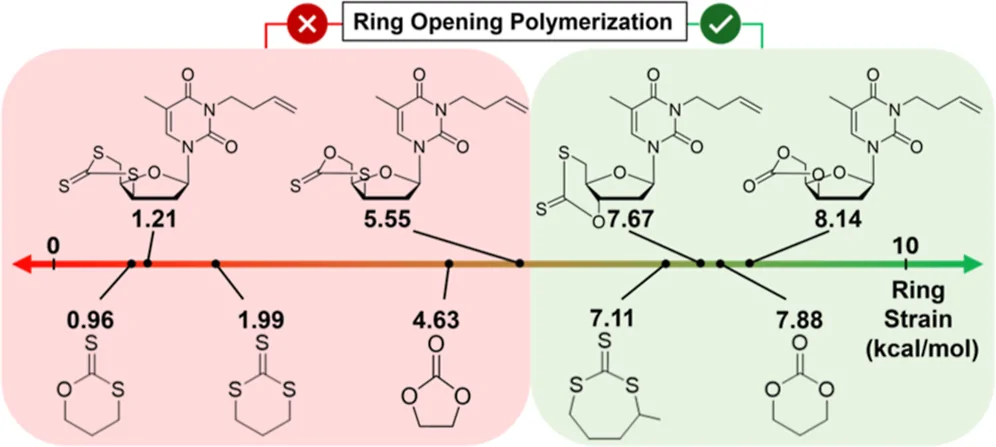
Thermodynamic roadmap evaluating the polymerizability of cyclic monomers. The axis displays the ring strain energies (enthalpies of isodesmic ring-opening reactions) in kcal/mol, calculated using DFT at the ωB97X-D/6-31++G­(d,p) level. The top row depicts the nucleoside derivatives evaluated in this study, while the bottom row shows the model cyclic compounds used to establish the thermodynamic baseline.

Initial investigations demonstrated that the ROP of **1** proceeded readily and also suggested a complex and interesting backbone microstructure. The molar ratio of monomer:initiator:catalyst was limited to 10:1:1 to afford polymers having low degrees of polymerization and easily conducive to in-depth characterization studies. Conversion of **1** in the presence of either DBU or TBD as the catalyst in dichloromethane (DCM) at 0.5 M reached >99% at r.t. after only 15 min, as determined by ^1^H NMR spectroscopy of crude polymerization mixtures. Purification was then performed by dissolution in minimal DCM followed by precipitation into methanol thrice to afford bright yellow solids with experimental number-averaged molar masses (*M*_n_) in moderate agreement with the theoretical values (Table 1, **entries 1** and **7**). The ^13^C NMR spectra of two purified polymers, **P1**_**r.t.**_ and **P2**_**r.t.**_, obtained from ROP using DBU vs TBD as catalyst, respectively, exhibited a multitude of downfield signals between 190 and 225 ppm with their relative intensities varying significantly, suggesting a range of thiocarbonate functionalities arising from cleavage of both C–O and C–S bonds during the ring-opening process (Figure 2B). Interestingly, these results contrast with a *trans*-fused 6,5-bicyclic xanthate derived from deoxyribose that yielded only xanthate linkages upon ROP in the presence of TBD at r.t.^30^ However, they are in agreement with multiple linkages being observed in the cases of the ROPs of *trans*-6,6-bicyclic and *cis*-6,5,5-tricyclic xanthate monomers derived from d-glucal^33^ and d-xylose,^30^ respectively, also reported by Buchard et al. To determine the polymer structures and their backbone connectivities, thereby providing a better understanding of the mechanisms involved during ROP, the purified polymers **P1**_**r.t.**_ and **P2**_**r.t.**_ were examined by matrix-assisted laser desorption ionization time-of-flight (MALDI-TOF) mass spectrometry and in-depth ^1^H, ^13^C, and 2D NMR spectroscopy studies (Figure 4A,B). Analyses by MALDI-TOF revealed multiple distributions with substantial populations of both linear species having the expected 4-*tert*-butylbenzyl alcohol (4-*t*-BBA)-derived chain ends and cyclic species having no chain ends (Figures S12 and S21). The significant presence of cyclic species indicates a prevalence for backbiting reactions during propagation. Further, **P1**_**r.t.**_ and **P2**_**r.t.**_ contained trithiocarbonate and thionocarbonate linkages, based on ^13^C NMR spectra including major signals at 221.6 and 192.9 ppm, respectively (Figure 2B).^46^ In agreement with work reported by Buchard and coworkers for the ROP of cyclic xanthates derived from d-glucal^33^ and d-xylose,^30^ these two prominent signals suggest a dominant alternating cleavage pathway during propagation to produce mixtures of linear and cyclic polymer structures comprised primarily of dimeric trithiocarbonate-*co*-thionocarbonate repeat units (Figure 4C,D).

**Table 1:** Results from ROP of 1 Using 4-*tert*-Butylbenzyl Alcohol as the Initiator in the Presence of DBU or TBD Organobase Catalysts at Ambient Temperature in Dichloromethane for Scaled Samples Prepared at Room Temperature and a Series of Small Scale Samples at Various Reduced Temperatures

entry	polymer^a^ ^,^ ^b^	Catalyst	*T* (°C)	time^d^	*M*_n_, Da (SEC)^f^	*D̵* (SEC)^f^	purified yield	% trithiocarbonate-*co*-thionocarbonate dimeric repeat units (*x*)^g^	% xanthate monomeric repeat units (*y*)^g^
**1**	**P1**_**r.t.**_ ^b^	DBU	r.t.	15 min	4700	1.42	69%	83	17
**2**	**P1**_**0**_ ^b^	DBU	0	30 min	5500	1.37	54%	38	62
**3**	**P1**_‑20_ ^b^	DBU	–20	30 min	5100	1.30	44%	5	95
**4**	**P1**_‑40_ ^b^	DBU	–40	1 h	4400	1.23	27%	0	>99
**5**	**P1**_‑60_ ^b^	DBU	–60	4 h	4700	1.27	16%	0	>99
**6**	**P1**_‑78_ ^c^	DBU	–78	18 h^e^	-	-	-	-	-
**7**	**P2**_**r.t.**_ ^b^	TBD	r.t.	15 min	3900	1.40	66%	91	9
**8**	**P2**_**0**_ ^b^	TBD	0	30 min	4500	1.31	61%	92	8
**9**	**P2**_‑20_ ^b^	TBD	–20	30 min	4900	1.32	62%	70	30
**10**	**P2**_‑40_ ^b^	TBD	–40	1 h	4300	1.39	47%	11	89
**11**	**P2**_‑60_ ^b^	TBD	–60	4 h	3700	1.34	19%	0	>99
**12**	**P2**_‑78_ ^c^	TBD	–78	18 h^e^	-	-	-	-	-

a The initial concentration of **1** was 0.5 M in dichloromethane.

b Molar ratio of M:I:catalyst of 10:1:1, giving a theoretical *M*_n_ = 3700 Da calculated from the monomer to initiator ratio plus chain ends.

c Molar ratio of M:I:catalyst of 20:1:1.

d Time point at which >99% conversion of **1** was reached, as monitored by comparing ^1^H NMR integration values for the resonance signals of the anomeric proton of **1** (6.06 ppm) with the anomeric proton of the resulting polymer (ca. 6.11 ppm–6.29 ppm).

e 0% conversion of **1** occurred at −78 °C, after ≥18 h.

f *M*_n_ (SEC) and *D̵* (SEC) were measured by THF SEC using polystyrene standards.

g Fraction of repeat units, as shown in the chemical structures for the polymers in Figure 4, Figures S15 and S16.

**Figure 4:**
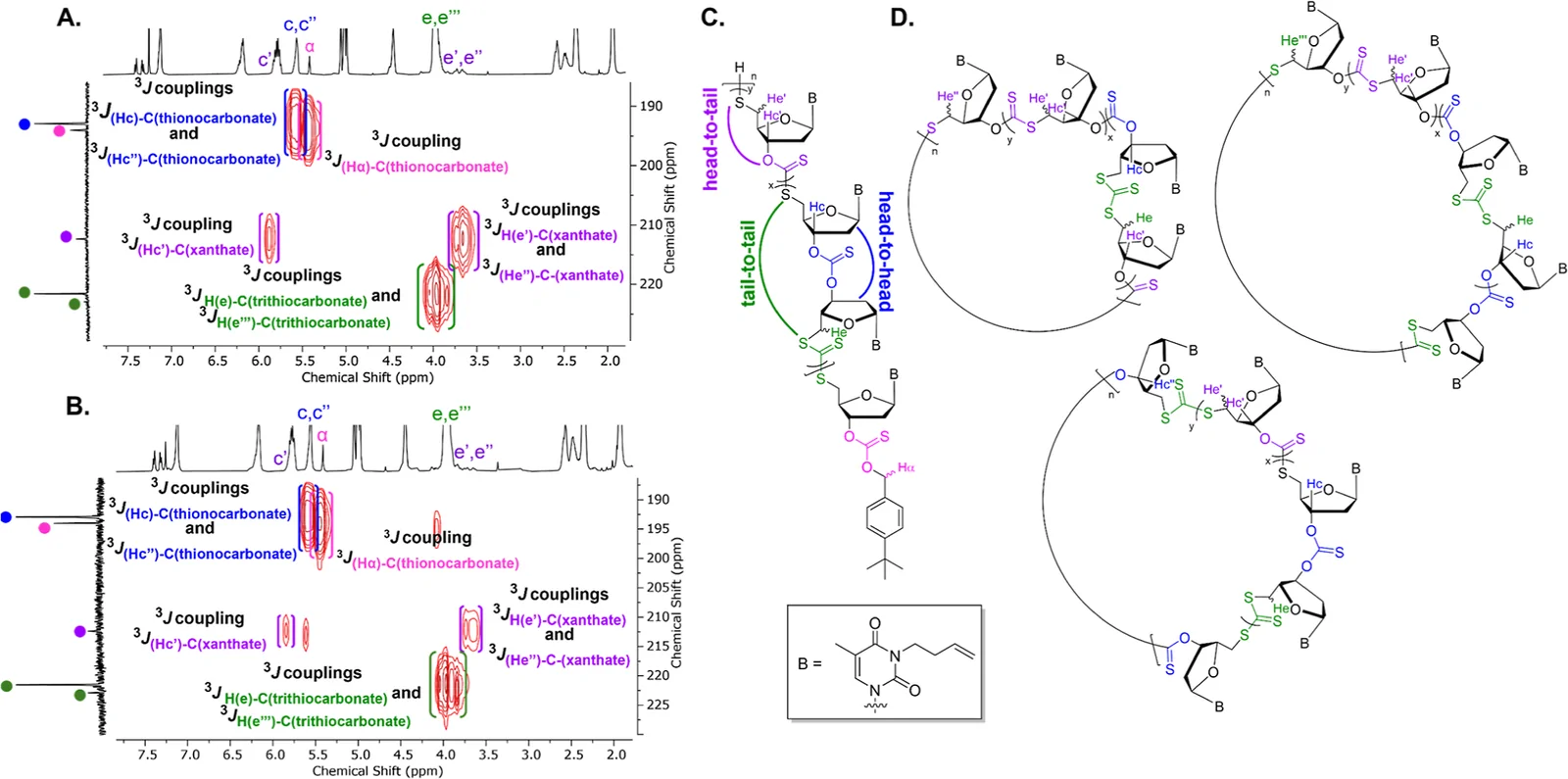
HMBC spectra of (A) **P1**_**r.t.**_ and (B) **P2**_**r.t.**_ for (C) proposed linear structure and (D) proposed cyclic structures within the polymeric mixtures.

Additionally, observation of the three minor signals at 222.9, 212.4, and 194.0 ppm, assigned to trithiocarbonate, xanthate, and thionocarbonate linkages, respectively, with intensities slightly varying based on the organobase catalyst employed, provided further information regarding the nature of the connections at the chain ends and those installed during macrocycle formation. The minor, sharp thionocarbonate signal resonating at 194.0 ppm is indicative of initiation by the alcohol-based initiator followed by cleavage of the C–S5′ bond from the tetrahedral intermediate during ring opening. Likewise, the minor xanthate could have resulted from initiation followed by cleavage of the C–O3′ bond. However, because ^1^H–^13^C heteronuclear multiple-bond (HMBC) results do not support an initiator-to-repeat unit xanthate linkage, the minor xanthate species is more likely to have arisen, along with the signal corresponding to the minor trithiocarbonate functionalities, from more complicated propagation/termination pathways. Those pathways could include minor regiorandom cleavage or cyclization via intramolecular backbiting reactions, as supported by the MALDI-TOF data. This cyclization pathway can proceed through nucleophilic attack of either the primary thiolate or secondary alkoxide onto the in-chain thiocarbonate moieties, ultimately resulting in possible trithiocarbonate, xanthate, and/or thionocarbonate connecting linkages. Due to the greater nucleophilicity of the primary thiolate compared to the secondary alkoxide, it is presumed that the majority of the cyclic structures contain either trithiocarbonate or xanthate cyclic connections. Based on these structural characterization attributes, general linear and cyclic structures within the complex polymeric mixtures are proposed as being comprised primarily of dimeric trithiocarbonate-*co*-thionocarbonate repeat units, potentially also xanthate connections between repeat units, and with thionocarbonate α-chain ends for linear chains and mixtures of minor populations of xanthate, trithiocarbonate, and thionocarbonate linkages incorporated within the macrocyclic structures (Figure 4C,D).

HMBC 2D NMR analyses were conducted to support the structural details and the connectivity of the proposed polymer structures. The HMBC spectrum for **P1**_**r.t.**_ (Figure 4A) displays a cross peak at (5.41 ppm, 194.0 ppm), resulting from the ^3^ *J*_CH_ coupling between methylene protons, H_α_, and the minor thionocarbonate thiocarbonyl carbon, supporting C–S5′ cleavage during alcohol-based initiation to yield a thionocarbonate functionality at the α-chain end. For backbone composition within either linear or cyclic polymer structures, one cross-peak at (5.56 ppm, 192.9 ppm) for a ^3^ *J*_CH_ coupling between O3′ methine protons, H_c_ and H_c’’_, and the major thionocarbonate thiocarbonyl carbon was detected, confirming head-to-head connectivities via 3′,3′-linkages for the thionocarbonate-containing repeat units (as labeled for the linear polymer structure of Figure 4C). Similarly, a cross-peak at (3.82–4.04 ppm, 221.6 ppm), arising from ^3^ *J*_CH_ coupling between the S5′ methylene protons, H_e_ and H_e’’’_, and the trithiocarbonate thiocarbonyl carbon, confirmed tail-to-tail connectivity of the trithiocarbonate repeat units via 5′,5′-linkages. Finally, two cross-peaks were observed at (5.86 ppm, 212.4 ppm) and (3.66–3.74 ppm, 212.4 ppm) for the ^3^ *J*_CH_ coupling between the O3′ methine proton, H_c’,_ with the xanthate thiocarbonyl carbon, and the ^3^ *J*_CH_ coupling between S5′ methylene protons, H_e’_ and H_e’’_, with the xanthate thiocarbonyl carbon, respectively. The combination of these cross-peaks indicates head-to-tail, 3′,5′-connectivities for the xanthate-containing repeat units. Likewise, the HMBC spectrum for **P2**_**r.t.**_ (Figure 4B) exhibited the same cross-correlations as **P1**_**r.t.**_. However, additional cross-peaks for potentially more complex polymeric structures are present.

In attempts to favor C–S5′ bond cleavage to form exclusively xanthate monomeric repeat units with head-to-tail connectivities and suppress backbiting reactions, ROPs were conducted at lower temperatures in the presence of DBU or TBD (Table 1, **Entries 2**–**6** and **8**–**12**). Polymerizations at temperatures of 0, −20, −40, and −60 °C were allowed to proceed until complete consumption of **1**, with only minimal ring opening occurring at −78 °C, and the resulting polymer linkages were then characterized by NMR and MALDI-TOF. Initial qualitative analyses of the crude polymer mixtures by ^13^C NMR spectroscopy indicated significant selectivity towards C–S5′ bond cleavage upon decreasing temperature in the presence of either catalyst, starting at −20 °C for DBU-catalyzed polymerizations and −40 °C for TBD-catalyzed polymerizations (Figure 5). Below those temperatures, nearly exclusive regioselective C–S5′ bond cleavage occurred to afford primarily xanthate linkages. After purification via precipitation into methanol, this regiochemical selectivity was further corroborated by the quantification of approximate ratios of trithiocarbonate-*co*-thionocarbonate dimeric repeat units and xanthate monomeric repeat units for the purified polymers by integration values of ^1^H NMR resonances at ca. 5.94–5.75 ppm and 5.65–5.50 ppm, which correspond to the methine protons adjacent to the thiocarbonate moieties (Table 1 and Figures S15 and S16). However, HMBC spectra for each polymer revealed ^1^H–^13^C correlations at (3.97 ppm, 221.1 ppm) and (3.89 ppm, 221.1 ppm) with varying intensities, indicating the formation of trithiocarbonate functionalities regardless of polymerization temperature (Figures S17 and S18). For polymers that do not result from alternating cleavage, these trithiocarbonate moieties are hypothesized to form from minor C–O3′ cleavage during macrocycle formation, yielding cyclic polyxanthates with trithiocarbonate connecting linkages.

**Figure 5:**
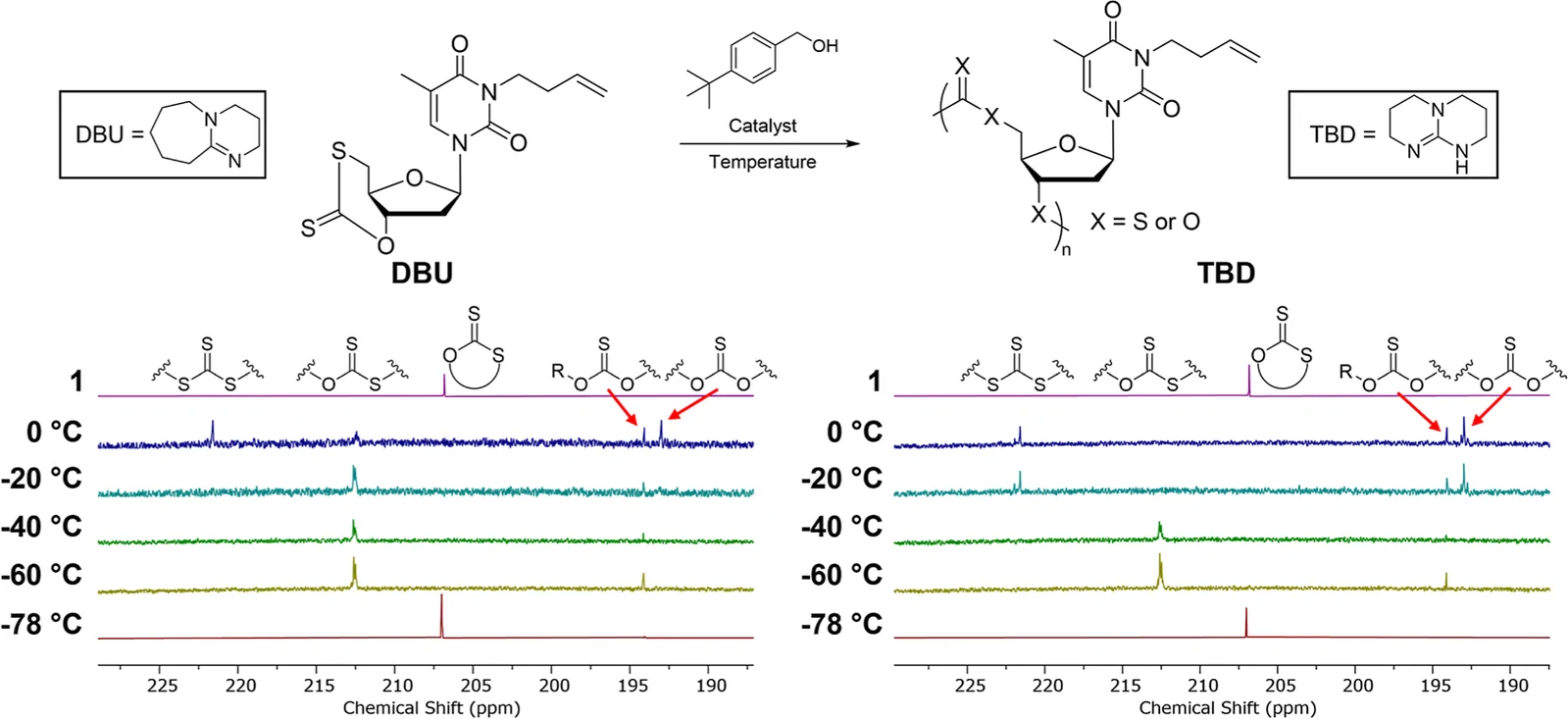
Expanded window (185 ppm–230 ppm) of crude ^13^C NMR spectra for the polymerization of **1** at varying temperatures, showing change in regiochemical cleavage in the presence of DBU or TBD.

Finally, MALDI-TOF spectra of the polymers (Table 1, **entries 2**–**5** and **8**–**11**) were obtained to determine chain ends and the relative extent of cyclization. In addition to somewhat proportional amounts of linear chains with the expected 4-*t*-BBA chain ends and cyclic species bearing no chain ends, multiple other major distributions were present in significant quantities. Although these distributions maintained the expected repeating unit, the polymer structures to account for the calculated residual masses are not yet known, suggesting limited polymerization control (Figures S19 and S22). Further, a substantial reduction in the purified yield was observed with increased regiochemical ROP cleavage control, indicating significant fractionation during purification. Therefore, MALDI-TOF spectra of the supernatants acquired from purification of samples were obtained, revealing mainly cyclic species of lower molar mass values (Figure S20). These data suggest less control over cyclization for the polymerizations conducted at lower temperatures and longer reaction times, with further studies needed to understand these factors affecting backbiting to potentially allow for promotion or mitigation of cyclization via implementation of time and temperature.

## Conclusions

The scope of this work involved the expansion of fundamental studies for the integration of sulfur chemistries in the development of monomers and polymers from naturally derived building blocks through syntheses of d-thymidine-derived, sulfur-containing cyclic monomers and investigations into their ROPs. Initial aims included the synthesis of a *trans*-6, 5-membered bicyclic xanthate derived from d-thymidine, **1**, and its ROP. Due to the same stereochemical configuration, furanose core, and bicyclic scaffold, the results of the ROP of **1** were anticipated to parallel those for the ROP of the deoxyribose-derived cyclic xanthate, in which regioselective C–S cleavage was achieved in the presence of TBD at r.t.^30^ Thus, a facile approach to generate nucleoside-derived, sulfur-containing polymers that maintain the structural integrity of native DNA was envisioned. However, applications of these sulfur-related chemistries to d-thymidine deviated from the expected outcomes, revealing interesting transformations, yet also requiring reaction optimizations for the intended monomer and polymers.

Monomer development involved assessment of two synthetic strategies, including two-step, one-pot and two-step, two-pot syntheses. Implementation of the two-step, one-pot strategy yielded **1**, but in poor yield and in addition to the *cis*-fused regioisomer **3**, as confirmed by 1D and 2D NMR spectroscopies. Interestingly, when the two-step, two-pot approach was executed, the cyclic monomers isolated and their respective yields differed substantially, depending on the base employed to facilitate CS_2_ insertion and subsequent cyclization. Utilizing DBU, a *cis*-6, 5-membered cyclic trithiocarbonate, **5**, from d-thymidine was discovered as a minor product, with the complicated mechanistic details not yet being fully understood. Conversely, CS_2_ insertion and cyclization promoted by *t*-BuOK favored cyclic xanthate formation, giving a good yield of **1**, however, with sensitive temperature and other adjustments needed to optimize reaction conditions.

Organobase-catalyzed ROPs of all isolated monomers were probed. However, due to low ring strain of **3** and **5**, as determined experimentally and computationally, only ROP of **1** proceeded and was pursued, with the resulting polymers structurally characterized by MALDI-TOF spectrometry and NMR spectroscopy techniques. Similar to our previous work evaluating the ring-opening copolymerization of a d-xylose-based oxetane with COS,^27^ polymerization temperature was identified as a tool to transform the polymeric backbone composition through altering the favored propagation cleavage pathway. Regardless of the catalyst employed, relatively increased temperatures encouraged alternating C–S5′ and C–O3′ cleavage, giving mostly dimeric 5′,5′-trithiocarbonate-*co*-3′,3′-thionocarbonate linkages with head-to-head and tail-to-tail connectivities, while lower temperatures instead favored monomeric 3′,5′-xanthate linkages with head-to-tail regiochemistries through selective C–S5′ cleavage. Therefore, structural advantages of this d-thymidine cyclic xanthate seem to arise from both its chemical complexity from the nitrogenous base, contributing to alternating C–O and C–S chemical cleavage at room temperature, and also the *trans*-fused, 6,5-membered bicyclic scaffold that possesses sufficient ring strain to undergo ROP at reduced temperatures to allow for a change in propagation pathway that involves selective C–S cleavage. Despite this ability to modulate the polymer backbones, control over chain ends and linear vs cyclic architecture remained challenging under the conditions investigated in this study, hindering the isolation of linear polymers with higher molar masses in significant yields. Therefore, ongoing work includes imparting mediation over initiation/termination/cyclization events while maintaining regiochemical control via implementation of other catalyst/initiator/solvent systems to obtain higher molar mass polymers. Then, given the range of polymeric linkages and architectures, polymer properties will be evaluated to determine structure–property-stability relationships. Further, future studies include the expansion of this methodology to other nucleoside building blocks to build a library of nucleic-acid-mimicking polythiocarbonates.

## Supplementary Material





## Notes

(ZIP)
Experimental procedures, computational details, and characterization data, including FTIR, ^1^H NMR, ^13^C NMR, HMBC, NOESY, SEC, MALDI-TOF spectra, HRMS, and other data for all compounds (PDF)
