# Supplementary material for: Investigation of Sulfur-related Influences on the Construction and Ring Opening of a Six-membered Cyclic Xanthate Derived from d‑Thymidine and CS2
Source: J Am Chem Soc. 2026 Oct 5;148(40):43812–21. doi: 10.1021/jacs.6c15290 (PMC13693707; doi:10.1021/jacs.6c15290)
Supplement: Supplementary file 1 [file ja6c15290_si_002.pdf]

Supporting Information

Investigation of Sulfur-related Influences on the Construction and Ring Opening of a Six-membered Cyclic Xanthate Derived from D-Thymidine and CS_2

Autumn M. Andras,[†] Nehal Fatima,[†] Kai-Hua Mick Kuo,[†] David K. Tran,[†] Donald J. Darensbourg,[†] and Karen L. Wooley*,[†,‡]

[†]Departments of Chemistry and [‡]Materials Science & Engineering, Texas A&M University, College Station, Texas 77843, USA

*Corresponding author e-mail: wooley@chem.tamu.edu

I. Experimental Procedures and Characterization Data

Materials 4

Instrumentation, Methods, and Analysis..4-5

Equipment 5

Synthetic protocols..5-9

II. Figures and Scheme

Figure S1. ^{1}H NMR (500 MHz, $CDCl_3$) and ^{13}C NMR (126 MHz, $CDCl_3$) spectra of 4........10

Figure S2. ^{1}H NMR (500 MHz, $CDCl_3$) and ^{13}C NMR (126 MHz, $CDCl_3$) spectra of 1........11

Figure S3. ^{1}H NMR (500 MHz, $CDCl_3$) and ^{13}C NMR (126 MHz, $CDCl_3$) spectra of 3........12

Figure S4. ^{1}H NMR (500 MHz, $CDCl_3$) and ^{13}C NMR (126 MHz, $CDCl_3$) spectra of 5........13

Figure S5. ^{1}H NMR (500 MHz, $CDCl_3$) and ^{13}C NMR (126 MHz, $CDCl_3$) spectra of $P1_{r.t.}$. .14

Figure S6. ^{1}H NMR (500 MHz, $CDCl_3$) and ^{13}C NMR (126 MHz, $CDCl_3$) spectra of $P2_{r.t.}$. .15

Figure S7. Full and expanded views of NOESY (500 MHz, $CDCl_3$) spectrum of 1..........16

Figure S8. Full and expanded views of NOESY (500 MHz, $CDCl_3$) spectrum of 3..........17

Figure S9. Full and expanded views of NOESY (500 MHz, $CDCl_3$) spectrum of 5..........18

Scheme S1. Proposed mechanism for transformation of 1 to 5....................................19

Figure S10. Full view of HMBC (^{1}H NMR 500 MHz, ^{13}C NMR 126 MHz, $CDCl_3$) spectrum of $P1_{r.t.}$..20

Figure S11. Full view of HMBC (^{1}H NMR 500 MHz, ^{13}C NMR 126 MHz, $CDCl_3$) spectrum of $P2_{r.t.}$..21

Figure S12. MALDI-TOF spectra of $P1_{r.t.}$ and $P2_{r.t.}$ and proposed linear and cyclic polymeric structures 22

Figure S13. SEC traces (THF as eluent, 1 mL/min) of P1 polymers generated from ROP of 1 in the presence of DBU at varying temperatures 23

Figure S14. SEC traces (THF as eluent, 1 mL/min) of P2 polymers generated from ROP of 1 in the presence of TBD at varying temperatures 24

Figure S15. Full and expanded views of ^{1}H NMR (500 MHz, $CDCl_3$) for all P1 polymers, prepared from 1 using DBU as the organobase catalyst..25

Figure S16. Full and expanded views of ^{1}H NMR (500 MHz, $CDCl_3$) for all P2 polymers, prepared from 1 using TBD as the organobase catalyst 26

Figure S17. Expanded views of thiocarbonate region in the HMBC spectra for P1 polymers 27

Figure S18. Expanded views of thiocarbonate region in the HMBC spectra for P2 polymers 28

Figure S19. MALDI-TOF spectra for purified P1 and P2 polymers 29

Figure S20. MALDI-TOF spectra for supernatants of purified P1 and P2 polymers 30

Figure S21. Expanded view of $P1_{r.t.}$ MALDI-TOF spectrum to demonstrate repetition of m/z values for proposed linear and cyclic species having primarily trithiocarbonate-co-thionocarbonate dimeric repeating units. 31

Figure S22. Expanded view of $P1_{-60.}$ MALDI-TOF spectrum to demonstrate repetition of m/z values for proposed linear and cyclic species 32

III. Computational Details and Related Figures, Table, and Data

Figure S23. Cyclic monomers for DFT calculations of ring strains 33

Figure S24. Consideration of ring strains by calculations of the enthalpies of isodesmic ring-opening reactions 34

Table S1. DFT calculations of the ring strain energies (kcal/mol) of different cyclic carbonates, cyclic trithiocarbonates, and cyclic xanthates 35

Appendix 1. Ensemble average of enthalpies and free energies of each conformation under ωB97X-D/6-31^{++}G(d,p) level 36-39

Appendix 2. Ensemble average of enthalpies and free energies of each conformation under M062X/6-31^{++}G(d,p) level 40-44

IV. References 45

I. Experimental Procedures and Characterization Data

Materials:

D-Thymidine, 1,8-diazabicyclo[5.4.0]undec-7-ene (DBU), 4-bromo-1-butene, and calcium hydride (CaH_2) were received from Oakwood Chemical (Estill, SC). Potassium *tert*-butoxide (*t*-BuOK*) was* used as received from Oakwood Chemical and stored in a glovebox under Ar atmosphere. 1,5,7-Triazabicyclo[4.4.0]dec-5-ene (TBD) was used as received from MedChemExpress (Monmouth Junction, NJ) and stored in a glovebox under Ar atmosphere. Pyridine (py), magnesium sulfate ($MgSO_4$), phosphorous pentoxide (P_2O_5), *p*-toluenesulfonyl chloride (TsCl), triethylamine (TEA), methanesulfonyl chloride (MsCl) and anhydrous carbon disulfide (CS_2) were received from Sigma-Aldrich (St. Louis, MO). (4-(*tert*-Butyl)phenyl)methanol (4-*t*-BBA) was received from Ambeed, Inc. (Buffalo Grove, IL). Anhydrous acetonitrile (MeCN) was used as received from Fischer Scientific (Waltham, MA) and stored in a glovebox under Ar atmosphere. Dichloromethane (DCM), *N,N*-dimethylformamide (DMF), and tetrahydrofuran (THF) were purified by a solvent purification system (J. C. Meyer Solvent Systems, Inc., Laguna Beach, CA). Py, DBU, and TEA were dried over CaH_2 and distilled prior to use. TsCl was purified by recrystallization from diethyl ether. 4-*t*-BBA was dried over molecular sieves and stored under Ar atmosphere. Compounds **1**, **3**, and **5** were dried under reduced pressure with P_2O_5 for 3 d and stored in the glovebox under Ar atmosphere before polymerization. All reactions were performed under an Ar or N_2 atmosphere, using glovebox or Schlenk line techniques. Glassware was flame-dried or oven-dried at 110 °C for 24 hours prior to use.

Instrumentation, Methods, and Analysis:

^1H NMR, ^{13}C NMR, (^1H-^1H) COSY, (^{13}C-^1H) HSQC (^{13}C-^1H) HMBC, and (^1H-^1H) NOESY spectra were recorded on an Avance 500 with an Oxford magnet, an automated tuning 5 mm ^1H/^{13}C/^{15}N cold probe, and a 24 position SampleCase sample changer interfaced to a UNIX computer using the VnmrJ software. Chemical shifts for ^1H NMR and ^{13}C NMR signals were referenced to the solvent resonance frequencies. The integration values for the ^1H NMR data are reported as the relative values based upon protons present within each repeat unit rather than the total protons per polymer chain, as there are significant differences in repeat unit compositions and structures as a function of the temperature under which the polymerizations were performed, in addition to different chain ends resulting from the presence of initiator *vs* backbiting reactions leading to cyclic species. The approximate ratio between xanthate monomeric repeat units to trithiocarbonate-*co*-thionocarbonate dimeric repeat units was calculated as follows:

$$\frac{\text{xanthate monomeric repeat unit}}{\text{trithiocarbonate} - co - \text{thionocarbonate dimeric repeat unit}} = \frac{(\mathrm{Hj} + \mathrm{Hc}'') - 1}{0.5 \times ((\mathrm{Hc} + \mathrm{Hc}') - (0.5 \times \mathrm{H\alpha}))}$$

Of note, subtraction of 1 from the (Hj + Hc'') term is to approximate the relative value of Hc''.

Fourier transform-infrared (FT-IR) spectra were recorded on either an IR Prestige 21 system, equipped with a diamond attenuated total reflection (ATR) lens (Shimadzu Corp., Japan) or a Bruker LUMOS-II micro-FTIR system equipped with a germanium ATR lens (Bruker Corp., Billerica, MA). Samples recorded on the Bruker LUMOS-II micro-FTIR system were first flattened using a diamond compression cell. Data obtained were analyzed using IRsolution *v.* 1.40 or OPUS software.

Size exclusion chromatography (SEC) eluting with THF was conducted on a Waters Corporation, Inc. (Milford, MA) system equipped with an isocratic pump model 1515, a differential refractometer model 2414, and three Styragel columns (PLgel 5 µm Mixed C, 500 Å, and 104 Å, 300 × 7.5 mm columns). The system was operated at 40 °C with a flow rate of 1 mL/min. Data

were analyzed using Breeze *v*. 6.20 software from Waters Chromatography, Inc. (Milford, MA). Molar masses were determined relative to polystyrene standards (300–467,000 Da) purchased from Polymer Laboratories, Inc. (Amherst, MA). Polymer solutions were prepared at a concentration of *ca*. 3 mg/mL with 0.05 vol% toluene as flow rate marker and an injection volume of 200 μL was used.

Electrospray ionization mass spectrometry (ESI-MS) experiments were performed using a Thermo Scientific Q Exactive Focus. Sample was injected into a 10 μL loop and was transferred to the mass spec using a mobile phase containing 70% methanol and 30% water with 0.1% formic acid at a flow rate of 600 μL/min. The Q Exactive Focus HESI source was operated in full MS in positive mode and negative mode. The mass resolution was tuned to 70000 FWHM at m/z 200. The spray voltage was set to 3.5 kV for positive mode and 2.8 kV for negative mode. The sheath gas and auxiliary gas flow rates were set to 40 and 10 arbitrary units, respectively. The vaporizer and transfer capillary temperatures were held at 250 and 320 °C, respectively. The S-Lens RF level was set at 50 v. Exactive Series 2.11/Xcalibur 4.2.47 software was used for data acquisition and processing. Matrix-assisted laser desorption ionization (MALDI) experiments were performed using a Bruker Neoflex MALDI-TOF mass spectrometer (Bruker Daltonics) operated in optimized conditions in positive linear mode. About 1 mg of the sample was dissolved in 200 μL of tetrahydrofuran. *trans*-2-[3-(4-*tert*-Butyl-phenyl)-2-methyl-2-propenylidene] malononitrile (DCTB) (10 mg/mL) and potassium trifluoroacetic acid (KTFA) (10 mg/ml) were used as a matrix and cationization agent, respectively. One microliter of sample, 1 μL of KTFA, and 20 μL of DCTB were mixed thoroughly. About 0.5 μl of this mixture was deposited on a stainless-steel sample holder. After air-drying, the sample was analyzed using MALDI-TOF MS.

Equipment:

Automated chromatography was performed with Teledyne Isco CombiFlash NextGen 300+ Automated Flash Chromatography System with 20-40 μm particle size silica gel disposable flash columns.

Reactions proceeding at 0 °C or below were performed using EYELA PSL-1810 Constant Temperature Bath with Magnetic Stirrer unless stated otherwise.

Polymers were isolated after precipitation using a Hermle Z36-HK centrifuge.

Synthetic Protocols

Synthesis of *N*^{3}-(3-butenyl) thymidine (2):

Following an adapted procedure:[1] To a 100-mL round bottom flask equipped with a magnetic stir bar was added D-thymidine (5.01 g, 20.7 mmol) and potassium carbonate (5.71 g, 41.3 mmol) followed by DMF (27.0 mL) to create a suspension. To the stirred suspension was added 4-bromo-1-butene (3.80 mL, 37.4 mmol). The reaction mixture was heated to 60 °C and allowed to stir for 24 h before it was cooled back to room temperature and concentrated *in vacuo*. The residue was dissolved in ethyl acetate and washed with water and brine. The organic layers were combined, dried over magnesium sulfate, filtered, and concentrated *in vacuo*. The crude product was purified by silica gel automated column chromatography with ethyl acetate as the eluent to give **2** as a white solid (4.96 g, 16.7 mmol, 81% yield).

The spectroscopic data for this compound were identical to those reported in literature.[1]

Synthesis of *N*^{3}-(3-butenyl)-5'-tosyl-D-thymidine (4):

To a 100-mL round bottom flask equipped with a stir bar was added **2** (3.01 g, 10.2 mmol) and anhydrous py (24.0 mL). The reaction flask was then placed in an ice-water bath, and tosyl chloride (2.13 g, 11.2 mmol) was added to the reaction flask in batches. The ice-water bath was

removed to allow the reaction flask to gradually warm to room temperature and the mixture was stirred for 48 hours under N_2 atmosphere. The reaction was quenched by the addition of methanol and the solvents were evaporated *in vacuo*. The resulting residue was diluted with 5% aq. HCl and extracted with EtOAc thrice, followed by a brine wash. The organic layers were combined, dried over magnesium sulfate, filtered, and concentrated *in vacuo*. The product was then purified by silica gel gravity column chromatography (ethyl acetate/hexanes = 1:1) followed by recrystallization from ethyl acetate in hexanes to give the product as a white solid (1.56 g, 3.46 mmol, 34% yield). R_f = 0.25. ^{1}H NMR (500 MHz, $CDCl_3$) δ 7.80 – 7.78 (m, 2H), 7.37 (d, *J* = 8.1 Hz, 2H), 7.34 (d, *J* = 1.4 Hz, 1H), 6.35 (t, *J* = 6.7 Hz, 1H), 5.80 (ddt, *J* = 17.1, 10.2, 6.9 Hz, 1H), 5.05 (dq, *J* = 17.1, 1.6 Hz, 1H), 5.00 (ddt, *J* = 10.2, 2.1, 1.1 Hz, 1H), 4.52 (dt, *J* = 7.0, 3.7 Hz, 1H), 4.27 (d, *J* = 2.8 Hz, 2H), 4.08 (q, *J* = 3.0 Hz, 1H), 3.99 (m, 2H), 2.46 (s, 3H), 2.37 (m, 3H), 2.19 (dt, *J* = 13.8, 6.8 Hz, 1H), 1.93 (d, *J* = 1.3 Hz, 3H). ^{13}C NMR (126 MHz, $CDCl_3$, ppm) δ 163.4, 151.0, 145.8, 135.0, 133.4, 132.5, 130.3, 128.0, 117.0, 110.9, 85.6, 83.8, 71.4, 68.9, 40.8, 40.5, 32.01, 21.8, 13.3. FT-IR (ATR): 3489 – 3325, 3120 – 3032, 3011 – 2878, 1697, 1658, 1612, 1465, 1350, 1273, 1172, 1080, 1041, 1002, 972, 902, 817, 771, 725, 663, 547. HRMS (ESI$^+$) Calcd [M+H$^+$] for $C_{21}H_{26}N_2O_7S_1H^+$ 451.1533, found: 451.1531

Synthesis of 3',5'-Cyclic *N*³-(3-butenyl)-D-thymidine *trans* xanthate (1) and 3',5'-Cyclic *N*³-(3-butenyl)-D-thymidine *cis* xanthate (3):

Following an adapted procedure:[2] To a 25-mL round bottom flask equipped with a stir bar was added **2** (2.01 g, 6.78 mmol) followed by anhydrous MeCN (13.5 mL). Anhydrous CS_2 (1.63 mL, 27.1 mmol) was added to the reaction flask and allowed to stir at room temperature for approximately 5 minutes. DBU (1.01 mL, 6.77 mmol) was then added to the flask, and the reaction was stirred for 1 h. The flask was placed in an ice-water bath and TEA (0.94 mL, 6.7 mmol) was added to the reaction mixture followed by MsCl (0.52 mL, 6.7 mmol). The flask was removed from the ice-water bath, allowed to gradually warm to room temperature, and stirred for 12.5 h. The reaction was quenched with excess acetic acid, and the crude mixture was concentrated *in vacuo*. The products were purified by silica gel gravity column chromatography (4% acetone in chloroform) to give a pale-yellow solid, **1** (71.9 mg, 0.203 mmol, 3.0% yield), and a yellow solid, **3** (66.3 mg, 0.187 mmol, 2.8% yield). **1**. R_f = 0.58. ^{1}H NMR (500 MHz, $CDCl_3$) δ 6.95 (d, *J* = 1.5 Hz, 1H), 6.06 (dd, *J* = 8.4, 3.5 Hz, 1H), 5.80 (ddt, *J* = 17.2, 10.2, 7.0 Hz, 1H), 5.08 (dq, *J* = 17.1, 1.6, 1H), 5.01 (ddt, *J* = 10.2, 2.1, 1.1 Hz, 1H), 4.94 (q, *J* = 8.8 Hz, 1H), 4.16 (ddd, *J* = 11.1, 8.7, 5.1 Hz, 1H), 4.00 (td, *J* = 7.1, 3.2 Hz, 2H), 3.45 (dd, *J* = 11.2, 9.9 Hz, 1H), 3.27 (dd, *J* = 9.9, 5.1 Hz, 1H), 2.82 – 2.67 (m, 2H), 2.38 (qt, *J* = 7.2, 1.4 Hz, 2H), 1.95 (d, *J* = 1.3 Hz, 3H). ^{13}C NMR (126 MHz, $CDCl_3$) δ 206.8, 163.1, 150.4, 134.8, 134.6, 117.2, 111.2, 87.6, 82.6, 73.2, 40.8, 36.5, 35.4, 32.0, 13.4. FT-IR (ATR): 3002 – 2900, 2900 – 2820, 1701, 1666, 1636, 1462, 1358, 1312, 1273, 1234, 1180, 1042, 964, 914, 814, 768, 633, 610, 552. HRMS (ESI$^+$) Calcd [M+H$^+$] for $C_{15}H_{18}N_2O_4S_2H^+$ 355.0781, found: 355.0777. **3**. R_f = 0.36. ^{1}H NMR (500 MHz, $CDCl_3$) δ 7.25 (d, *J* = 1.5 Hz, 1H), 6.23 (t, *J* = 6.4 Hz, 1H), 5.80 (ddt, *J* = 17.1, 10.2, 7.0 Hz, 1H), 5.06 (dq, *J* = 17.1, 1.6 Hz, 1H), 5.01 (dd, *J* = 10.2, 1.8 Hz, 1H), 4.91 (dd, *J* = 12.7, 2.6 Hz, 1H), 4.67 (dt, *J* = 8.0, 2.6, Hz, 1H), 4.45 (dd, *J* = 12.7, 1.5 Hz, 1H), 4.06 – 3.89 (m, 2H), 3.01 (ddd, *J* = 14.7, 8.1, 6.7 Hz, 1H), 2.37 (q, *J* = 7.2 Hz, 2H), 2.19 (dt, *J* = 14.6, 5.7 Hz, 1H), 1.93 (d, *J* = 1.3 Hz, 3H). ^{13}C NMR (126 MHz, $CDCl_3$) δ 207.4, 163.1, 151.0, 134.9, 132.2, 117.1, 111.4, 84.4, 73.3, 72.8, 44.1, 41.1, 40.8, 32.1, 13.5. FT-IR (ATR): 3588, 3504, 3327, 3077, 3006, 2961, 2924, 2852, 1705, 1664, 1632, 1519, 1463, 1400, 1348, 1311, 1276, 1253, 1207, 1187, 1111, 1091, 1054, 1014, 915, 863. HRMS (ESI$^+$) Calcd [M+H$^+$] for $C_{15}H_{18}N_2O_4S_2H^+$ 355.0781, found: 355.0769.

Synthesis of 3',5'-Cyclic N^3-(3-butenyl)-D-thymidine *cis* trithiocarbonate (5):
To a 50-mL round bottom flask equipped with a stir bar were added **4** (1.00 g, 2.22 mmol) and anhydrous MeCN (22.0 mL) in a glovebox with argon atmosphere. The reaction flask was transferred into a fume hood and placed under N_2 atmosphere. DBU (0.40 mL, 2.7 mmol) was added to the reaction flask followed by anhydrous CS_2 (0.16 mL, 2.7 mmol). The reaction was allowed to stir at room temperature for 1 h. The crude reaction mixture was concentrated *in vacuo* and immediately subjected to purification by silica gel automated column chromatography using a 0-10% MeCN in $CHCl_3$ eluent system to isolate **5** in trace amounts (~10 mg, 1.2% yield) as a yellow solid. R_f = 0.83. ^1H NMR (500 MHz, $CDCl_3$) δ 7.34 (d, *J* = 1.6 Hz, 1H), 6.24 (dd, *J* = 8.6, 5.5 Hz, 1H), 5.80 (ddt, *J* = 17.1, 10.1, 7.0 Hz, 1H), 5.06 (dq, *J* = 17.0, 1.7 Hz, 2H), 5.01 (dd, *J* = 10.0, 1.9 Hz, 1H), 4.93 (dt, *J* = 8.4, 4.1 Hz, 1H), 4.12 – 3.97 (m, 3H), 3.48 (dd, *J* = 14.2, 3.5 Hz, 1H), 3.39 (dd, *J* = 14.2, 4.7 Hz, 1H), 2.84 (ddd, *J* = 13.9, 8.5, 5.5 Hz, 1H), 2.38 (q, *J* = 7.3 Hz, 2H), 2.05 (dt, *J* = 13.7, 8.8 Hz, 1H), 1.96 (d, *J* = 1.3 Hz, 3H). ^{13}C NMR (126 MHz, $CDCl_3$, ppm) δ 223.2, 163.1, 151.0, 134.9, 132.4, 117.1, 111.4, 83.2, 74.1, 49.1, 41.4, 40.9, 38.0, 32.1, 13.6. FT-IR (ATR): 3758 – 3202, 3078, 2924, 2855, 1636, 1458, 1358, 1273, 1180, 1049, 895, 764, 733, 648, 556, 401. HRMS (ESI$^+$) Calcd [M+H$^+$] for $C_{15}H_{18}N_2O_3S_3H^+$ 371.0552, found: 371.0544.

Synthesis of 3'5'-Cyclic 3-(3-butenyl)-D-thymidine *trans* xanthate (1):
To a 25-mL round bottom flask equipped with a stir bar was added **4** (2.00 g, 4.44 mmol) and anhydrous THF (4.50 mL) in a glovebox with argon atmosphere. The reaction flask was removed from the glovebox and transferred into a fume hood, placed under N_2 atmosphere, and cooled in an ethanol bath at -78 °C. A solution of *t*-BuOK (0.498 g, 4.44 mmol) in anhydrous THF (4.40 mL) was added to the reaction flask and allowed to stir at -78 °C for 2 h. Anhydrous CS_2 (0.53 mL, 8.8 mmol) was then added to the reaction mixture, and the flask was subsequently warmed to 20 °C and stirred for 3 hours. The reaction was quenched with acetic acid, diluted with DCM, and vacuum filtered through a Celite pad. The filtrate was collected and purified via silica gel gravity column chromatography using a 3.5% acetone in chloroform eluent to yield a pale yellow solid (0.914 g, 2.58 mmol, 58% yield). R_f = 0.53. **See characterization data above**.

Synthetic protocol for the organobase-catalyzed ring-opening polymerization of 1 at room temperature:
In a glovebox under argon atmosphere, **1** (100 mg, 0.282 mmol, 10 eq) was weighed into a 4-mL vial charged with a stir bar and subsequently dissolved in 0.36 mL of anhydrous DCM. A solution of 4-*t*-BBA (1 eq) in 0.1 mL anhydrous DCM was added and stirred for *ca*. 2 min, followed by a solution of organobase (1 eq) in 0.1 mL anhydrous DCM. The polymerization was allowed to proceed for 15 minutes, transferred outside of the glovebox, and quenched with acetic acid. The polymers were purified by precipitation from DCM into methanol thrice, followed by drying *in vacuo* to afford bright yellow solids.

$P1_{r.t.}$: ^1H NMR (500 MHz, $CDCl_3$) 7.40 – 7.39 (br d, *J* = 8.2 Hz), 7.33 - 7.31 (br d, *J* = 8.0 Hz), 7.14 – 7.12 (m), 6.23 – 6.15 (m), 5.87 – 5.74 (m), 5.60 – 5.55 (m), 5.41 (br s), 5.05 – 4.98 (m), 4.50 – 4.43 (m), 4.00 – 3.89 (m), 3.75 – 3.62 (m), 2.60 – 2.56 (m), 2.51 – 2.41 (m), 2.38 – 2.33 (m), 1.94 – 1.92 (m), 1.30 (br s). ^{13}C NMR (126 MHz, $CDCl_3$) δ 222.9, 221.6, 212.4,194.0, 192.9, 163.0, 152.3, 150.6, 134.9, 133.4, 130.9,128.7,128.7, 125.8,117.110.8, 86.6, 84.1, 81.2, 75.5, 40.7, 39.1, 36.9, 34.7, 32.0, 31.4, 29.8, 13.5. FT-IR (ATR): 3078, 3049 – 2817, 2322, 1636, 1458, 1358, 1265, 1219, 1188, 1057, 910, 802, 764, 648 cm^{-1}. (72.3 mg, 69% yield).

P2$_{r.t.}$: ^{1}H NMR (500 MHz, $CDCl_3$) δ 7.40 - 7.39 (br d, *J* = 8.1 Hz), 7.32 - 7.31 (br d, *J* = 8.0 Hz), 7.13 (m), 6.22 – 6.15 (m), 5.84 – 5.74 (m), 5.59 – 5.55 (m), 5.41 (br s), 5.05 – 4.98 (m), 4.49 – 4.43 (m), 3.99 – 3.89 (m),3.76 – 3.63 (m), 2.60 – 2.56 (m), 2.51 – 2.45 (m), 2.38 – 2.33 (m), 1.93 - 1.92 (m), 1.30 (br s). ^{13}C NMR (126 MHz, $CDCl_3$) δ 223.0 221.5, 212.4, 194.0, 192.9, 163.2, 163.0, 152.3, 150.6, 134.9, 133.4, 133.13, 130.9, 128.7, 125.8, 117.1, 110.8, 86.6, 84.1, 81.2, 75.5, 40.7, 39.1, 36.9, 34.8, 32.0, 31.4, 29.8, 13.5. FT-IR (ATR): 3078, 3024 – 2814 1697, 1636, 1458, 1358, 1273, 1227, 1188, 1065, 957, 910, 802, 764, 648 cm^{-1}. (68.7 mg, 66% yield).

Synthetic protocol for organobase-catalyzed ring-opening polymerization of 1 at varying temperatures:

In a glovebox under argon atmosphere, **1** (25 mg, 0.070 mmol, 10 eq) was weighed into a vial charged with a stir bar. A solution of 4-*t*-BBA in 0.1 mL (1 eq) anhydrous DCM was added to the polymerization vial and transferred outside of the glovebox to a fume hood. The polymerization vial was put under N_2 atmosphere and cooled in an ethanol bath set to a predetermined temperature for several minutes. An organobase solution was cooled in the same ethanol bath for several minutes before 40 µL (1 eq) was added to the polymerization vial via microsyringe. Once **1** was fully consumed, the polymerization was quenched with excess acetic acid.

P1$_0$: The polymerization was conducted at 0 °C in the presence of DBU for 30 min and purified via precipitation into methanol twice to obtain a yellow solid (14.2 mg, 54% yield).
^{1}H NMR (500 MHz, $CDCl_3$) δ 7.41 (br d, *J* = 8.2 Hz), 7.33 (br d, *J* = 8.1 Hz), 7.19 – 7.09 (m), 6.29 – 6.15 (m), 5.96 – 5.71 (m), 5.65 – 5.53 (m), 5.44 – 5.41 (m), 5.08 – 4.98 (m), 4.54 – 4.41 (m), 4.05 – 3.87 (m), 3.79 – 3.61 (m), 2.64 – 2.54 (m), 2.54 – 2.41 (m), 2.40 – 2.30 (m), 1.99 – 1.90 (m), 1.64 (br s), 1.31 (s).

P1$_{-20}$: The polymerization was conducted in the presence of DBU at -20 °C for 30 min and purified via precipitation into methanol twice to obtain a pale yellow solid (11.6 mg, 44% yield).
^{1}H NMR (500 MHz, $CDCl_3$) δ 7.41 (br d, *J* = 8.3 Hz), 7.34 (br d, *J* = 8.3 Hz), 7.17 – 7.10 (m, 2H), 6.29 – 6.16 (m), 5.94 – 5.73 (m), 5.65 – 5.55 (m), 5.45 – 5.41 (m), 5.08 – 4.97 (m), 4.54 – 4.43 (m), 4.06 – 3.87 (m), 3.80 – 3.62 (m), 2.65 – 2.54 (m), 2.53 - 2.41 (m), 2.40 – 2.31 (m), 1.99 – 1.92 (m), 1.61 (br s), 1.32 (s).

P1$_{-40}$: The polymerization was conducted at -40 °C in the presence of DBU for 1 h and purified via precipitation into methanol twice to obtain a pale yellow solid (7.1 mg, 27% yield).
^{1}H NMR (500 MHz, $CDCl_3$) δ 7.41 (br d, *J* = 8.3 Hz), 7.34 (br d, *J* = 8.3 Hz), 7.17 – 7.10 (m), 6.29 – 6.18 (m), 5.97 – 5.73 (m), 5.65 – 5.60 (m), 5.43 (d, *J* = 2.9 Hz), 5.09 – 4.98 (m), 4.54 – 4.43 (m), 4.06 – 3.89 (m), 3.80 – 3.61 (m), 2.65 – 2.54 (m), 2.51 - 2.41 (m), 2.41 – 2.32 (m), 1.99 – 1.92 (m), 1.60 (br s), 1.32 (s).

P1$_{-60}$: The polymerization was conducted at -60 °C in the presence of DBU for 4 h and purified via precipitation into methanol thrice to obtain a pale yellow solid (4.2 mg, 16% yield).
^{1}H NMR (500 MHz, $CDCl_3$) δ 7.41 (br d, *J* = 8.3 Hz), 7.34 (br d, *J* = 8.3 Hz), 7.17 – 7.09 (m), 6.29 – 6.19 (m), 5.93 – 5.73 (m), 5.65 – 5.60 (m), 5.43 (d, *J* = 2.9 Hz), 5.09 – 4.98 (m), 4.54 – 4.43 (m), 4.06 – 3.92 (m), 3.80 – 3.62 (m), 2.64 - 2.54 (m), 2.52 – 2.41 (m), 2.41 – 2.32 (m), 2.00 – 1.92 (m), 1.62 (br s), 1.32 (s).

$P2_0$: The polymerization was conducted at 0 °C in the presence of TBD for 30 min and purified via precipitation into methanol twice to obtain a yellow solid (16.0 mg, 61% yield).
^{1}H NMR (500 MHz, $CDCl_3$) δ 7.41 (br d, *J* = 8.3 Hz), 7.33 (br d, *J* = 8.2 Hz), 7.16 – 7.11 (m), 6.27 – 6.15 (m), 5.86 – 5.73 (m), 5.63 – 5.53 (m), 5.44 – 5.41 (m), 5.09 – 4.97 (m), 4.53 – 4.42 (m) 4.07 – 3.78 (m), 3.78 – 3.61 (m), 2.65 – 2.55 (m), 2.53 – 2.42 (m), 2.41 – 2.31 (m), 1.97 – 1.91(m), 1.63 (br s), 1.32 (s).

$P2_{-20}$: The polymerization was conducted at -20 °C in the presence of TBD for 30 min and purified via precipitation into methanol twice to obtain a yellow solid (16.1 mg, 62% yield).
^{1}H NMR (500 MHz, $CDCl_3$) δ 7.41 (br d, *J* = 8.2 Hz), 7.34 (br d, *J* = 8.1 Hz), 7.17 – 7.10 (m), 6.29 – 6.14 (m), 5.89 – 5.73 (m), 5.64 – 5.54 (m), 5.44 – 5.41 (m), 5.09 – 4.98 (m), 4.54 – 4.42 (m), 4.04 – 3.82 (m), 3.79 – 3.62 (m), 2.65 – 2.55 (m), 2.54 – 2.41 (m), 2.41 – 2.30 (m), 2.00 - 1.89 (m), 1.62 (br s), 1.32 (s.)

$P2_{-40}$: The polymerization was conducted at -40 °C in the presence of TBD for 1 h and purified via precipitation into methanol twice to obtain a pale yellow solid (12.4 mg, 47% yield).
^{1}H NMR (500 MHz, $CDCl_3$) δ 7.41 (br d, *J* = 8.3 Hz), 7.34 (br d, *J* = 8.3 Hz), 7.18 – 7.09 (m), 6.29 – 6.17 (m, 1H), 6.26 – 6.18 (m, 1H), 5.93 – 5.72 (m), 5.66 – 5.54 (m), 5.43 (d, *J* = 2.8 Hz), 5.09 – 4.96 (m), 4.55 – 4.43 (m), 4.05 – 3.88 (m), 3.80 – 3.62 (m), 2.64 – 2.54 (m), 2.53 – 2.41 (m), 2.41 – 2.32 (m), 2.00 – 1.91 (m), 1.61 (br s), 1.32 (s).

$P1_{-60}$: The polymerization was conducted at -60 °C in the presence of TBD for 4 h and purified via precipitation into methanol twice to obtain a pale yellow solid (5.1 mg, 19% yield).
^{1}H NMR (500 MHz, $CDCl_3$) δ 7.42 (br d, *J* = 8.3 Hz), 7.34 (br d, *J* = 8.3 Hz), 7.18 – 7.09 (m), 6.29 – 6.19 (m), 5.94 – 5.73 (m), 5.65 – 5.57 (m), 5.44 (d, *J* = 2.9 Hz), 5.09 – 4.98 (m), 4.54 – 4.44 (m), 4.06 – 3.89 (m), 3.82 – 3.56 (m, 2H), 2.65 – 2.54 (m, 1H), 2.54 – 2.41 (m), 2.41 – 2.32 (m), 2.01 – 1.92 (m), 1.58 (br s), 1.32 (s).

II. Figures and Scheme

500 MHz – $CDCl_3$

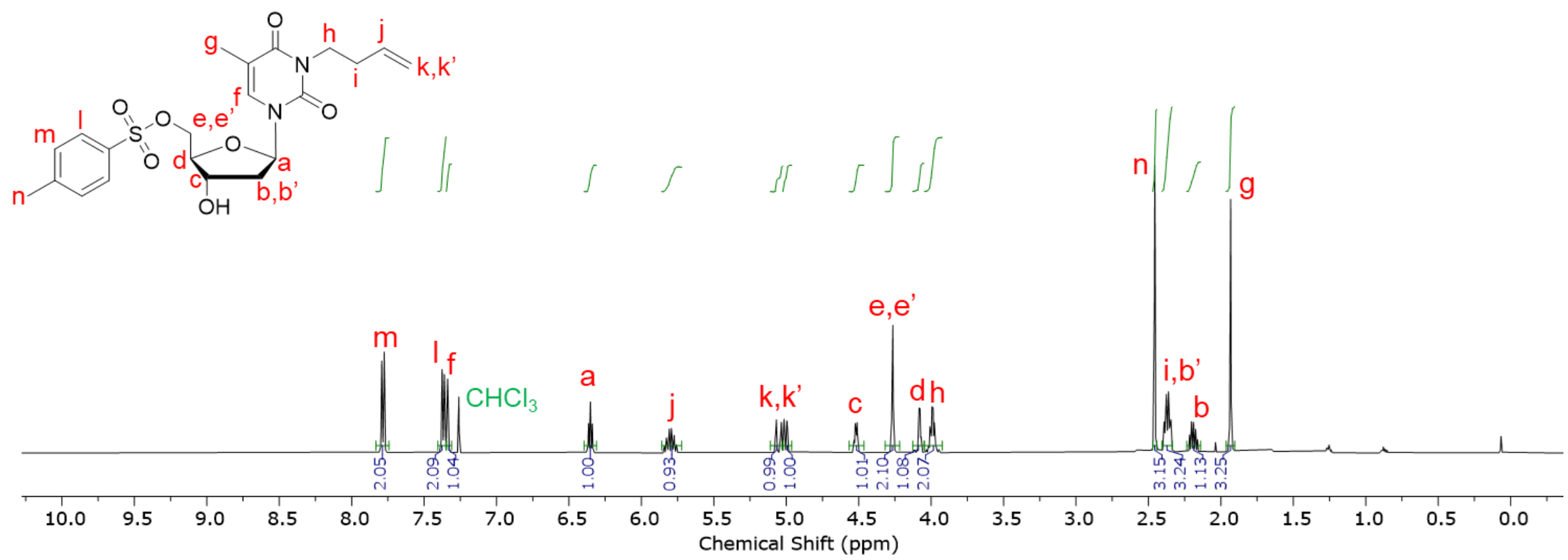

126 MHz – $CDCl_3$

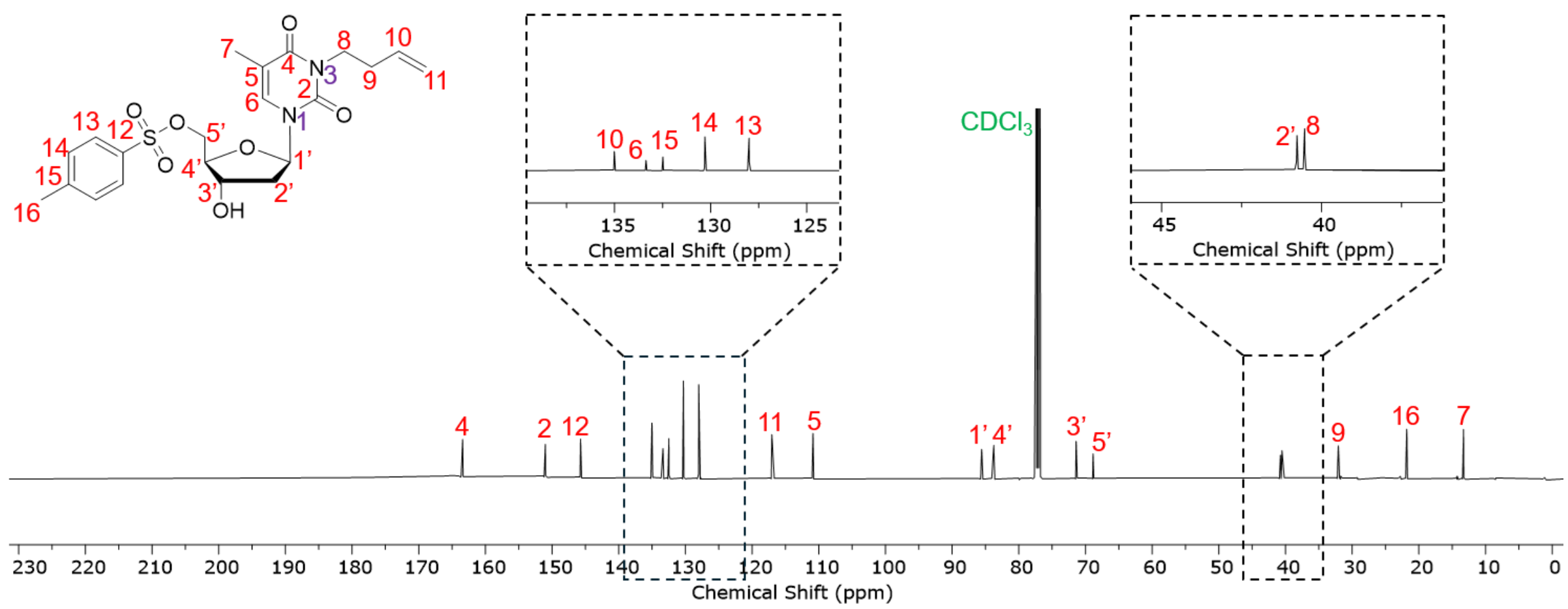

Figure S1. ^{1}H NMR (500 MHz, $CDCl_3$) and ^{13}C NMR (126 MHz, $CDCl_3$) spectra of **4**.

500 MHz – CDCl$_3$

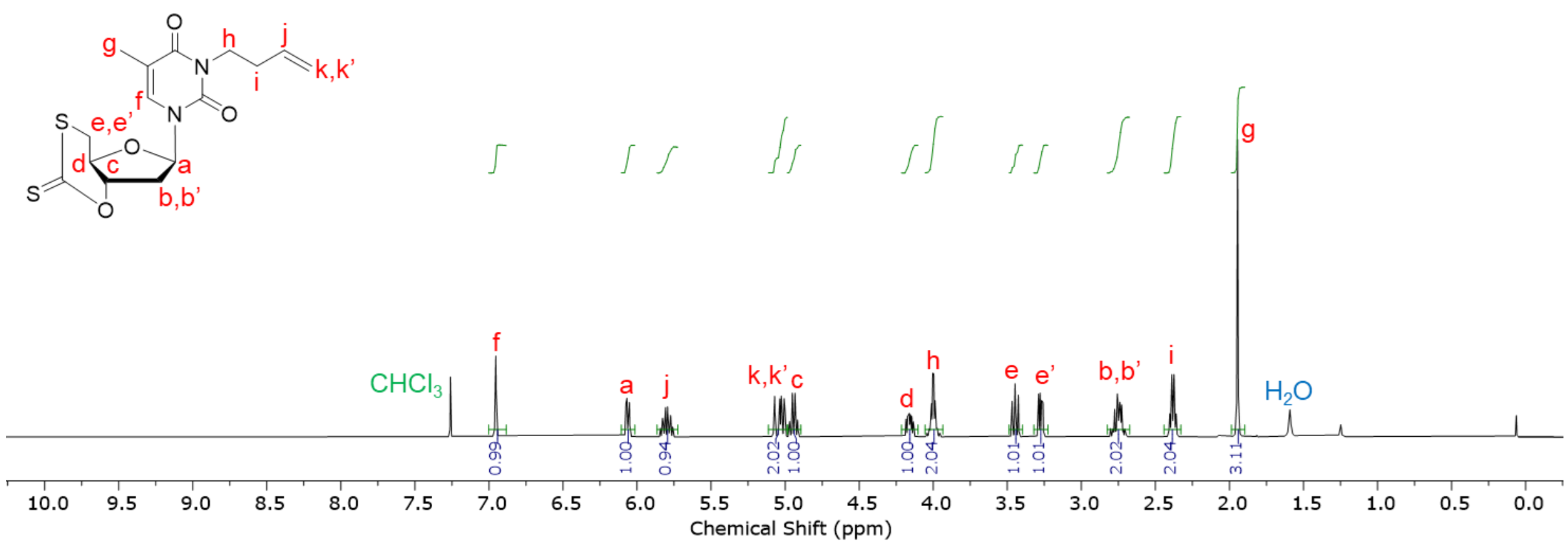

126 MHz – CDCl$_3$

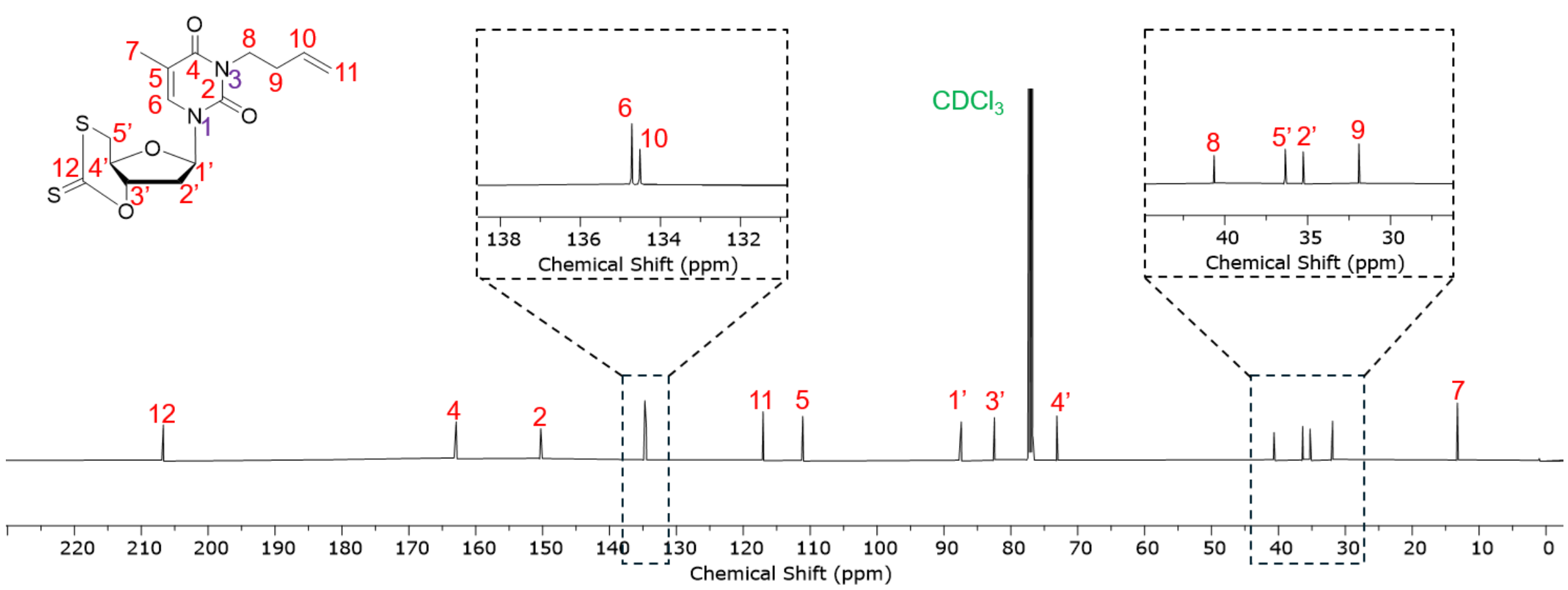

Figure S2. ^{1}H NMR (500 MHz, CDCl$_3$) and ^{13}C NMR (126 MHz, CDCl$_3$) spectra of **1**.

500 MHz – $CDCl_3$

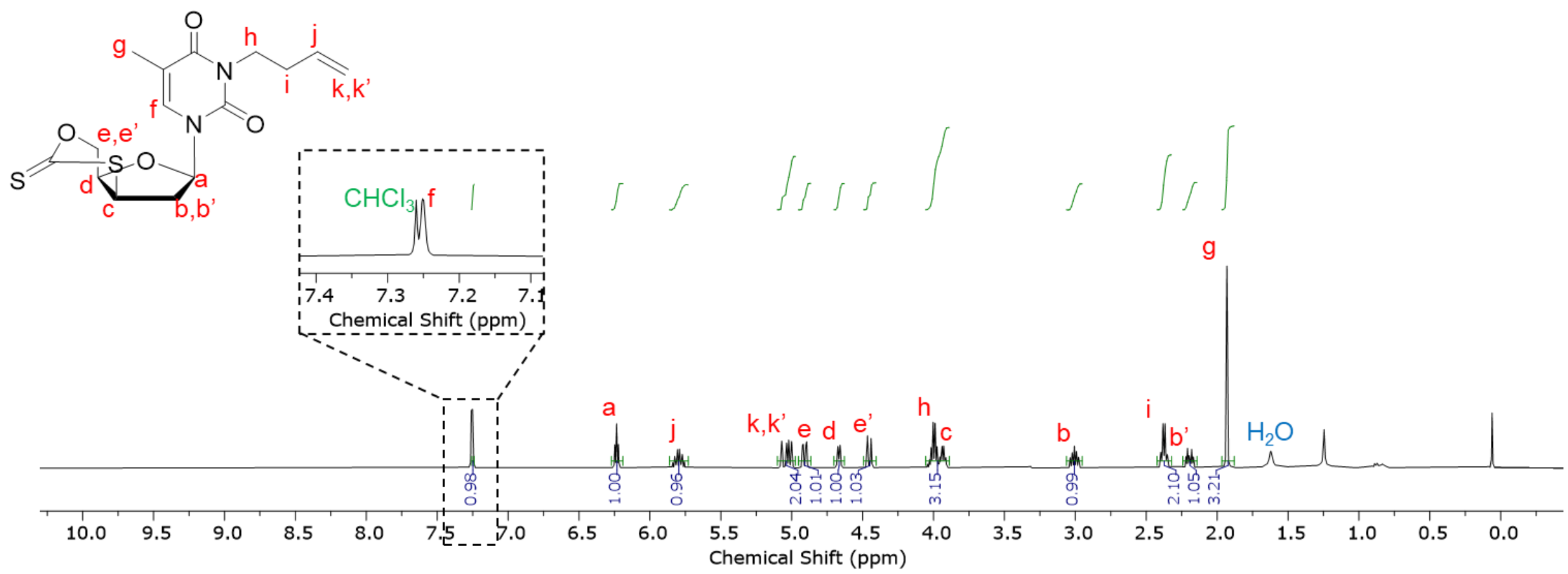

126 MHz – $CDCl_3$

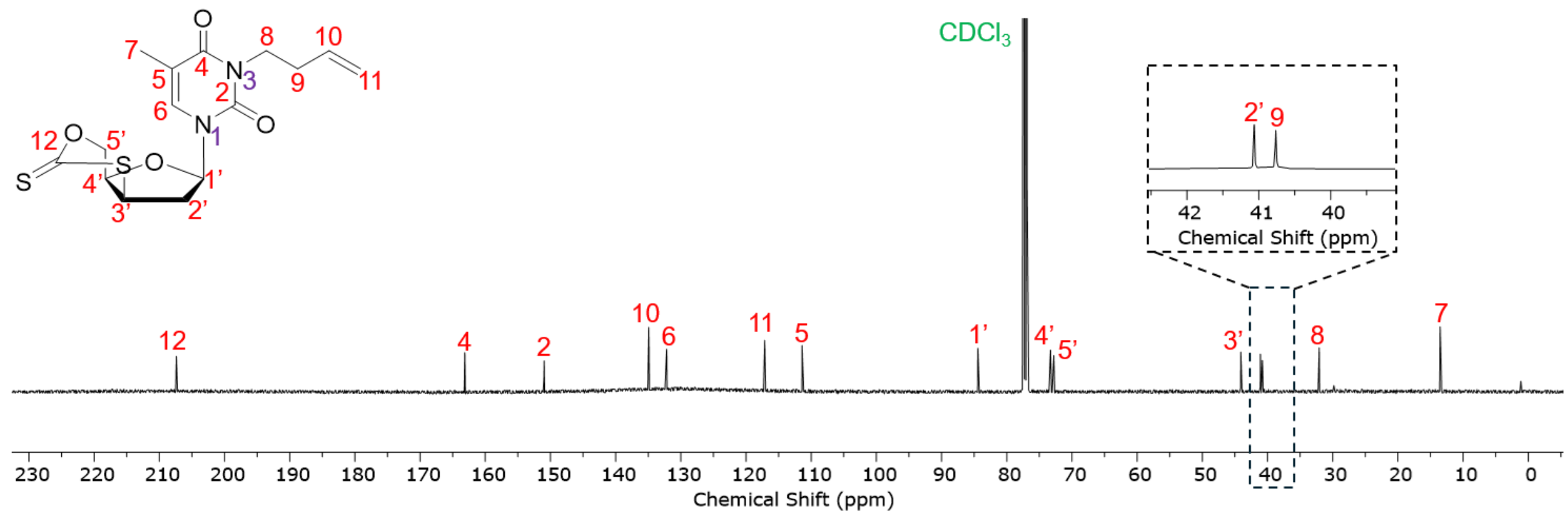

Figure S3. ^{1}H NMR (500 MHz, $CDCl_3$) and ^{13}C NMR (126 MHz, $CDCl_3$) spectra of **3**.

500 MHz – $CDCl_3$

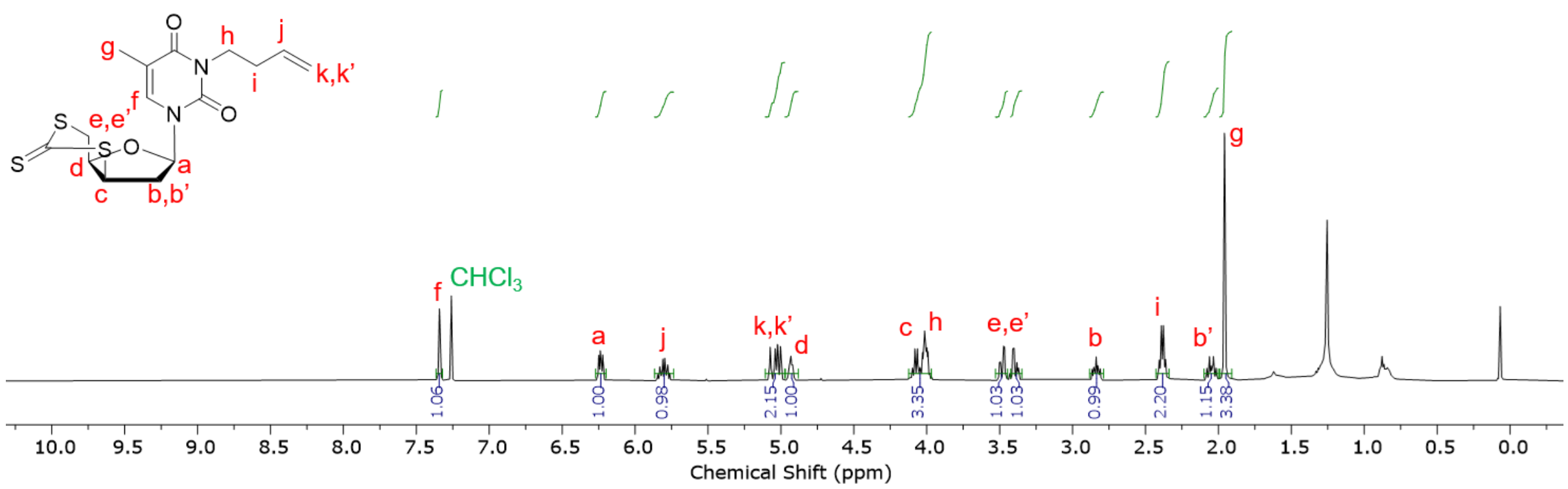

126 MHz – $CDCl_3$

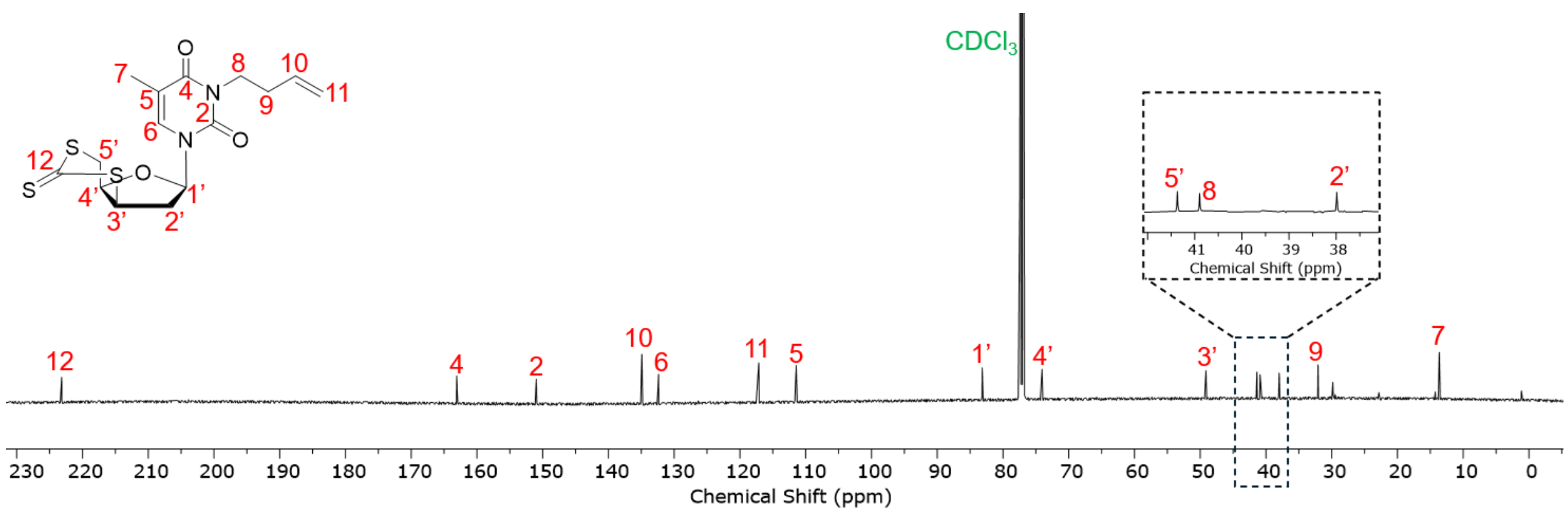

Figure S4. ^{1}H NMR (500 MHz, $CDCl_3$) and ^{13}C NMR (126 MHz, $CDCl_3$) spectra of **5**.

500 MHz – CDCl₃

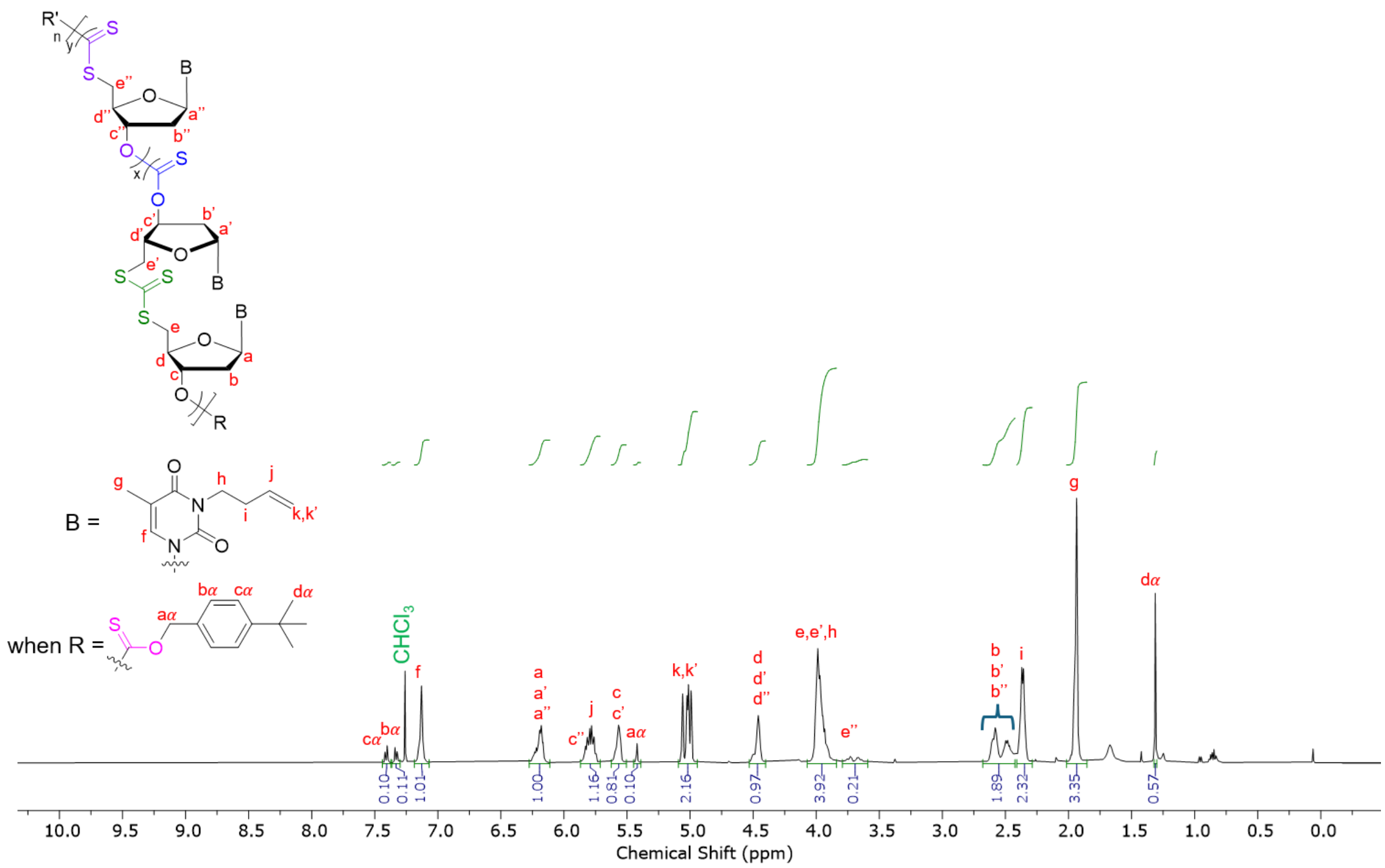

126 MHz – CDCl₃

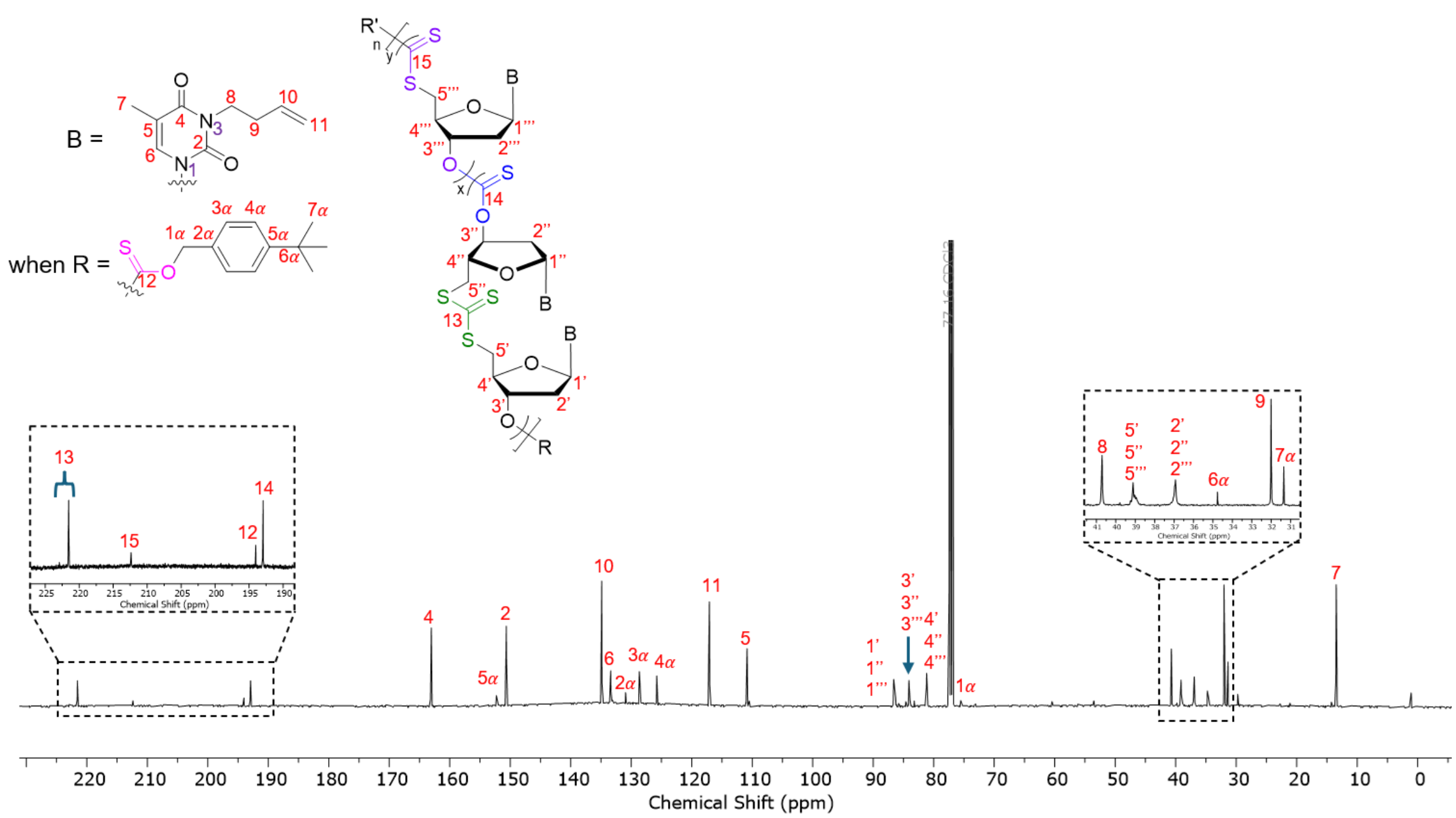

Figure S5. ^{1}H NMR (500 MHz, $CDCl_3$) and ^{13}C NMR (126 MHz, $CDCl_3$) spectra of **P1**$_{r.t.}$

500 MHz – $CDCl_3$

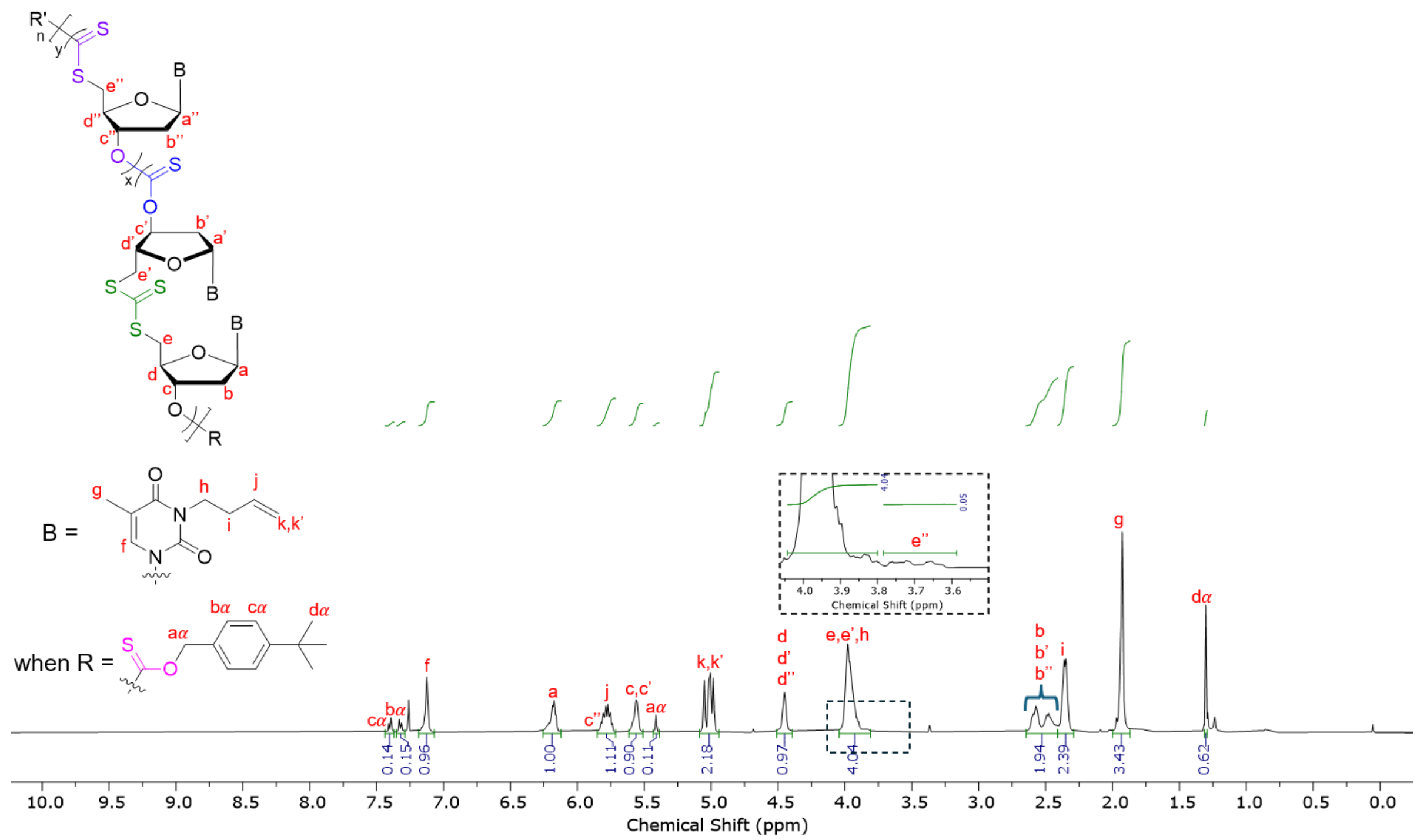

126 MHz – $CDCl_3$

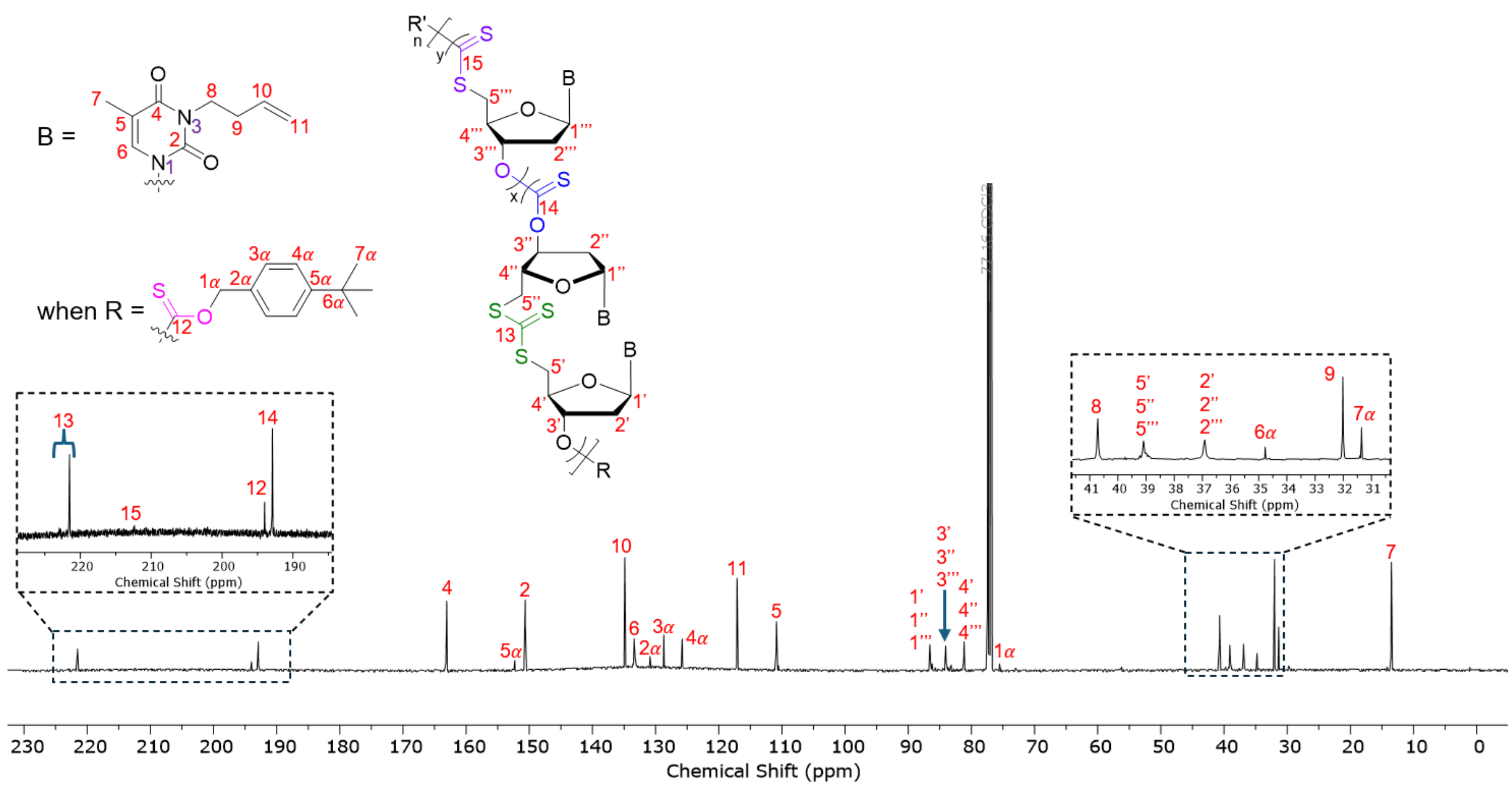

Figure S6. ^1H NMR (500 MHz, $CDCl_3$) and ^{13}C NMR (126 MHz, $CDCl_3$) spectra of **P2$_{r.t.}$**.

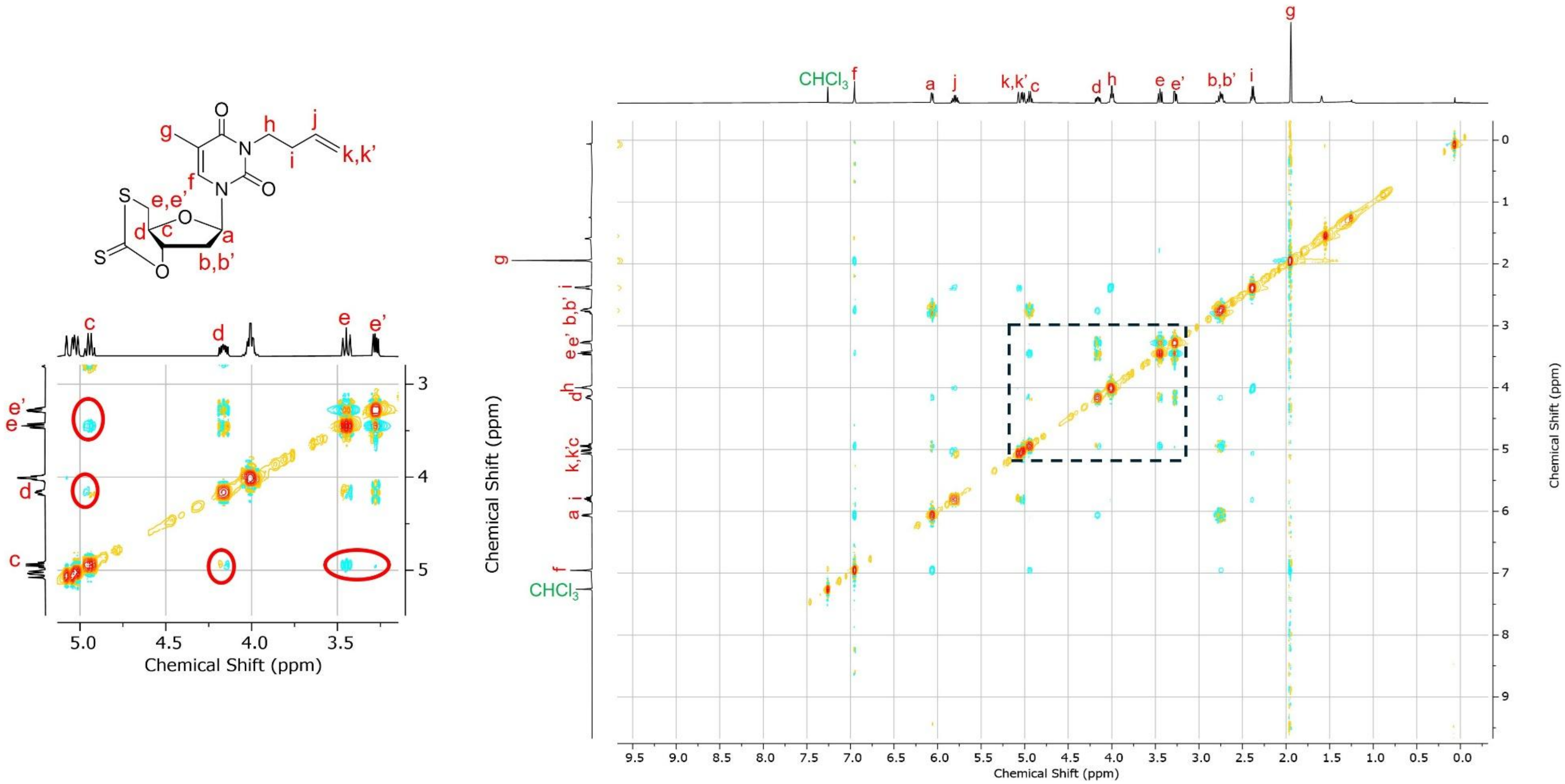

Figure S7. Full and expanded views of NOESY (500 MHz, $CDCl_3$) spectrum of **1**.

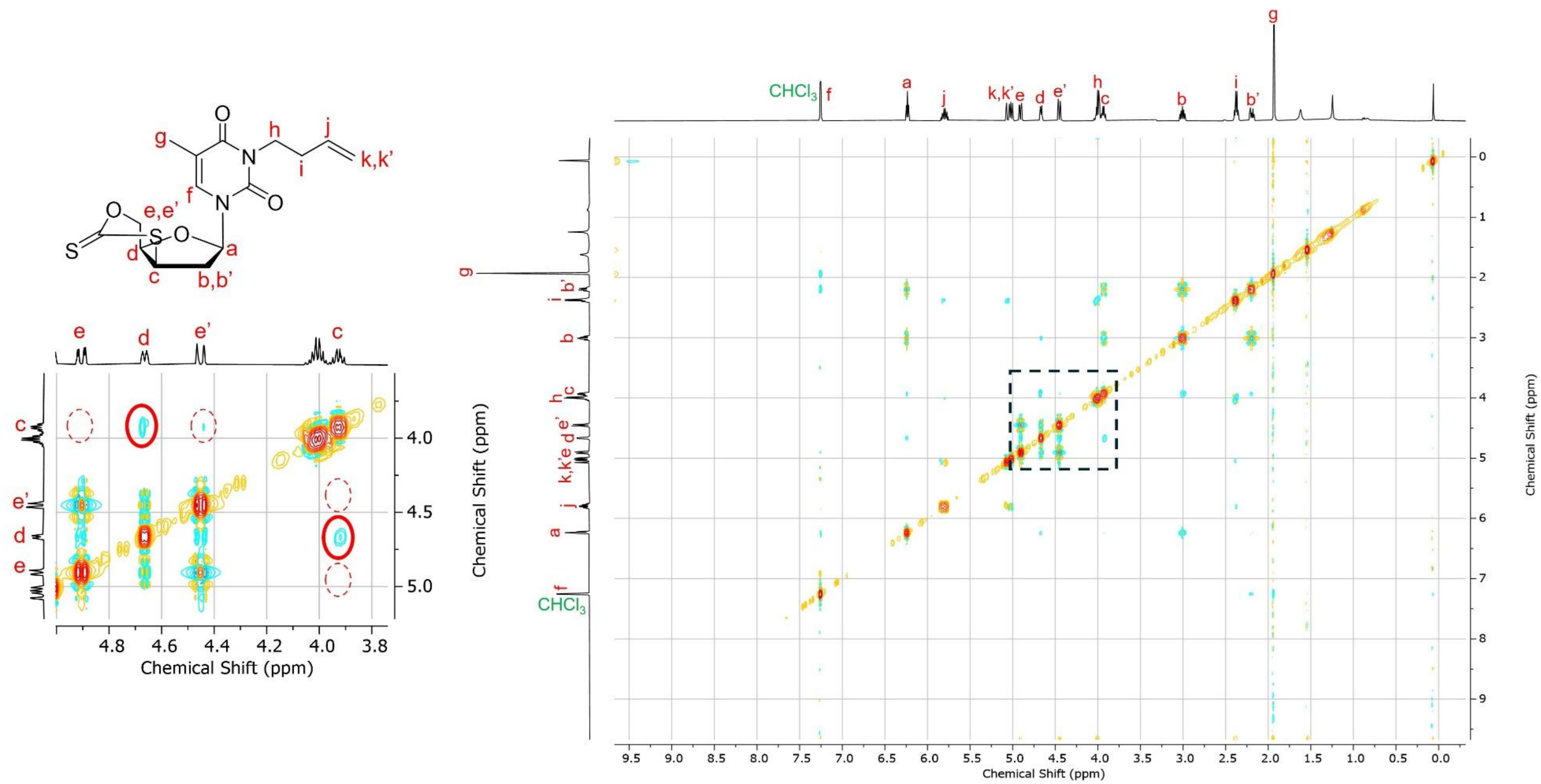

Figure S8. Full and expanded views of NOESY (500 MHz, $CDCl_3$) spectrum of **3**.

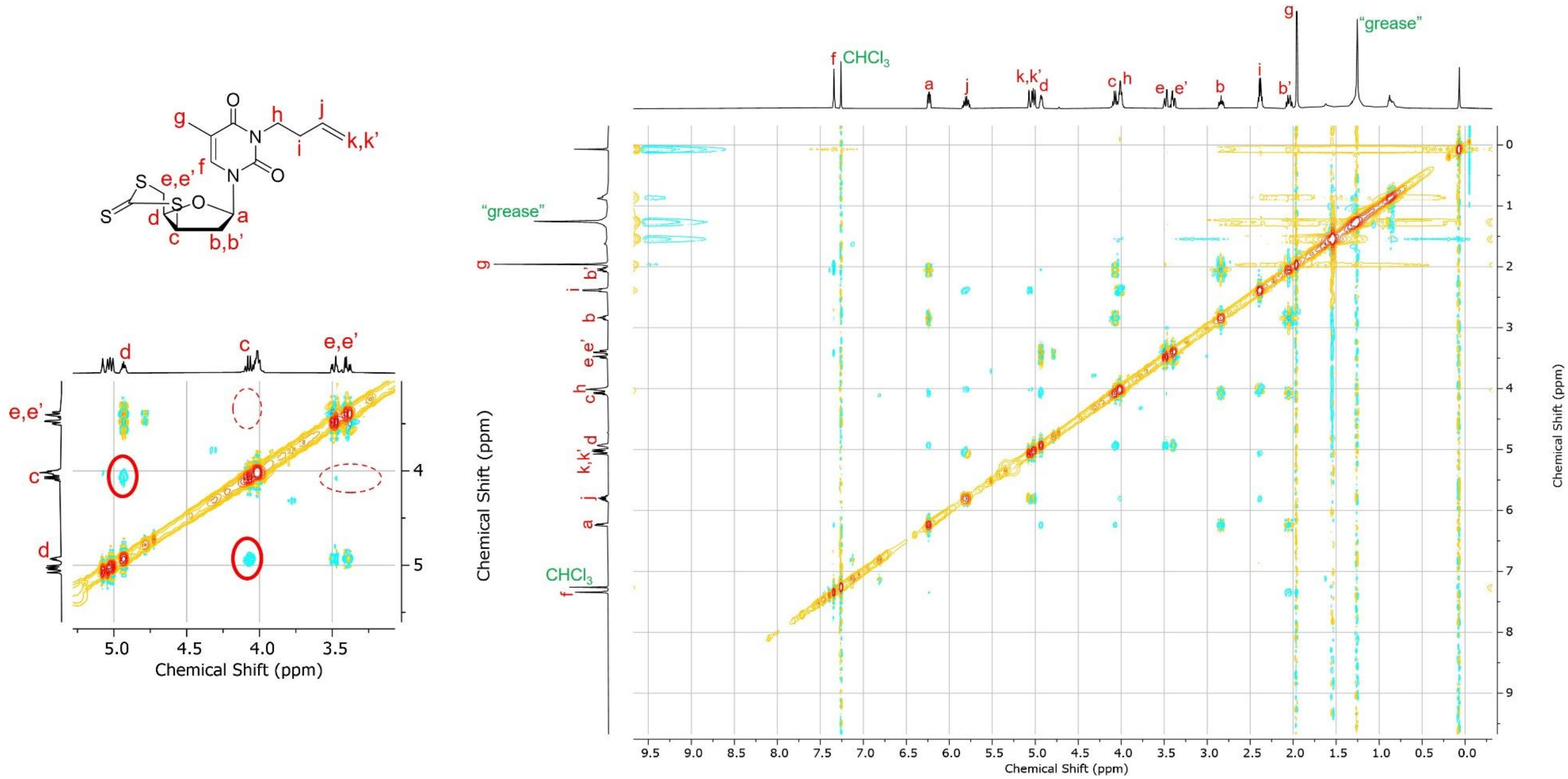

Figure S9. Full and expanded views of NOESY (500 MHz, $CDCl_3$) spectrum of **5**.

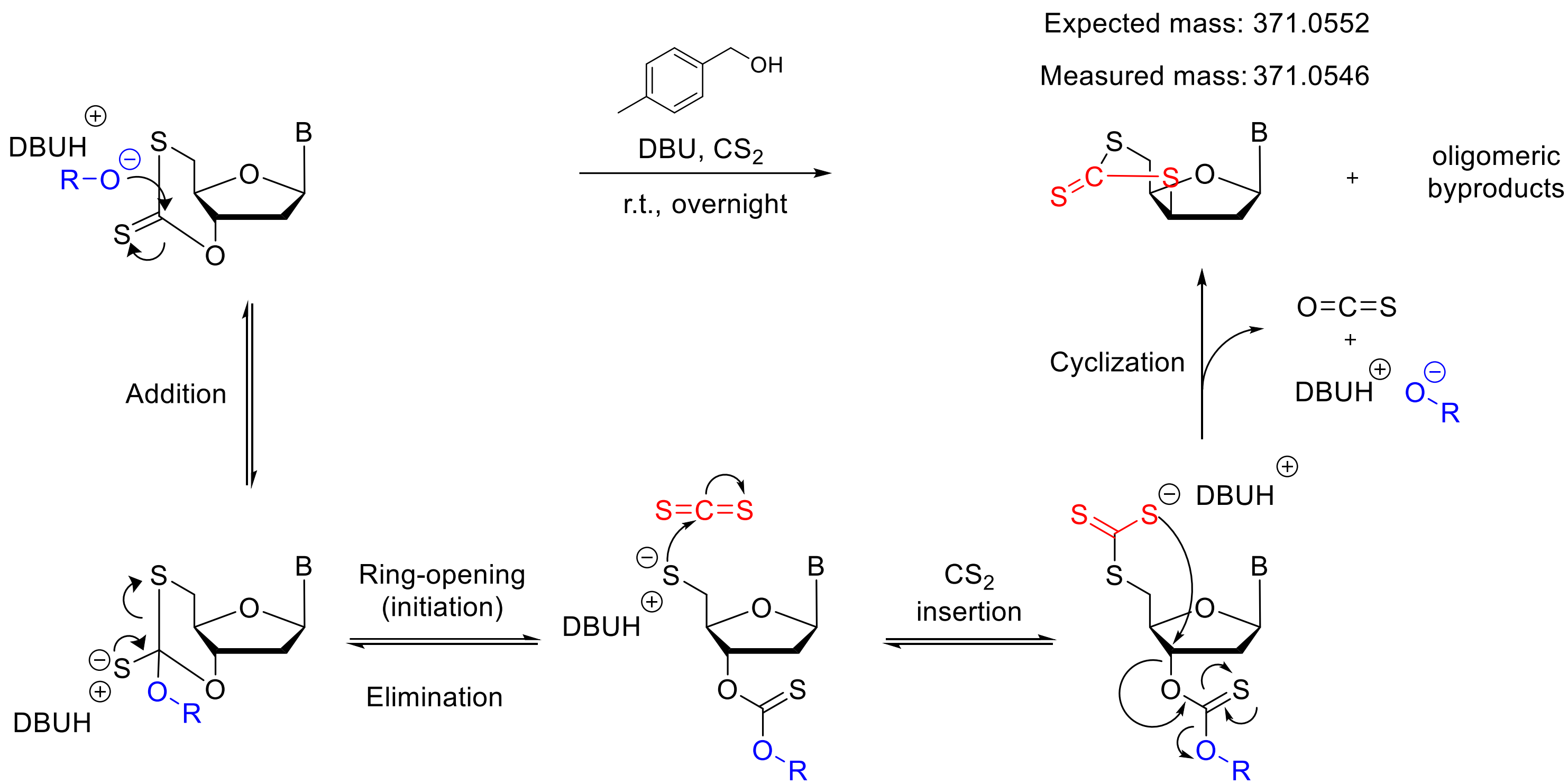

Scheme S1. Proposed mechanism for transformation of **1** to **5**.

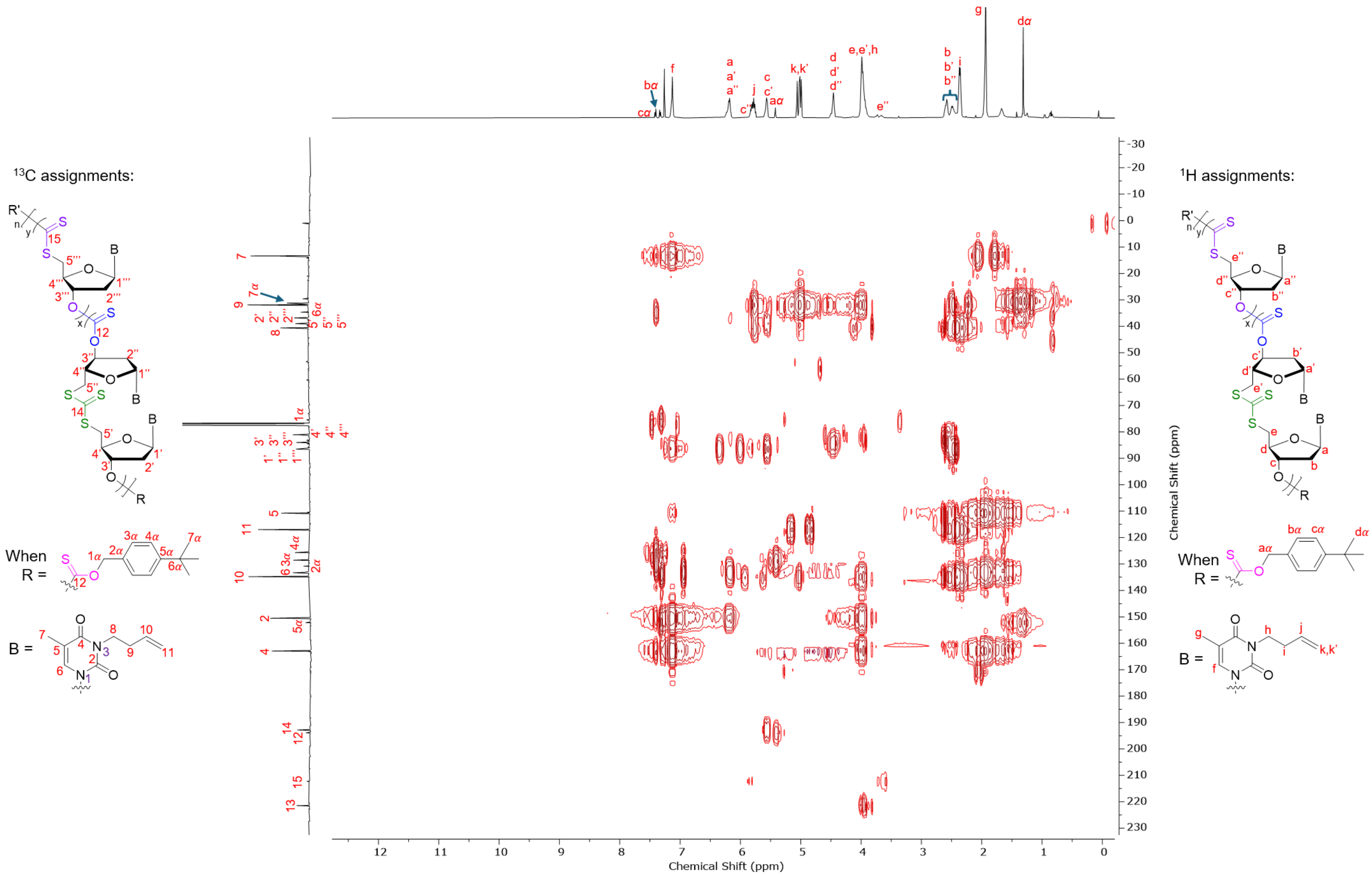

Figure S10. Full view of HMBC (^{1}H NMR 500 MHz, ^{13}C NMR 126 MHz, $CDCl_3$) spectrum of **P1**$_{r.t.}$

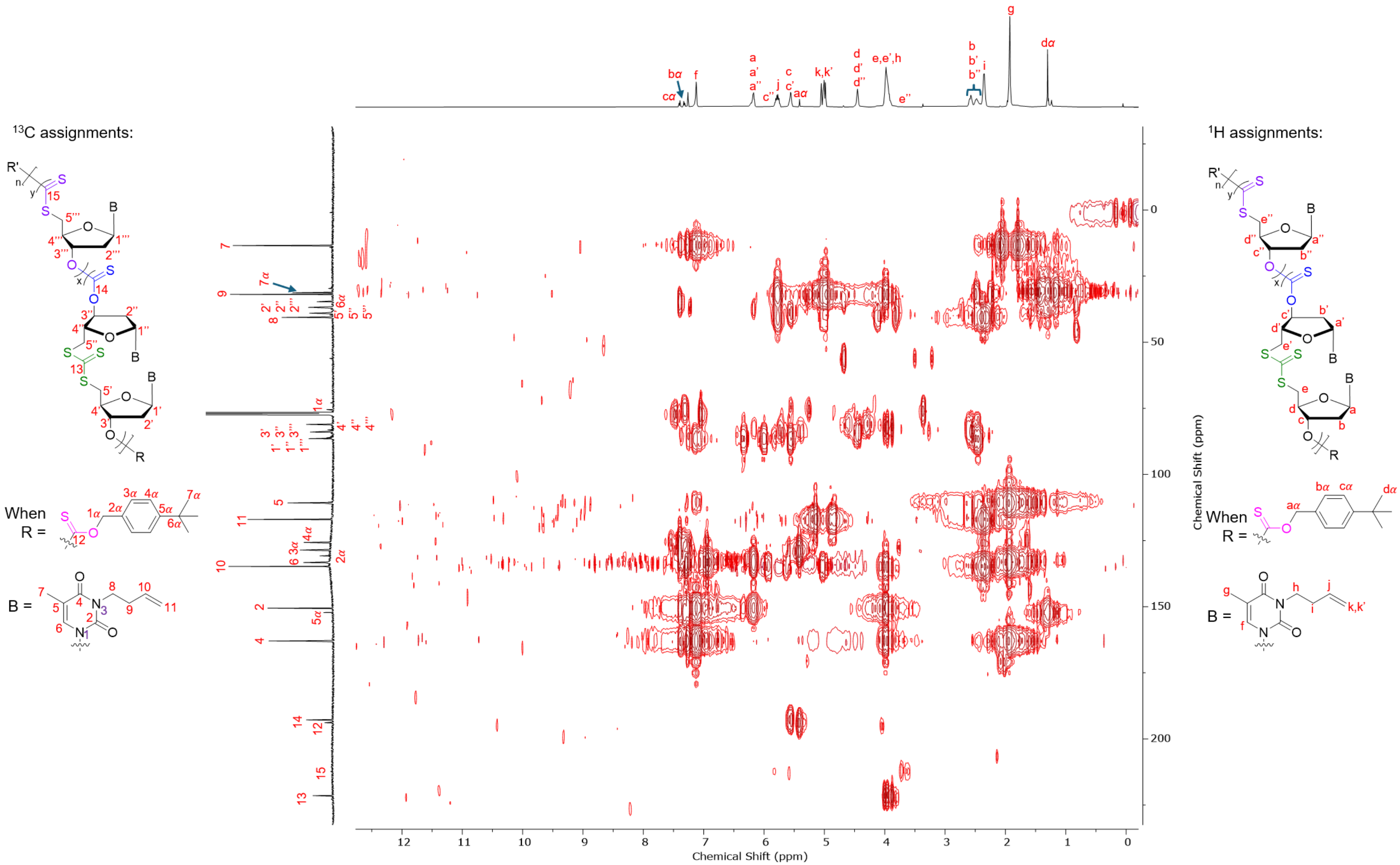

Figure S11. Full view of HMBC (^{1}H NMR 500 MHz, ^{13}C NMR 126 MHz, $CDCl_3$) spectrum of **P2**$_{r.t.}$

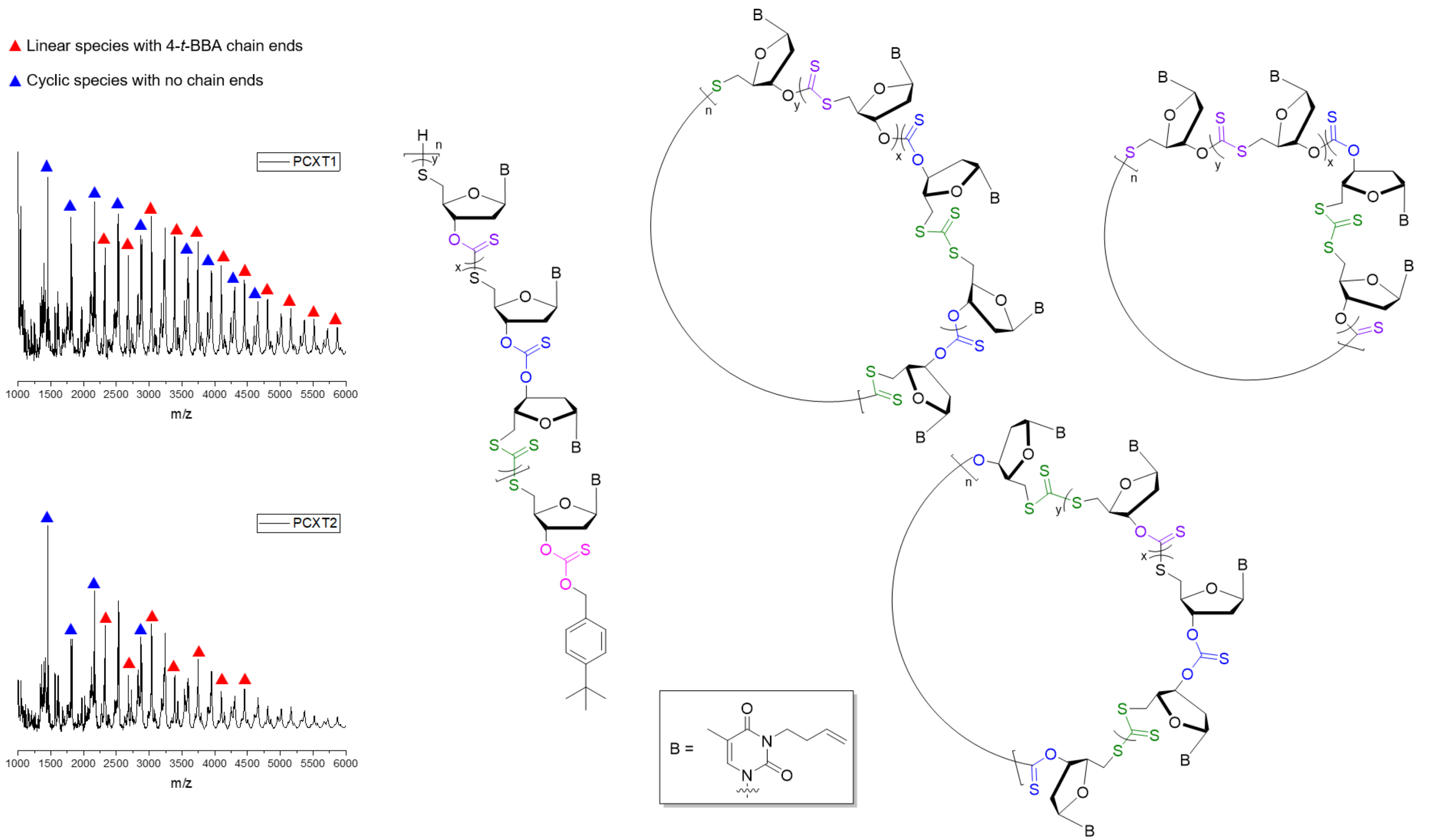

Figure S12. MALDI-TOF spectra of **P1**$_{r.t.}$ and **P2**$_{r.t.}$ and proposed linear and cyclic polymeric structures.

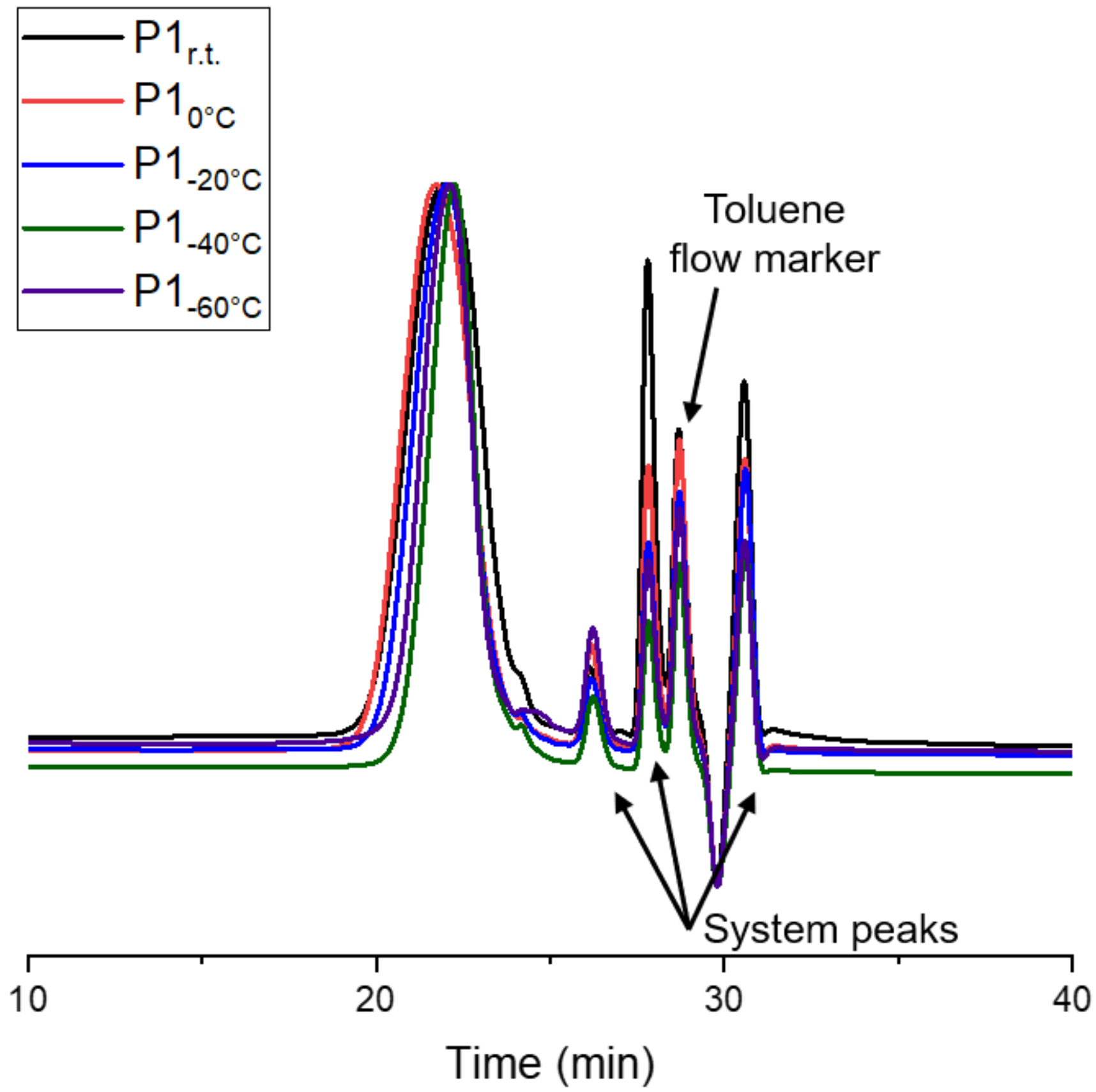

Figure S13. SEC traces (THF as eluent, 1 mL/min) of **P1** polymers generated from ROP of **1** in the presence of DBU at varying temperatures.

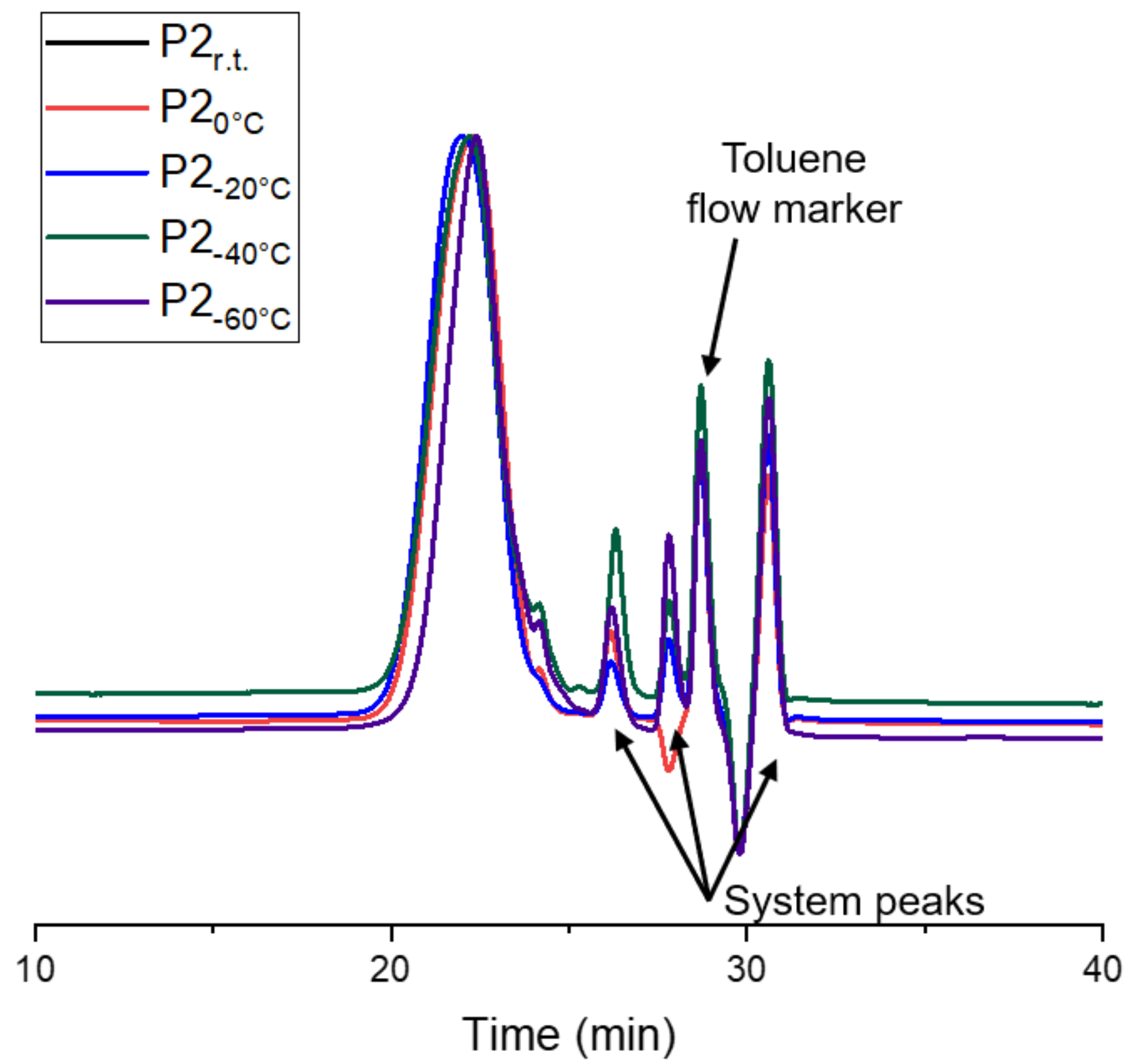

Figure S14. SEC traces (THF as eluent, 1 mL/min) of **P2** polymers generated from ROP of **1** in the presence of TBD at varying temperatures.

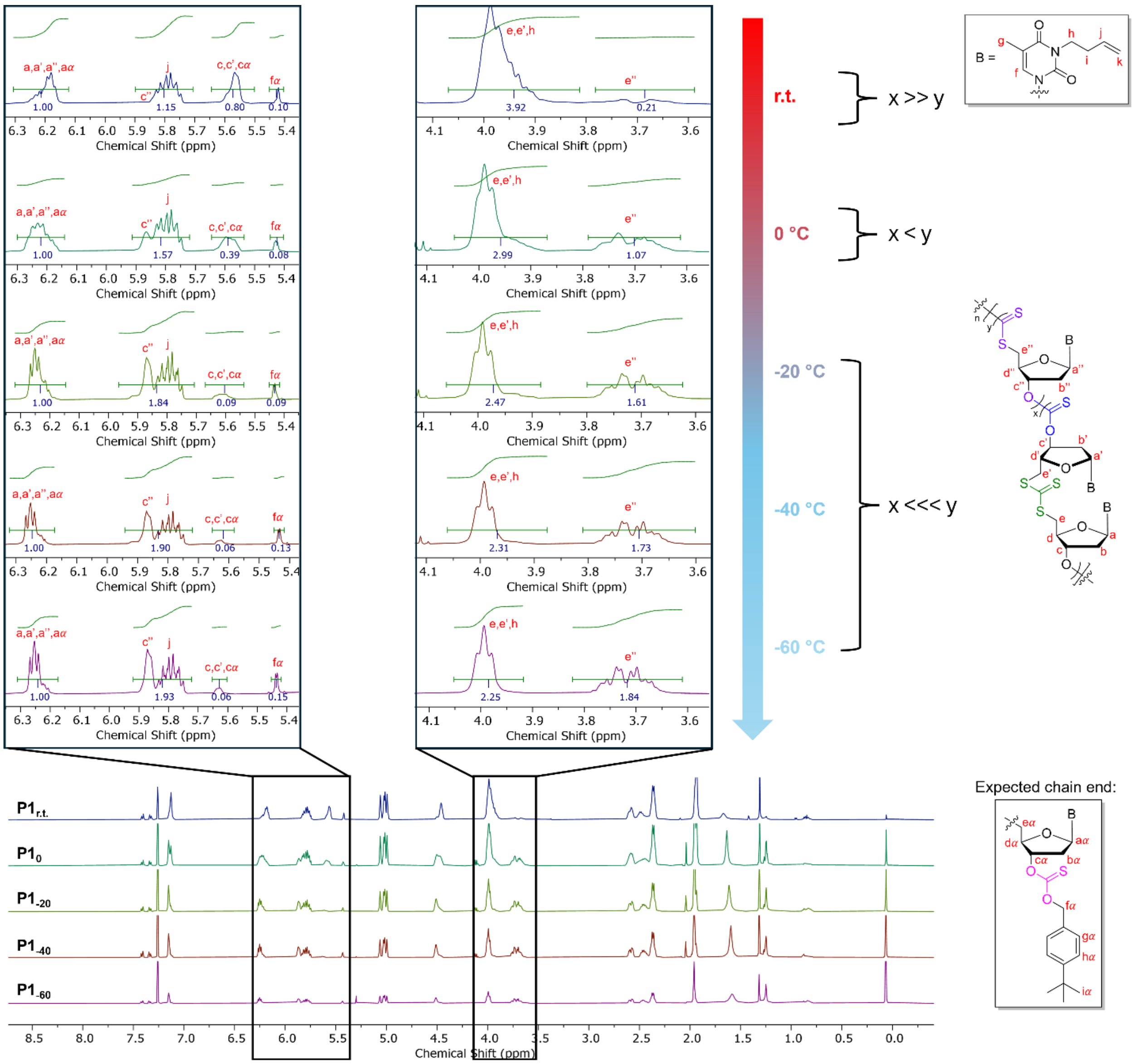

Figure S15. Full and expanded views of ^{1}H NMR (500 MHz, $CDCl_3$) for all **P1** polymers, prepared from **1** using DBU as the organobase catalyst.

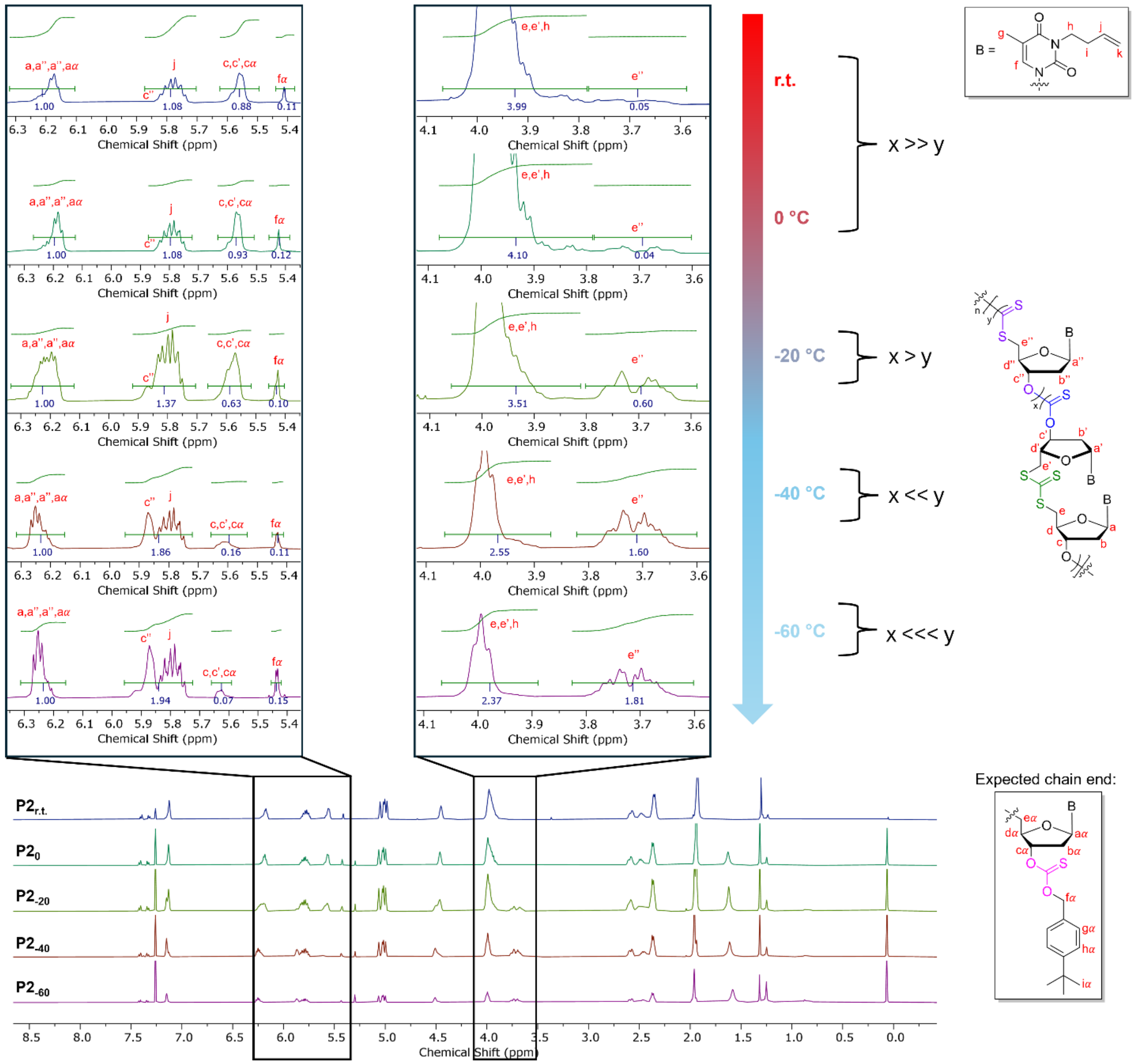

Figure S16. Full and expanded views of ^{1}H NMR (500 MHz, $CDCl_3$) for all **P2** polymers, prepared from **1** using TBD as the organobase catalyst.

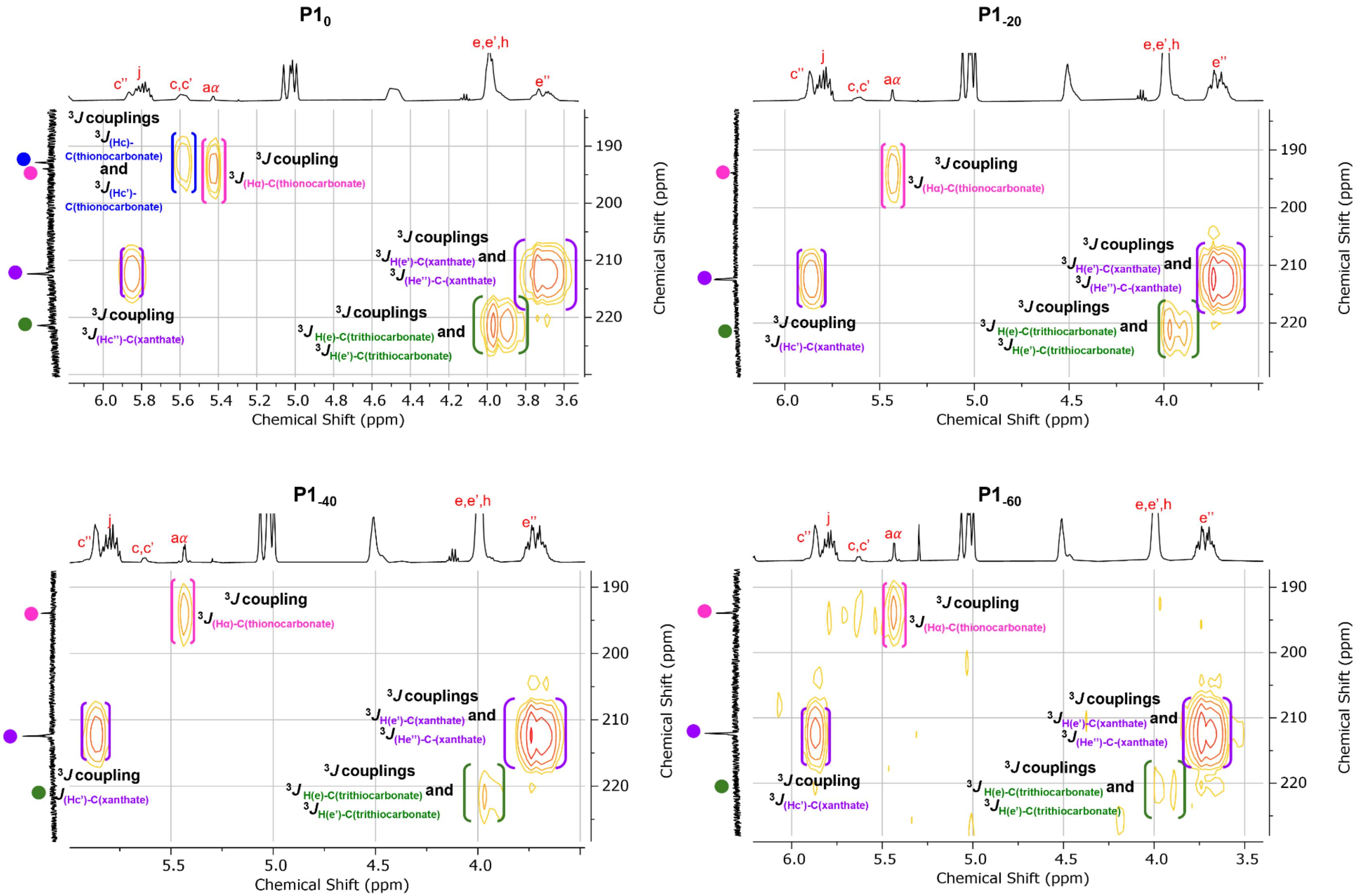

Figure S17. Expanded views of thiocarbonate region in the HMBC spectra for **P1** polymers.

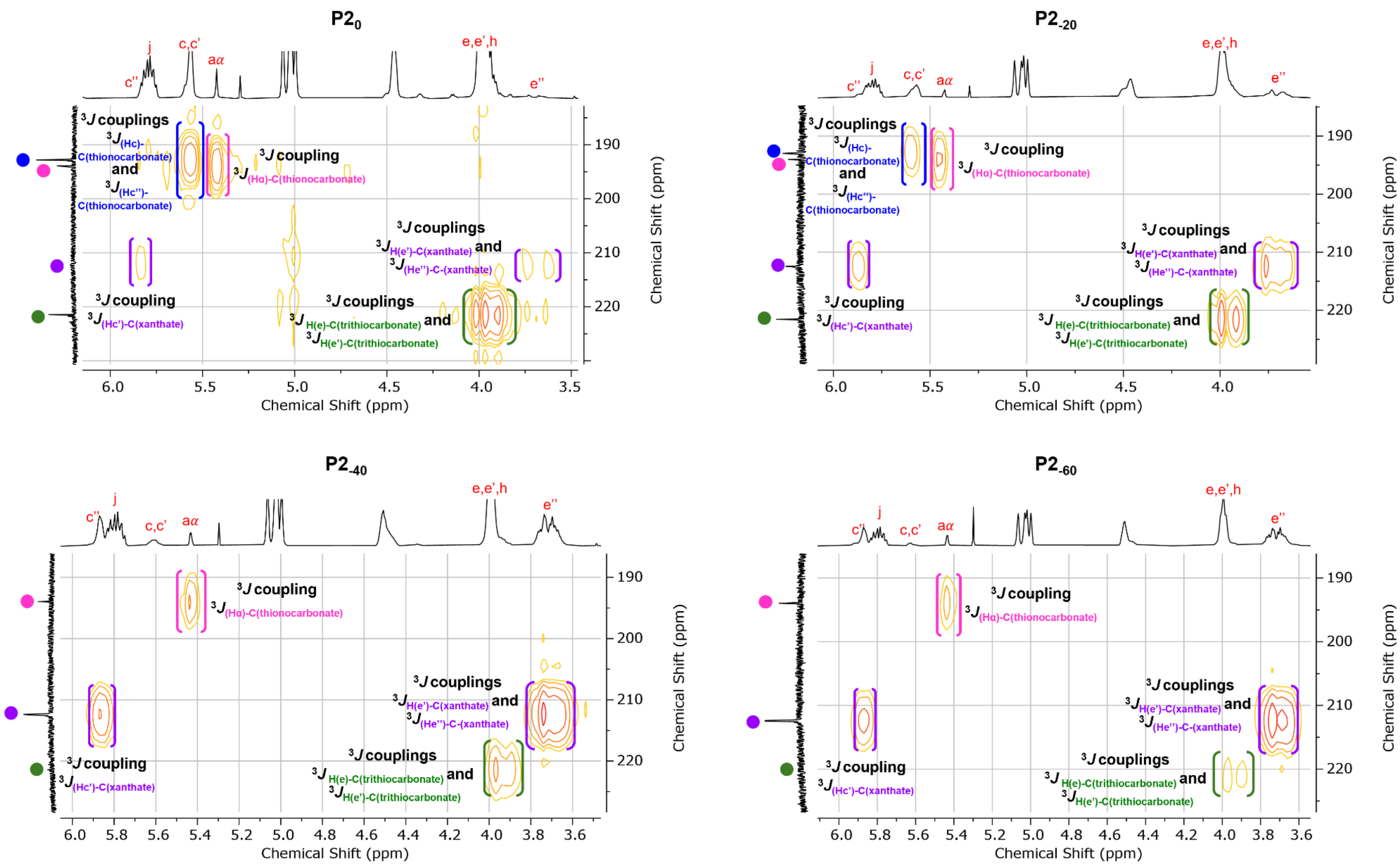

Figure S18. Expanded views of thiocarbonate region in the HMBC spectra for **P2** polymers.

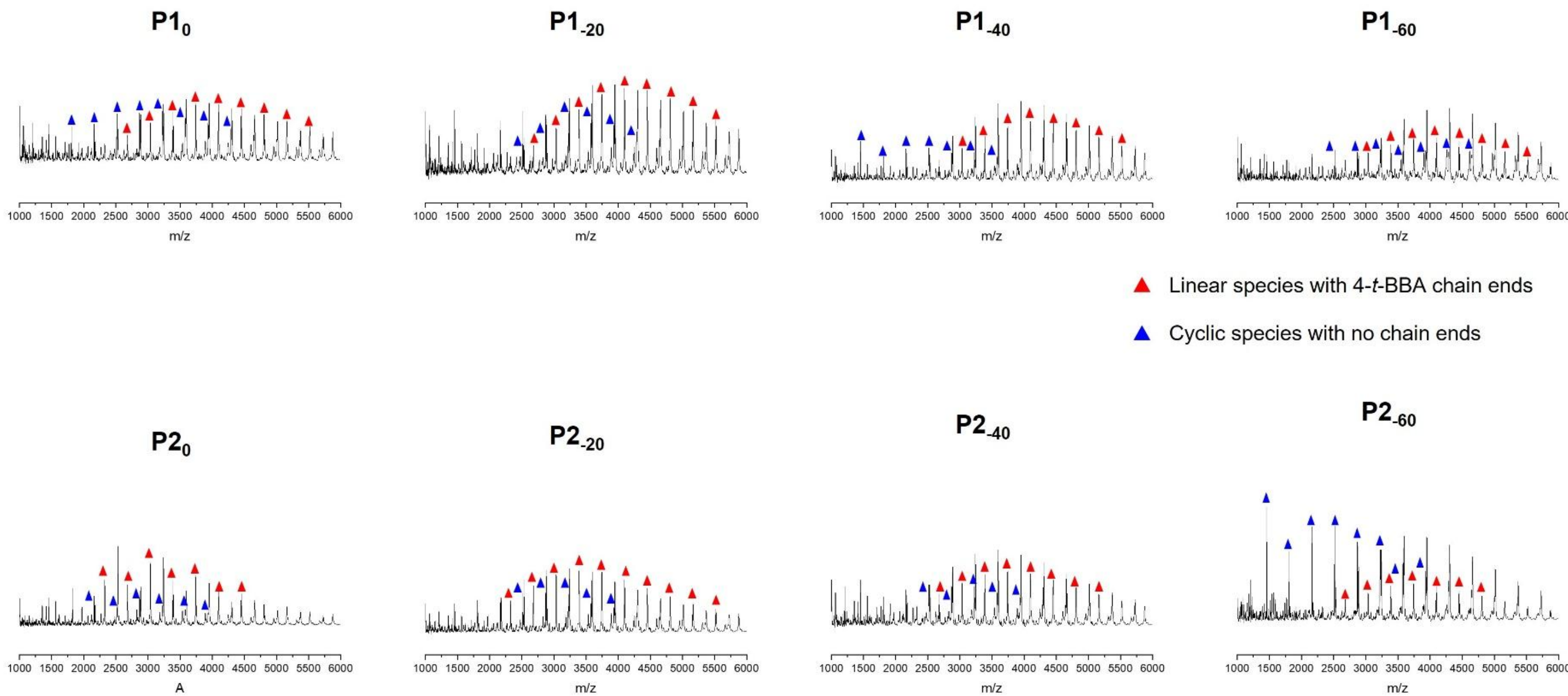

Figure S19. MALDI-TOF spectra for purified **P1** and **P2** polymers.

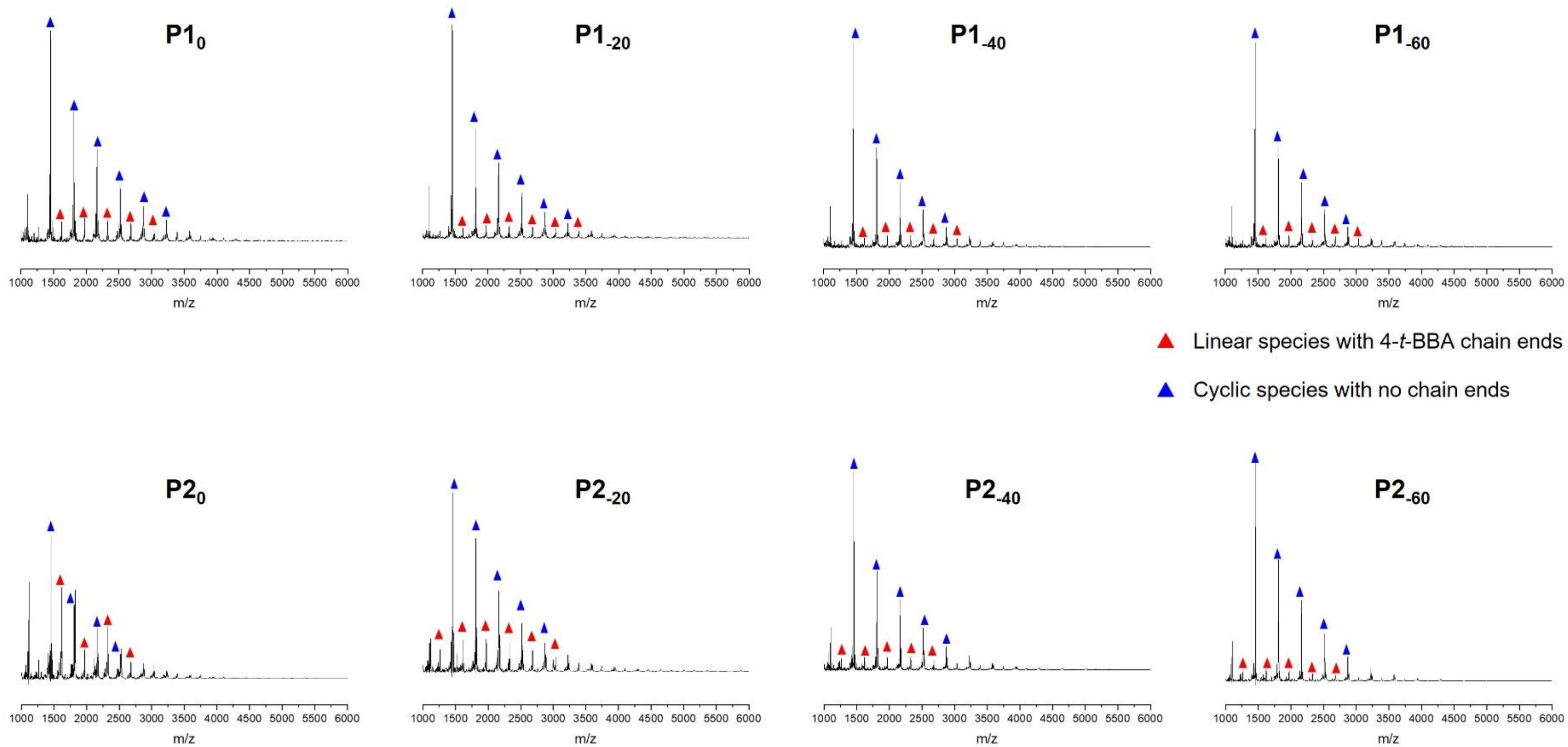

Figure S20. MALDI-TOF spectra for supernatants of purified **P1** and **P2** polymers.

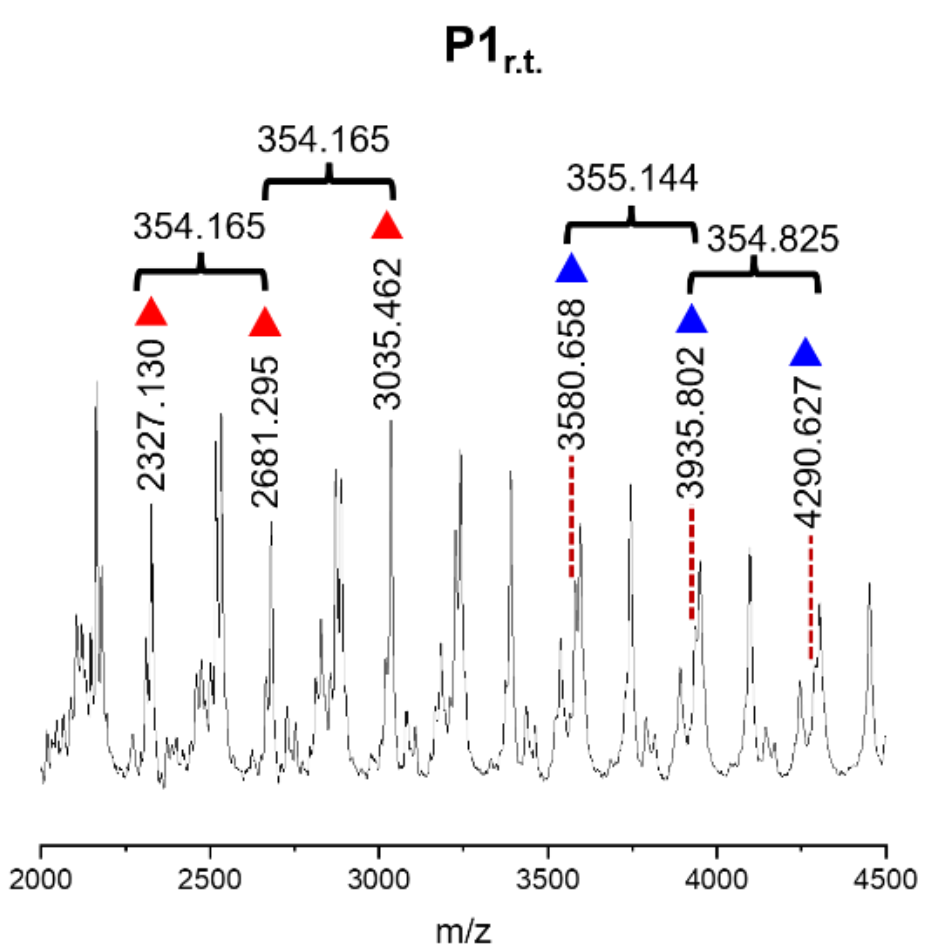

n	Calc.	Found
6	2329.88	2327.130
7	2684.32	2681.295
8	3038.76	3035.462

n	Calc.	Found
10	3583.40	3580.658
11	3937.84	3935.802
12	4292.28	4290.627

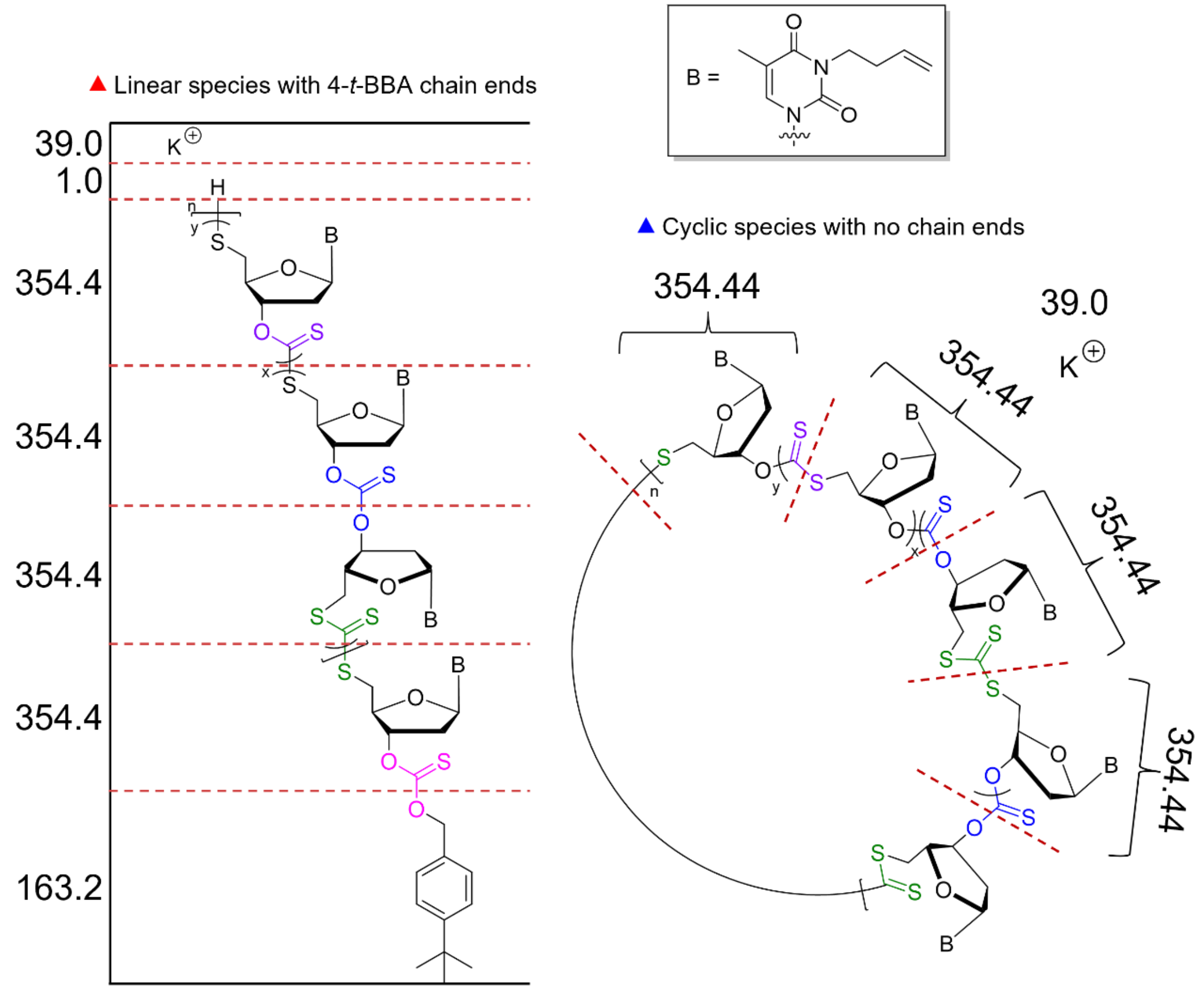

Figure S21. Expanded view of **P1**$_{r.t.}$ MALDI-TOF spectrum to demonstrate repetition of m/z values for proposed linear and cyclic species having primarily trithiocarbonate-*co*-thionocarbonate dimeric repeating units.

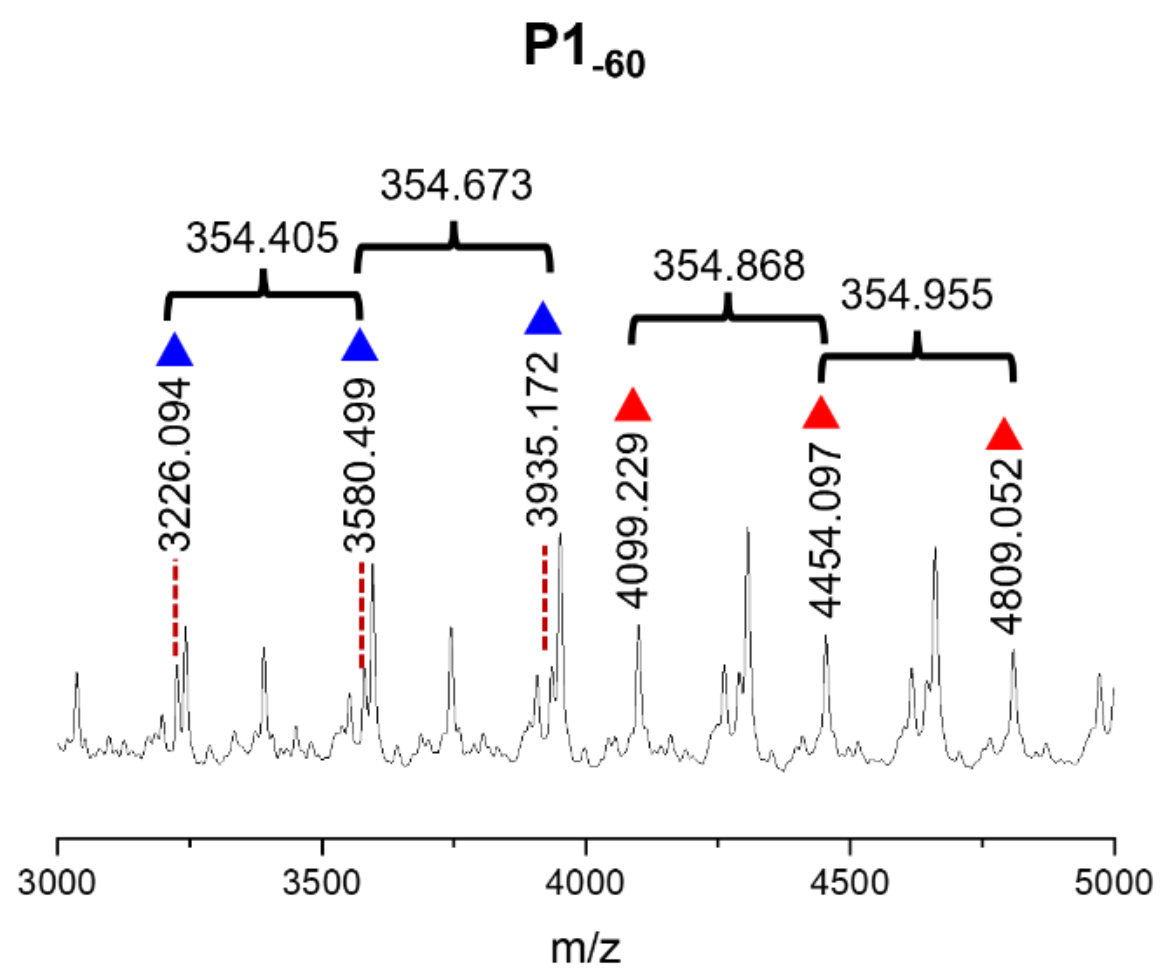

n	Calc.	Found
11	4102.08	4099.229
12	4456.52	4454.097
13	4810.096	4809.052

n	Calc.	Found
9	3228.96	3226.094
10	3583.40	3580.499
11	3937.84	3935.172

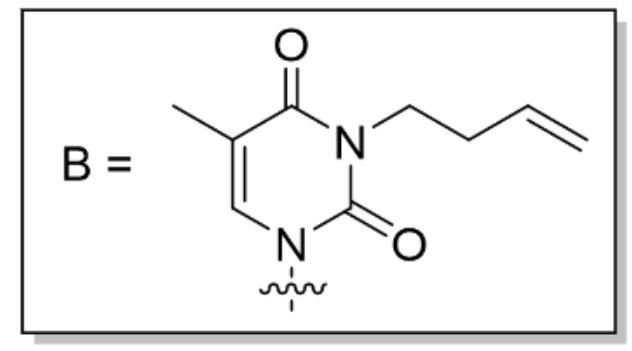

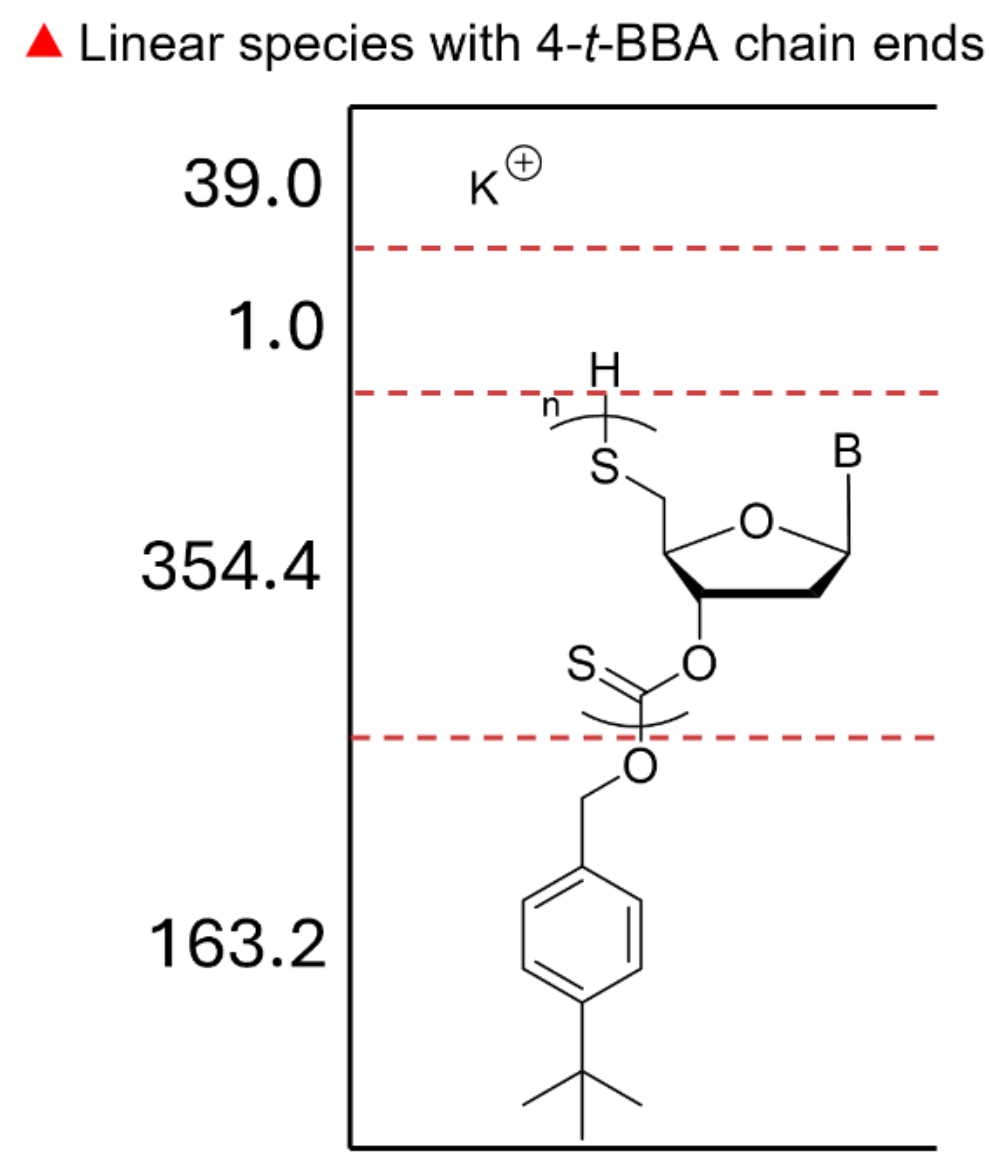

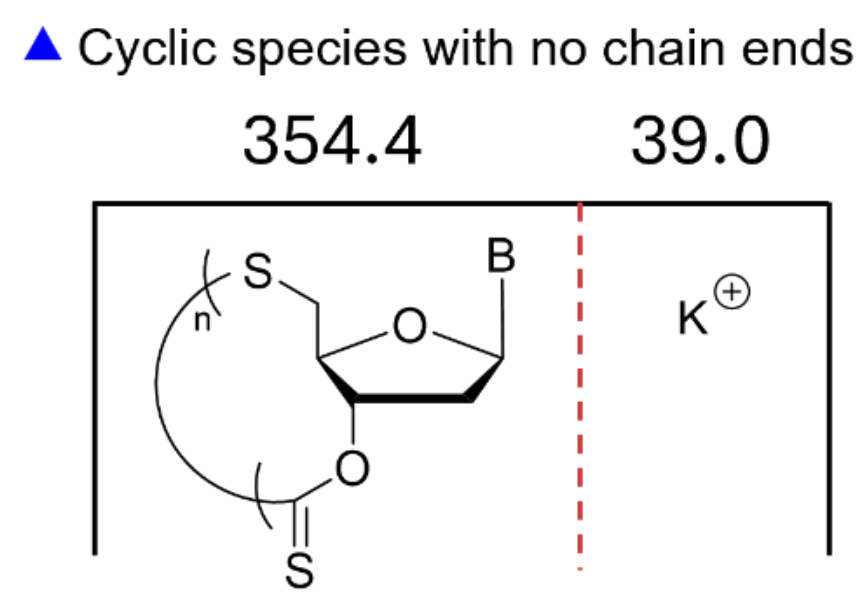

Figure S22. Expanded view of **P1-60** MALDI-TOF spectrum to demonstrate repetition of m/z values for proposed linear and cyclic species

III. Computational Details and Related Figures, Table, and Data

The ring strain energies were evaluated by calculating the enthalpies of specific isodesmic ring-opening reactions. In these computational models, each cyclic molecule (**Figure S23**) reacts with a corresponding small acyclic reference molecule—dimethyl carbonate for cyclic carbonates, dimethyl trithiocarbonate for cyclic trithiocarbonates, and dimethyl xanthate for cyclic xanthates—to afford a fully acyclic product (**Figure S24**). This approach conserves the number and types of chemical bonds, yielding acyclic structures that accurately reflect the actual functional linkages present within the corresponding polymer backbones.

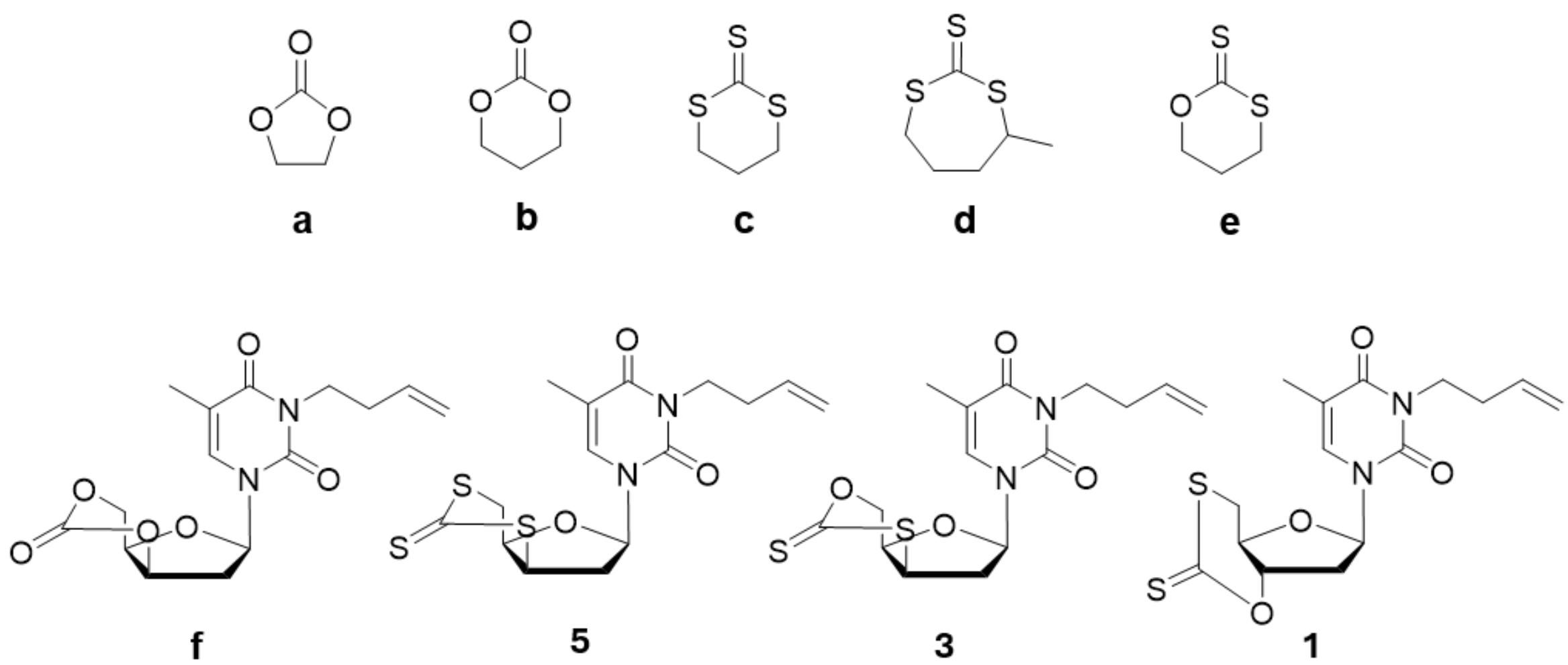

Figure S23. Cyclic monomers for DFT calculations of ring strains.

To ensure an accurate representation of the thermodynamic properties for the flexible cyclic and acyclic molecules, the initial geometries were optimized at the GFN2-xTB level of theory, followed by a subsequent conformer ensemble generation using CREST (version 2.8).[3, 4] The resulting lowest-energy 10 conformers of each molecule were subsequently subjected to DFT-level optimization. All DFT calculations were performed using Gaussian (version 16 C.01).[5] The geometric optimization and harmonic vibrational frequency calculations were carried out at B3LYP/6-311G(d,p) level, incorporating Grimme's D3 empirical dispersion correction (GD3). The absence of imaginary frequencies confirmed that all optimized structures represent local minima on the potential energy surface. Cartesian coordinates of all the stationary points are available in xyz format in the zip files.

To obtain more accurate electronic energies, single-point energy calculations were performed on the optimized geometries at the ωB97X-D and M062X/6-31^{++}G(d,p) level of theory. Solvent effects (dichloromethane) were implicitly accounted for during the single-point calculations using the SMD solvation model. Thermochemical corrections at 298.15 K were computed utilizing the GoodVibes program (version 4.3.0),[6, 7] which applies a quasi-harmonic approximation (mRRHO) to correct the entropy contributions of low-frequency vibrational modes. The final reported enthalpies and Gibbs free energies correspond to the Boltzmann-weighted ensemble averages of 10 conformers (**Appendix 1 & 2**), providing a rigorous representation of the macroscopic thermodynamic states.

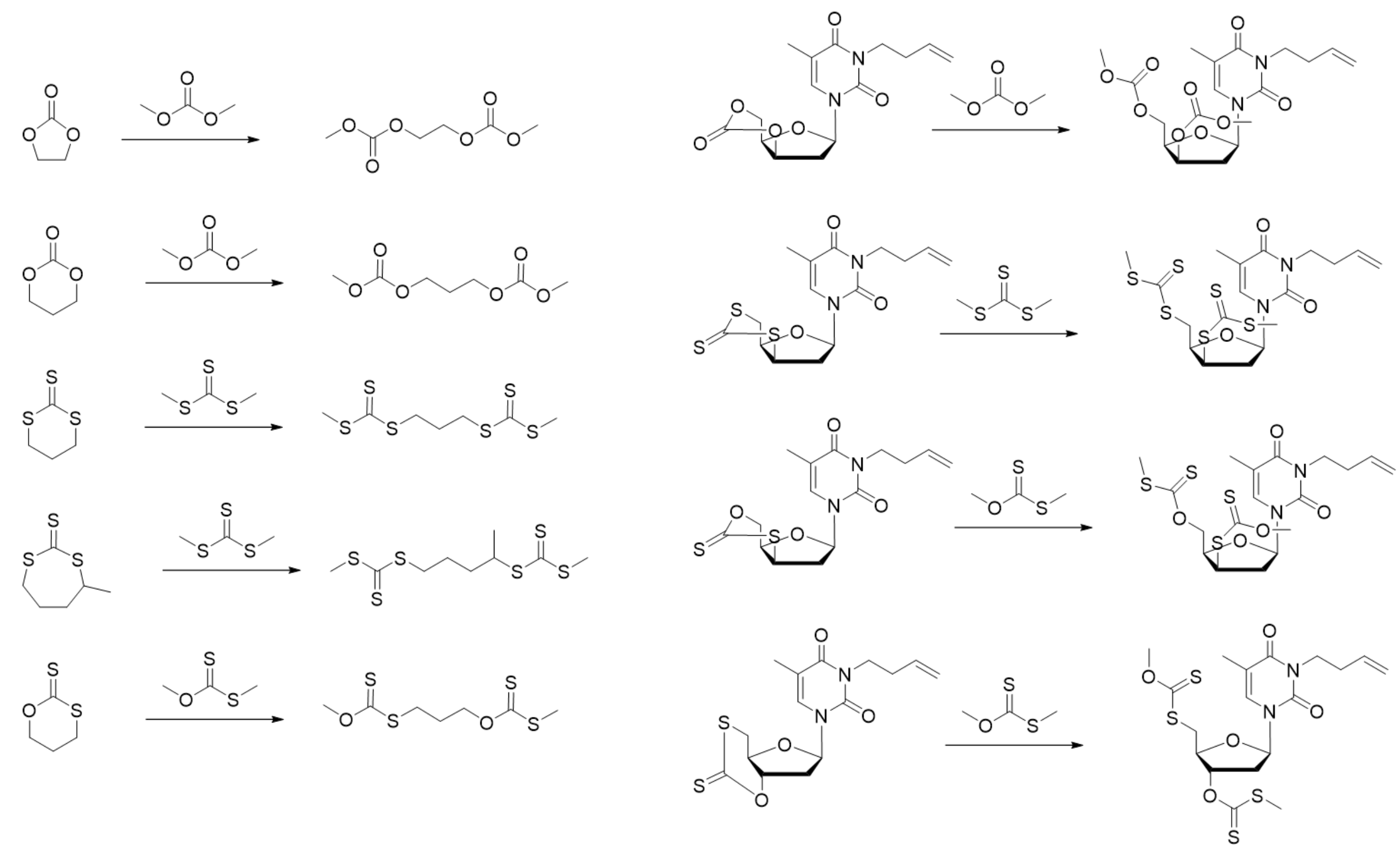

Figure S24. Consideration of ring strains by calculations of enthalpies of isodesmic ring-opening reactions.

Table S1. DFT calculations of the ring strain energies (kcal/mol) of different cyclic carbonates, cyclic trithiocarbonates and cyclic xanthates, with the chemical structures shown in Figure S23, and the polymerizability designations made according to the references cited.

Chemical Structure	Calculated Enthalpy value for the Isodesmic Ring Opening Process [i]		Polymerizable?
	ωb97X-D/6-31^{++}G (d,p)	M062X/6-31^{++}G (d,p)	
**a**[8]	4.63	7.93	✗
**b**[9]	7.88	9.89	✓
**c**[10]	1.99	3.99	✗
**d**[10]	7.11	8.52	✓
**e**[ii]	0.96	3.03	?
**f**[11]	8.14	9.55	✓
**5**	1.21	3.76	✗
**3**	6.46	7.44	✗
**1**	7.67	8.42	✓

i) Averaged from the top 10 lowest-energy conformers.
ii) No literature reference found regarding experimental polymerizability.

Appendix 1. Ensemble average of enthalpies and free energies of each conformation under ωB97X-D/6-31++G(d,p) level.

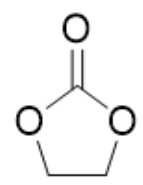

Structure	H (Hartree)	qh-G (Hartree)	Pop (%)
5carbonate_best.log	-343.447094	-343.477156	100.00

Boltzmann-Averaged Enthalpy (H) : -343.447094 Hartree
Boltzmann-Averaged Free Energy (G) : -343.477156 Hartree

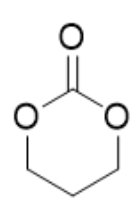

Structure	H (Hartree)	qh-G (Hartree)	Pop (%)
6carbonate_best.log	-381.477257	-381.511258	100.00

Boltzmann-Averaged Enthalpy (H) : -381.477257 Hartree
Boltzmann-Averaged Free Energy (G) : -381.511258 Hartree

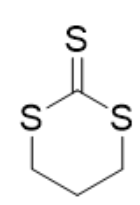

Structure	H (Hartree)	qh-G (Hartree)	Pop (%)
conf_1.log	-1350.417118	-1350.457914	7.78
conf_2.log	-1350.419219	-1350.460249	92.22

Boltzmann-Averaged Enthalpy (H) : -1350.419056 Hartree
Boltzmann-Averaged Free Energy (G) : -1350.460068 Hartree

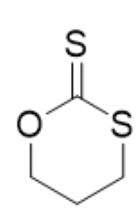

Structure	H (Hartree)	qh-G (Hartree)	Pop (%)
6xanthate_best.log	-1027.441195	-1027.477766	100.00

Boltzmann-Averaged Enthalpy (H) : -1027.441195 Hartree
Boltzmann-Averaged Free Energy (G) : -1027.477766 Hartree

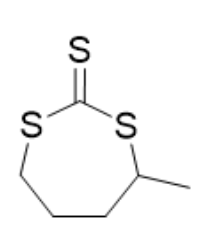

Structure	H (Hartree)	qh-G (Hartree)	Pop (%)
conf_1_opt.log	-1428.969918	-1429.016164	55.80
conf_2_opt.log	-1428.969506	-1429.015344	23.42
conf_3_opt.log	-1428.968374	-1429.014391	8.53
conf_4_opt.log	-1428.968294	-1429.014587	10.50
conf_5_opt.log	-1428.966991	-1429.012867	1.70
conf_6_opt.log	-1428.963524	-1429.009522	0.05

Boltzmann-Averaged Enthalpy (H) : -1428.969466 Hartree
Boltzmann-Averaged Free Energy (G) : -1429.015596 Hartree

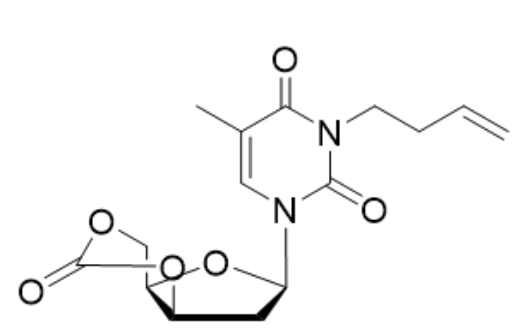

Structure	H (Hartree)	qh-G (Hartree)	Pop (%)
conf_1.log	-1142.700034	-1142.767492	6.57
conf_2.log	-1142.697531	-1142.765266	0.62
conf_3.log	-1142.700342	-1142.769242	41.90
conf_4.log	-1142.700286	-1142.769146	37.83
conf_5.log	-1142.699308	-1142.767260	5.13
conf_6.log	-1142.699318	-1142.767267	5.17
conf_7.log	-1142.697261	-1142.764984	0.46
conf_8.log	-1142.696815	-1142.765554	0.84
conf_9.log	-1142.696740	-1142.765426	0.74
conf_10.log	-1142.696742	-1142.765429	0.74

Boltzmann-Averaged Enthalpy (H) : -1142.700080 Hartree
Boltzmann-Averaged Free Energy (G) : -1142.768755 Hartree

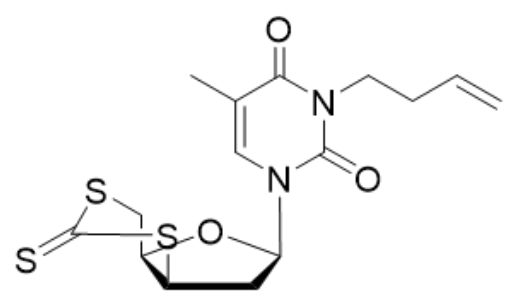

Structure	H (Hartree)	qh-G (Hartree)	Pop (%)
conf_1.log	-2111.597513	-2111.668879	0.95
conf_2.log	-2111.598638	-2111.670086	3.41
conf_3.log	-2111.596453	-2111.667986	0.37
conf_4.log	-2111.598038	-2111.669682	2.22
conf_5.log	-2111.599376	-2111.671907	23.46
conf_6.log	-2111.597142	-2111.669083	1.18
conf_7.log	-2111.599282	-2111.671765	20.17
conf_8.log	-2111.599461	-2111.671961	24.84
conf_9.log	-2111.599376	-2111.671904	23.38
conf_10.log	-2111.593742	-2111.665131	0.02

Boltzmann-Averaged Enthalpy (H) : -2111.599268 Hartree

|Boltzmann-Averaged Free Energy (G) : -2111.671702 Hartree

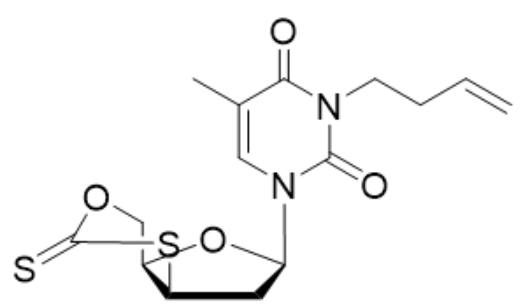

Structure	H (Hartree)	qh-G (Hartree)	Pop (%)
conf_1.log	-1788.622991	-1788.689895	20.01
conf_2.log	-1788.622990	-1788.689895	20.01
conf_3.log	-1788.621863	-1788.689372	11.50
conf_4.log	-1788.621981	-1788.689371	11.49
conf_5.log	-1788.620418	-1788.687785	2.14
conf_6.log	-1788.622523	-1788.689872	19.54
conf_7.log	-1788.618713	-1788.685299	0.15
conf_8.log	-1788.621025	-1788.688882	6.84
conf_9.log	-1788.620960	-1788.689061	8.27
conf_10.log	-1788.616091	-1788.684131	0.04

Boltzmann-Averaged Enthalpy (H) : -1788.622286 Hartree

Boltzmann-Averaged Free Energy (G) : -1788.689577 Hartree

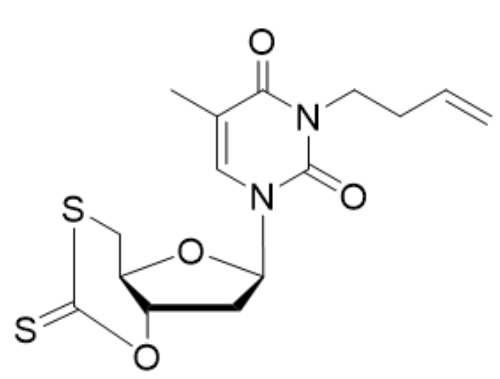

Structure	H (Hartree)	qh-G (Hartree)	Pop (%)
conf_1.log	-1788.615532	-1788.686954	10.42
conf_2.log	-1788.615712	-1788.686942	10.29
conf_3.log	-1788.615715	-1788.686943	10.31
conf_4.log	-1788.615712	-1788.686942	10.29
conf_5.log	-1788.615528	-1788.686954	10.42
conf_6.log	-1788.615710	-1788.686939	10.27
conf_7.log	-1788.615450	-1788.686784	8.71
conf_8.log	-1788.615711	-1788.686940	10.27
conf_9.log	-1788.615713	-1788.686942	10.30
conf_10.log	-1788.615452	-1788.686784	8.71

Boltzmann-Averaged Enthalpy (H) : -1788.615629 Hartree

Boltzmann-Averaged Free Energy (G) : -1788.686916 Hartree

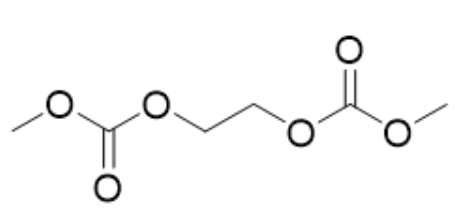

Structure	H (Hartree)	qh-G (Hartree)	Pop (%)
conf_1_opt.log	-685.674648	-685.727243	34.27
conf_2_opt.log	-685.673756	-685.726370	13.59
conf_3_opt.log	-685.673117	-685.726424	14.40
conf_4_opt.log	-685.673115	-685.726423	14.38
conf_5_opt.log	-685.673115	-685.726422	14.36
conf_6_opt.log	-685.670957	-685.723542	0.68
conf_7_opt.log	-685.670919	-685.724432	1.75
conf_8_opt.log	-685.670920	-685.724433	1.75
conf_9_opt.log	-685.672573	-685.724974	3.10
conf_10_opt.log	-685.670917	-685.724423	1.73

Boltzmann-Averaged Enthalpy (H) : -685.673582 Hartree

Boltzmann-Averaged Free Energy (G) : -685.726528 Hartree

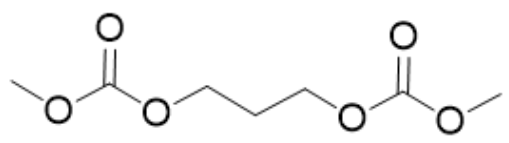

Structure	H (Hartree)	qh-G (Hartree)	Pop (%)
conf_1_opt.log	-724.953590	-725.007996	3.38
conf_2_opt.log	-724.953357	-725.008841	8.26
conf_3_opt.log	-724.951520	-725.006462	0.66
conf_4_opt.log	-724.955231	-725.010452	45.53
conf_5_opt.log	-724.953339	-725.008136	3.91
conf_6_opt.log	-724.949647	-725.004388	0.07
conf_7_opt.log	-724.953339	-725.008137	3.92
conf_8_opt.log	-724.950134	-725.005255	0.19
conf_9_opt.log	-724.953473	-725.009525	17.05
conf_10_opt.log	-724.953471	-725.009524	17.04

Boltzmann-Averaged Enthalpy (H) : -724.954235 Hartree
Boltzmann-Averaged Free Energy (G) : -725.009698 Hartree

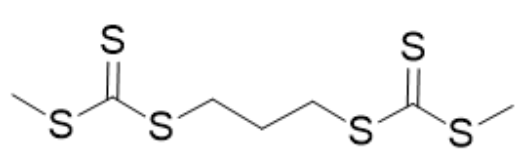

Structure	H (Hartree)	qh-G (Hartree)	Pop (%)
conf_1_opt.log	-2662.747035	-2662.809871	2.87
conf_2_opt.log	-2662.745010	-2662.807176	0.17
conf_3_opt.log	-2662.748667	-2662.811794	21.97
conf_4_opt.log	-2662.747772	-2662.810667	6.66
conf_5_opt.log	-2662.746604	-2662.809025	1.17
conf_6_opt.log	-2662.748995	-2662.812156	32.23
conf_7_opt.log	-2662.748251	-2662.812054	28.94
conf_8_opt.log	-2662.747075	-2662.809896	2.94
conf_9_opt.log	-2662.746196	-2662.809271	1.52
conf_10_opt.log	-2662.746198	-2662.809274	1.52

Boltzmann-Averaged Enthalpy (H) : -2662.748394 Hartree
Boltzmann-Averaged Free Energy (G) : -2662.811683 Hartree

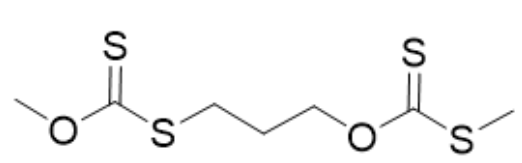

Structure	H (Hartree)	qh-G (Hartree)	Pop (%)
conf_1_opt.log	-2016.790574	-2016.850269	23.18
conf_2_opt.log	-2016.788288	-2016.847766	1.64
conf_3_opt.log	-2016.784669	-2016.844128	0.03
conf_4_opt.log	-2016.790289	-2016.850510	29.95
conf_5_opt.log	-2016.784671	-2016.844128	0.03
conf_6_opt.log	-2016.787862	-2016.848157	2.48
conf_7_opt.log	-2016.782596	-2016.842583	0.01
conf_8_opt.log	-2016.789347	-2016.849781	13.83
conf_9_opt.log	-2016.781218	-2016.840755	0.00
conf_10_opt.log	-2016.789724	-2016.850476	28.86

Boltzmann-Averaged Enthalpy (H) : -2016.789964 Hartree
Boltzmann-Averaged Free Energy (G) : -2016.850235 Hartree

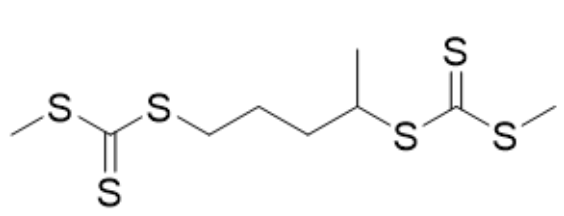

Structure	H (Hartree)	qh-G (Hartree)	Pop (%)
conf_1_opt.log	-2741.306916	-2741.374739	18.87
conf_2_opt.log	-2741.307667	-2741.375647	49.36
conf_3_opt.log	-2741.304771	-2741.373643	5.91
conf_4_opt.log	-2741.306206	-2741.374276	11.56
conf_5_opt.log	-2741.304827	-2741.372304	1.43
conf_6_opt.log	-2741.305082	-2741.372756	2.31
conf_7_opt.log	-2741.305514	-2741.373912	7.86
conf_8_opt.log	-2741.304646	-2741.372464	1.70
conf_9_opt.log	-2741.303312	-2741.371793	0.83
conf_10_opt.log	-2741.302281	-2741.370295	0.17

Boltzmann-Averaged Enthalpy (H) : -2741.306819 Hartree
Boltzmann-Averaged Free Energy (G) : -2741.374853 Hartree

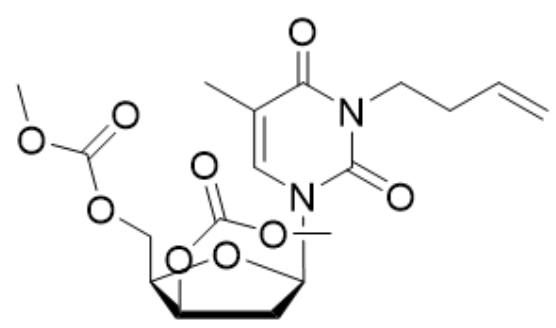

Structure	H (Hartree)	qh-G (Hartree)	Pop (%)
conf_1_opt.log	-1486.137052	-1486.221627	24.44
conf_2_opt.log	-1486.136857	-1486.220911	11.45
conf_3_opt.log	-1486.133496	-1486.217895	0.47
conf_4_opt.log	-1486.136008	-1486.221288	17.06
conf_5_opt.log	-1486.133594	-1486.217574	0.33
conf_6_opt.log	-1486.133123	-1486.218320	0.74
conf_7_opt.log	-1486.137054	-1486.221631	24.53
conf_8_opt.log	-1486.135088	-1486.221262	16.61
conf_9_opt.log	-1486.132431	-1486.217192	0.22
conf_10_opt.log	-1486.134252	-1486.219953	4.15

🏆 Boltzmann-Averaged Enthalpy (H) : -1486.136342 Hartree
🏆 Boltzmann-Averaged Free Energy (G) : -1486.221293 Hartree

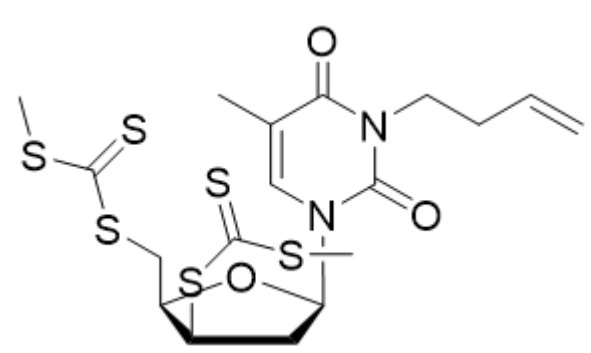

Structure	H (Hartree)	qh-G (Hartree)	Pop (%)
conf_1_opt.log	-3423.928084	-3424.020354	49.82
conf_2_opt.log	-3423.925791	-3424.017320	2.00
conf_3_opt.log	-3423.925463	-3424.017072	1.54
conf_4_opt.log	-3423.926881	-3424.018308	5.70
conf_5_opt.log	-3423.926329	-3424.018610	7.85
conf_6_opt.log	-3423.926327	-3424.018604	7.81
conf_7_opt.log	-3423.927086	-3424.019344	17.09
conf_8_opt.log	-3423.925447	-3424.017708	3.02
conf_9_opt.log	-3423.925451	-3424.017718	3.06
conf_10_opt.log	-3423.924896	-3424.017365	2.10

🏆 Boltzmann-Averaged Enthalpy (H) : -3423.927256 Hartree
🏆 Boltzmann-Averaged Free Energy (G) : -3424.019456 Hartree

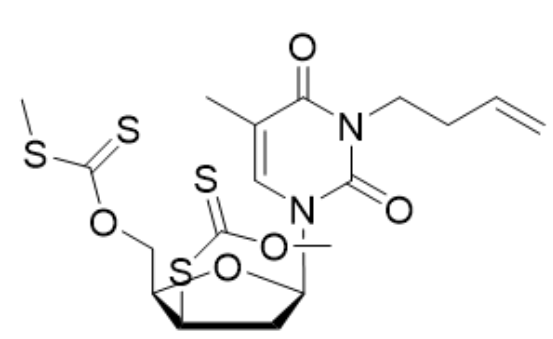

Structure	H (Hartree)	qh-G (Hartree)	Pop (%)
conf_1.log	-2777.978883	-2778.065865	19.18
conf_2.log	-2777.976888	-2778.064257	3.49
conf_4.log	-2777.978881	-2778.065859	19.05
conf_5.log	-2777.979021	-2778.066021	22.62
conf_6.log	-2777.977449	-2778.064292	3.62
conf_7.log	-2777.977448	-2778.064284	3.59
conf_8.log	-2777.977016	-2778.064351	3.86
conf_9.log	-2777.977898	-2778.065244	9.93
conf_10.log	-2777.978086	-2778.065611	14.65

🏆 Boltzmann-Averaged Enthalpy (H) : -2777.978454 Hartree
🏆 Boltzmann-Averaged Free Energy (G) : -2778.065572 Hartree

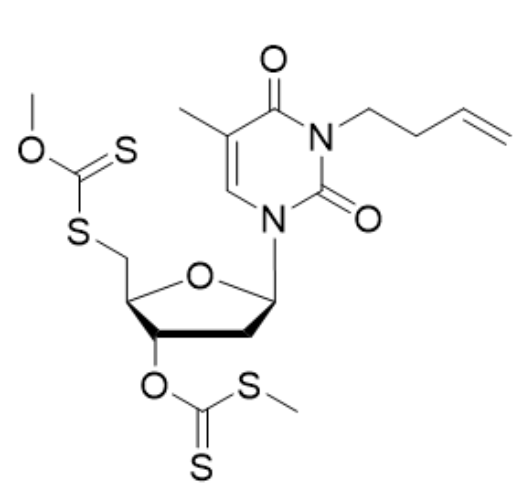

Structure	H (Hartree)	qh-G (Hartree)	Pop (%)
conf_1_opt.log	-2777.975562	-2778.065001	10.76
conf_2_opt.log	-2777.974140	-2778.064083	4.07
conf_3_opt.log	-2777.975995	-2778.066452	49.99
conf_4_opt.log	-2777.974393	-2778.064604	7.06
conf_5_opt.log	-2777.974859	-2778.064548	6.65
conf_6_opt.log	-2777.974134	-2778.064384	5.59
conf_7_opt.log	-2777.974714	-2778.064519	6.46
conf_8_opt.log	-2777.974714	-2778.064522	6.48
conf_9_opt.log	-2777.973798	-2778.063670	2.63
conf_10_opt.log	-2777.971882	-2778.061671	0.32

🏆 Boltzmann-Averaged Enthalpy (H) : -2777.975344 Hartree
🏆 Boltzmann-Averaged Free Energy (G) : -2778.065489 Hartree

Appendix 2. Ensemble average of enthalpies and free energies of each conformation under M062X/6-31++G(d,p) level.

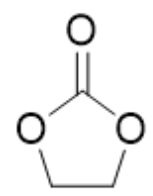


```
Structure                  H (Hartree)     qh-G (Hartree)  Pop (%)
-------------------------------------------------------------------
5carbonate_best.log        -342.211725     -342.241787     100.00
-------------------------------------------------------------------
Boltzmann-Averaged Enthalpy (H)    : -342.211725 Hartree
Boltzmann-Averaged Free Energy (G) : -342.241787 Hartree
-------------------------------------------------------------------
```

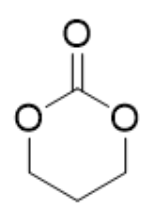


```
Structure                  H (Hartree)     qh-G (Hartree)  Pop (%)
-------------------------------------------------------------------
6carbonate_best.log        -381.477257     -381.511258     100.00
-------------------------------------------------------------------
Boltzmann-Averaged Enthalpy (H)    : -381.477257 Hartree
Boltzmann-Averaged Free Energy (G) : -381.511258 Hartree
-------------------------------------------------------------------
```

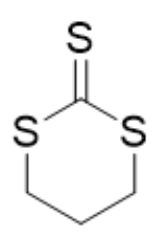


```
Structure                  H (Hartree)     qh-G (Hartree)  Pop (%)
-------------------------------------------------------------------
conf_1.log                 -1350.340617    -1350.378395    22.71
conf_2.log                 -1350.341539    -1350.379551    77.29
-------------------------------------------------------------------
Boltzmann-Averaged Enthalpy (H)    : -1350.341330 Hartree
Boltzmann-Averaged Free Energy (G) : -1350.379288 Hartree
-------------------------------------------------------------------
```

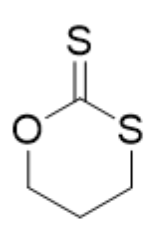


```
Structure                  H (Hartree)     qh-G (Hartree)  Pop (%)
-------------------------------------------------------------------
6xanthate_best.log         -1027.376269    -1027.412840    100.00
-------------------------------------------------------------------
Boltzmann-Averaged Enthalpy (H)    : -1027.376269 Hartree
Boltzmann-Averaged Free Energy (G) : -1027.412840 Hartree
-------------------------------------------------------------------
```

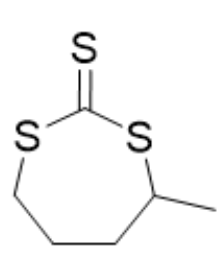


```
Structure                  H (Hartree)     qh-G (Hartree)  Pop (%)
-------------------------------------------------------------------
conf_1_opt.log             -1428.872594    -1428.915822    56.55
conf_2_opt.log             -1428.871864    -1428.914683    16.93
conf_3_opt.log             -1428.871353    -1428.914351    11.91
conf_4_opt.log             -1428.871129    -1428.914403    12.59
conf_5_opt.log             -1428.869801    -1428.912658    1.98
conf_6_opt.log             -1428.865892    -1428.908871    0.04
-------------------------------------------------------------------
Boltzmann-Averaged Enthalpy (H)    : -1428.872081 Hartree
Boltzmann-Averaged Free Energy (G) : -1428.915210 Hartree
-------------------------------------------------------------------
```

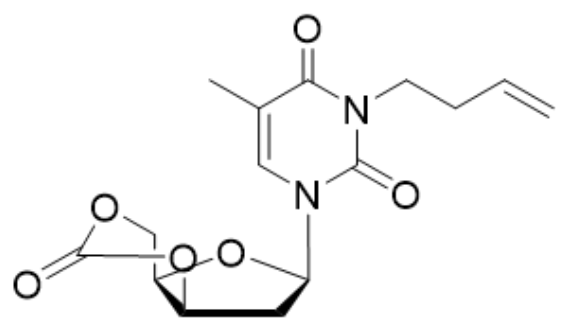

Structure	H (Hartree)	qh-G (Hartree)	Pop (%)
conf_1.log	-1142.587832	-1142.652271	9.88
conf_2.log	-1142.585210	-1142.649926	0.82
conf_3.log	-1142.587627	-1142.653508	36.64
conf_4.log	-1142.587631	-1142.653472	35.24
conf_5.log	-1142.586818	-1142.651751	5.70
conf_6.log	-1142.586826	-1142.651757	5.73
onf_7.log	-1142.584976	-1142.649680	0.64
onf_8.log	-1142.585022	-1142.650742	1.96
conf_9.log	-1142.584939	-1142.650607	1.70
conf_10.log	-1142.584939	-1142.650608	1.70

M06-2X Boltzmann-Averaged Enthalpy (H) : -1142.587378 Hartree

M06-2X Boltzmann-Averaged Free Energy (G) : -1142.652966 Hartree

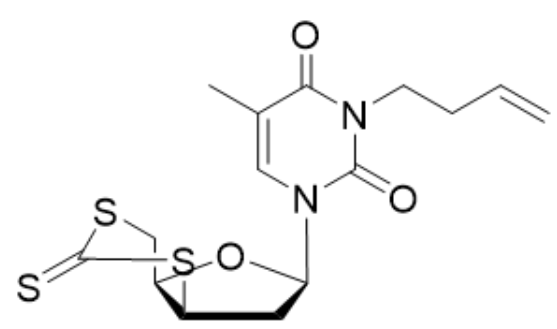

Structure	H (Hartree)	qh-G (Hartree)	Pop (%)
conf_1.log	-2111.448820	-2111.517166	1.96
conf_2.log	-2111.450169	-2111.518598	8.94
conf_3.log	-2111.447765	-2111.516279	0.77
conf_4.log	-2111.449548	-2111.518174	5.71
conf_5.log	-2111.449884	-2111.519396	20.82
conf_6.log	-2111.448291	-2111.517213	2.06
conf_7.log	-2111.449785	-2111.519249	17.83
conf_8.log	-2111.449928	-2111.519409	21.12
conf_9.log	-2111.449885	-2111.519393	20.76
conf_10.log	-2111.444952	-2111.513322	0.03

M06-2X Boltzmann-Averaged Enthalpy (H) : -2111.449810 Hartree

M06-2X Boltzmann-Averaged Free Energy (G) : -2111.519116 Hartree

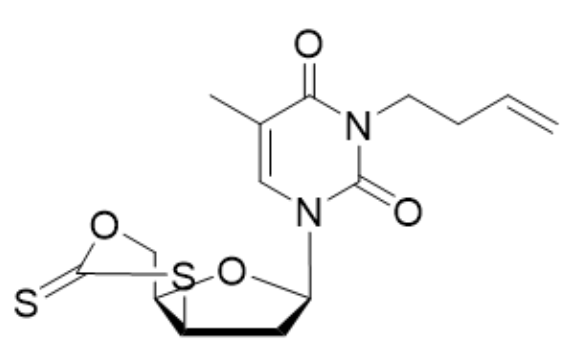

Structure	H (Hartree)	qh-G (Hartree)	Pop (%)
conf_1.log	-1788.486270	-1788.553173	20.94
conf_2.log	-1788.486269	-1788.553173	20.94
conf_3.log	-1788.485232	-1788.552741	13.24
conf_4.log	-1788.485330	-1788.552720	12.95
conf_5.log	-1788.483503	-1788.550871	1.83
conf_6.log	-1788.485624	-1788.552973	16.95
conf_7.log	-1788.482031	-1788.548618	0.17
conf_8.log	-1788.484120	-1788.551976	5.89
conf_9.log	-1788.484046	-1788.552147	7.06
conf_10.log	-1788.478819	-1788.546859	0.03

Boltzmann-Averaged Enthalpy (H) : -1788.485558 Hartree

Boltzmann-Averaged Free Energy (G) : -1788.552829 Hartree

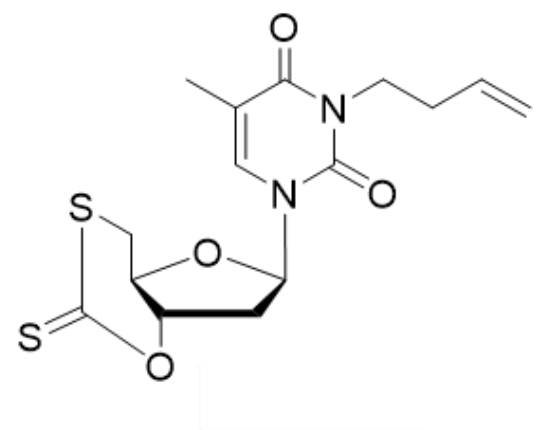

Structure	H (Hartree)	qh-G (Hartree)	Pop (%)
conf_1.log	-1788.479224	-1788.547626	9.47
conf_2.log	-1788.479543	-1788.547753	10.83
conf_3.log	-1788.479546	-1788.547755	10.85
conf_4.log	-1788.479542	-1788.547753	10.83
conf_5.log	-1788.479221	-1788.547628	9.48
conf_6.log	-1788.479540	-1788.547751	10.80
conf_7.log	-1788.479158	-1788.547472	8.04
conf_8.log	-1788.479541	-1788.547751	10.81
conf_9.log	-1788.479543	-1788.547753	10.83
conf_10.log	-1788.479159	-1788.547472	8.05

Boltzmann-Averaged Enthalpy (H) : -1788.479420 Hartree
Boltzmann-Averaged Free Energy (G) : -1788.547684 Hartree

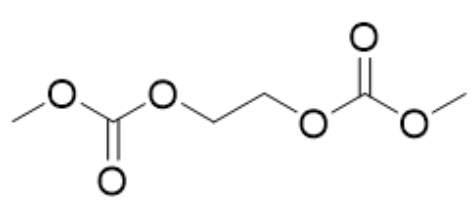

Structure	H (Hartree)	qh-G (Hartree)	Pop (%)
conf_1_opt.log	-685.610066	-685.659642	57.33
conf_2_opt.log	-685.608913	-685.658509	17.25
conf_3_opt.log	-685.607274	-685.657562	6.33
conf_4_opt.log	-685.607271	-685.657560	6.32
conf_5_opt.log	-685.607271	-685.657559	6.31
conf_6_opt.log	-685.606332	-685.655898	1.09
conf_7_opt.log	-685.605012	-685.655506	0.72
conf_8_opt.log	-685.605012	-685.655507	0.72
conf_9_opt.log	-685.607542	-685.656924	3.22
conf_10_opt.log	-685.605013	-685.655500	0.71

Boltzmann-Averaged Enthalpy (H) : -685.609107 Hartree
Boltzmann-Averaged Free Energy (G) : -685.658835 Hartree

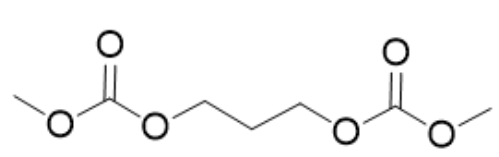

Structure	H (Hartree)	qh-G (Hartree)	Pop (%)
conf_1_opt.log	-724.878407	-724.929795	6.04
conf_2_opt.log	-724.877726	-724.930190	9.18
conf_3_opt.log	-724.876297	-724.928220	1.14
conf_4_opt.log	-724.879558	-724.931761	48.45
conf_5_opt.log	-724.878497	-724.930274	10.04
conf_6_opt.log	-724.874727	-724.926449	0.17
conf_7_opt.log	-724.878501	-724.930279	10.09
conf_8_opt.log	-724.873329	-724.925432	0.06
conf_9_opt.log	-724.876955	-724.929989	7.42
conf_10_opt.log	-724.876954	-724.929988	7.41

Boltzmann-Averaged Enthalpy (H) : -724.878672 Hartree
Boltzmann-Averaged Free Energy (G) : -724.930883 Hartree

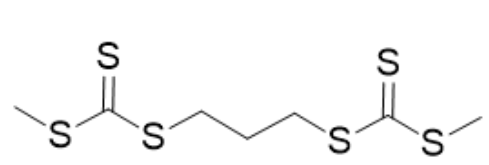

Structure	H (Hartree)	qh-G (Hartree)	Pop (%)
conf_1_opt.log	-2662.597487	-2662.657304	5.74
conf_2_opt.log	-2662.595375	-2662.654523	0.30
conf_3_opt.log	-2662.598738	-2662.658846	29.42
conf_4_opt.log	-2662.597357	-2662.657234	5.34
conf_5_opt.log	-2662.596875	-2662.656277	1.94
conf_6_opt.log	-2662.598983	-2662.659125	39.53
conf_7_opt.log	-2662.597362	-2662.658146	14.03
conf_8_opt.log	-2662.596383	-2662.656186	1.76
conf_9_opt.log	-2662.595567	-2662.655623	0.97
conf_10_opt.log	-2662.595568	-2662.655625	0.97

Boltzmann-Averaged Enthalpy (H) : -2662.598347 Hartree
Boltzmann-Averaged Free Energy (G) : -2662.658511 Hartree

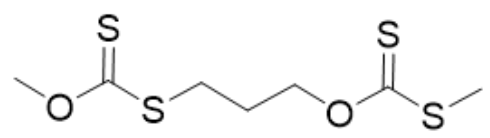

Structure	H (Hartree)	qh-G (Hartree)	Pop (%)
conf_1_opt.log	-2016.666598	-2016.723273	47.96
conf_2_opt.log	-2016.664026	-2016.720485	2.50
conf_3_opt.log	-2016.661031	-2016.717471	0.10
conf_4_opt.log	-2016.665635	-2016.722838	30.24
conf_5_opt.log	-2016.661029	-2016.717467	0.10
conf_6_opt.log	-2016.663014	-2016.720290	2.04
conf_7_opt.log	-2016.658105	-2016.715073	0.01
conf_8_opt.log	-2016.663787	-2016.721202	5.35
conf_9_opt.log	-2016.656391	-2016.712909	0.00
conf_10_opt.log	-2016.664208	-2016.721941	11.70

Boltzmann-Averaged Enthalpy (H) : -2016.665727 Hartree
Boltzmann-Averaged Free Energy (G) : -2016.722732 Hartree

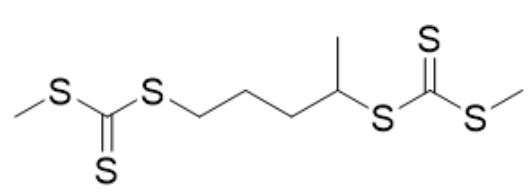

Structure	H (Hartree)	qh-G (Hartree)	Pop (%)
conf_1_opt.log	-2741.137109	-2741.201914	35.27
conf_2_opt.log	-2741.136652	-2741.201612	25.63
conf_3_opt.log	-2741.134396	-2741.200248	6.04
conf_4_opt.log	-2741.136197	-2741.201248	17.43
conf_5_opt.log	-2741.134936	-2741.199395	2.45
conf_6_opt.log	-2741.134851	-2741.199506	2.75
conf_7_opt.log	-2741.135187	-2741.200566	8.46
conf_8_opt.log	-2741.133559	-2741.198358	0.82
conf_9_opt.log	-2741.133166	-2741.198628	1.09
conf_10_opt.log	-2741.130948	-2741.195944	0.06

Boltzmann-Averaged Enthalpy (H) : -2741.136315 Hartree
Boltzmann-Averaged Free Energy (G) : -2741.201309 Hartree

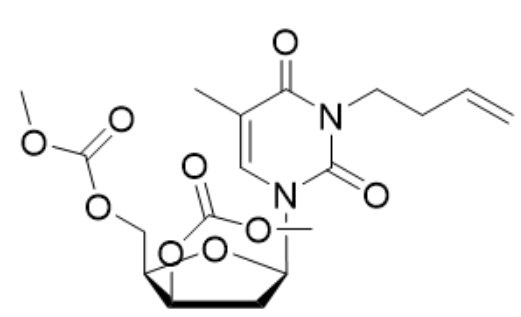

Structure	H (Hartree)	qh-G (Hartree)	Pop (%)
conf_1_opt.log	-1485.989004	-1486.070560	29.24
conf_2_opt.log	-1485.988217	-1486.069253	7.32
conf_3_opt.log	-1485.985536	-1486.066916	0.62
conf_4_opt.log	-1485.987733	-1486.069994	16.05
conf_5_opt.log	-1485.983029	-1486.063991	0.03
conf_6_opt.log	-1485.984196	-1486.066374	0.35
conf_7_opt.log	-1485.989007	-1486.070565	29.40
conf_8_opt.log	-1485.986472	-1486.069628	10.89
conf_9_opt.log	-1485.984261	-1486.066003	0.23
conf_10_opt.log	-1485.986362	-1486.069045	5.87

Boltzmann-Averaged Enthalpy (H) : -1485.988262 Hartree
Boltzmann-Averaged Free Energy (G) : -1486.070135 Hartree

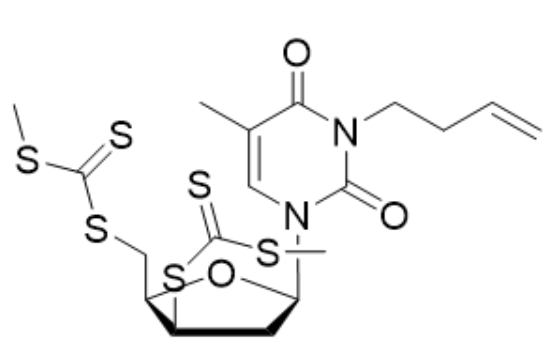

Structure	H (Hartree)	qh-G (Hartree)	Pop (%)
conf_1_opt.log	-3423.707229	-3423.796481	61.17
conf_2_opt.log	-3423.703778	-3423.792288	0.72
conf_3_opt.log	-3423.703109	-3423.791699	0.39
conf_4_opt.log	-3423.705476	-3423.793885	3.91
conf_5_opt.log	-3423.705135	-3423.794397	6.73
conf_6_opt.log	-3423.705136	-3423.794395	6.72
conf_7_opt.log	-3423.705928	-3423.795167	15.22
conf_8_opt.log	-3423.703930	-3423.793173	1.84
conf_9_opt.log	-3423.703934	-3423.793183	1.86
conf_10_opt.log	-3423.703497	-3423.792948	1.45

Boltzmann-Averaged Enthalpy (H) : -3423.706464 Hartree
Boltzmann-Averaged Free Energy (G) : -3423.795677 Hartree

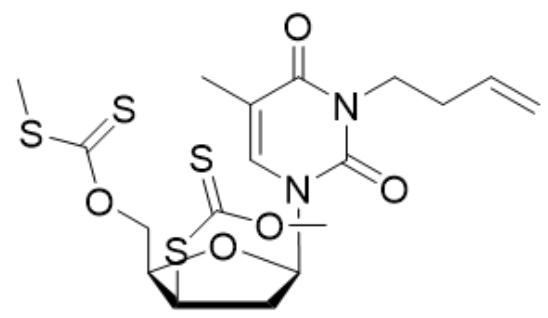

Structure	H (Hartree)	qh-G (Hartree)	Pop (%)
conf_1.log	-2777.780898	-2777.867880	10.17
conf_2.log	-2777.778625	-2777.865994	1.38
conf_4.log	-2777.780895	-2777.867873	10.10
conf_5.log	-2777.781328	-2777.868329	16.36
conf_6.log	-2777.780385	-2777.867228	5.10
conf_7.log	-2777.780384	-2777.867221	5.06
conf_8.log	-2777.778841	-2777.866175	1.67
conf_9.log	-2777.781235	-2777.868581	21.38
conf_10.log	-2777.781337	-2777.868862	28.78

🏆 Boltzmann-Averaged Enthalpy (H) : -2777.781049 Hartree

🏆 Boltzmann-Averaged Free Energy (G) : -2777.868264 Hartree

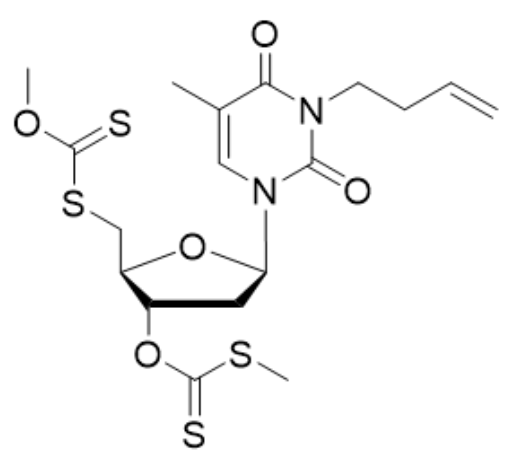

Structure	H (Hartree)	qh-G (Hartree)	Pop (%)
conf_1_opt.log	-2777.777895	-2777.864315	12.63
conf_2_opt.log	-2777.775862	-2777.862785	2.50
conf_3_opt.log	-2777.777971	-2777.865409	40.23
conf_4_opt.log	-2777.776675	-2777.863868	7.86
conf_5_opt.log	-2777.776906	-2777.863575	5.77
conf_6_opt.log	-2777.776456	-2777.863687	6.49
conf_7_opt.log	-2777.777416	-2777.864203	11.21
conf_8_opt.log	-2777.777417	-2777.864206	11.24
conf_9_opt.log	-2777.775384	-2777.862237	1.40
conf_10_opt.log	-2777.774788	-2777.861559	0.68

🏆 Boltzmann-Averaged Enthalpy (H) : -2777.777465 Hartree

🏆 Boltzmann-Averaged Free Energy (G) : -2777.864526 Hartree

IV. References:

(1) Tsao, Y.-Y. T.; Wooley, K. L. Synthetic, Functional Thymidine-Derived Polydeoxyribonucleotide Analogues from a Six-Membered Cyclic Phosphoester. *J. Am. Chem. Soc.* **2017**, *139* (15), 5467-5473.

(2) López-Vidal, E. M.; Gregory, G. L.; Kociok-Köhn, G.; Buchard, A. Polymers from sugars and CS_2: synthesis and ring-opening polymerisation of sulfur-containing monomers derived from 2-deoxy-d-ribose and d-xylose. *Polym. Chem.* **2018**, *9* (13), 1577-1582.

(3) Pracht, P.; Bohle, F.; Grimme, S. Automated exploration of the low-energy chemical space with fast quantum chemical methods. *Phys. Chem. Chem. Phys.* **2020**, *22* (14), 7169-7192.

(4) Bannwarth, C.; Ehlert, S.; Grimme, S. GFN2-xTB—An Accurate and Broadly Parametrized Self-Consistent Tight-Binding Quantum Chemical Method with Multipole Electrostatics and Density-Dependent Dispersion Contributions. *Journal of Chemical Theory and Computation* **2019**, *15* (3), 1652-1671.

(5) Frisch, M. e.; Trucks, G.; Schlegel, H. B.; Scuseria, G.; Robb, M.; Cheeseman, J.; Scalmani, G.; Barone, V.; Petersson, G.; Nakatsuji, H. Gaussian 16. Gaussian, inc. Wallingford, CT: 2016.

(6) Luchini, G.; Alegre-Requena, J.V.; Funes-Ardoiz, I.; Paton, R. S. GoodVibes: automated thermochemistry for heterogeneous computational chemistry data. *F1000Research* **2020**, *9* (291), 1-14.

(7) Grimme, S. Supramolecular Binding Thermodynamics by Dispersion-Corrected Density Functional Theory. *Chem. Eur. J.* **2012**, *18* (32), 9955-9964.

(8) Vogdanis, L.; Martens, B.; Uchtmann, H.; Hensel, F.; Heitz, W. Synthetic and thermodynamic investigations in the polymerization of ethylene carbonate. *Makromol. Chem.* **1990**, *191* (3), 465-472.

(9) Matsuo, J.; Aoki, K.; Sanda, F.; Endo, T. Substituent Effect on the Anionic Equilibrium Polymerization of Six-Membered Cyclic Carbonates. *Macromolecules* **1998**, *31* (14), 4432-4438.

(10) Zhao, J.-Z.; Yue, T.-J.; Ren, B.-H.; Liu, Y.; Ren, W.-M.; Lu, X.-B. Recyclable Sulfur-Rich Polymers with Enhanced Thermal, Mechanical, and Optical Performance. *Macromolecules* **2022**, *55* (19), 8651-8658.

(11) Gregory, G. L.; Hierons, E. M.; Kociok-Köhn, G.; Sharma, R. I.; Buchard, A. CO_2-Driven stereochemical inversion of sugars to create thymidine-based polycarbonates by ring-opening polymerisation. *Polym. Chem.* **2017**, *8* (10), 1714-1721.
